# Macrophages tune NK cell IFNγ production through direct lipid transfer

**DOI:** 10.1038/s41467-026-76444-0

**Published:** 2026-08-08

**Authors:** Cathal Keane, Shauna Mayock, Carrie Corkish, Ella Rogerson, Cristhiane Favero de Aguiar, Ya Gao, Sarah Kenny, Lorraine Brennan, Suzanne M. Cloonan, David K. Finlay

**Affiliations:** 1 School of Biochemistry and Immunology, Trinity Biomedical Sciences Institute, Trinity College Dublin, Dublin, Ireland; 2 School of Agriculture and Food Science, Conway Institute, Institute of Food and Health, University College Dublin, Dublin, Ireland; 3 School of Medicine, Trinity Biomedical Sciences Institute, Trinity College Dublin, Dublin, Ireland; 4 Division of Pulmonary and Critical Care Medicine, Joan and Sanford I. Weill Department of Medicine, Weill Cornell Medicine, New York, NY USA; 5 School of Pharmacy and Pharmaceutical Sciences, Trinity Biomedical Sciences Institute, Trinity College Dublin, Dublin, Ireland

**Keywords:** Innate immunity, Lipids, Metabolomics, Membrane trafficking

## Abstract

Natural killer (NK) cells are critical effectors of innate immunity, but their activity is strongly influenced by metabolic state. While intrinsic NK metabolism has been studied extensively, less is known about how surrounding immune cells shape NK cell function. Here, we identify a direct metabolic communication axis between macrophages and NK cells. Using co-culture and in vivo models, we show that lipopolysaccharide-stimulated macrophages induce lipid accumulation in NK cells that suppresses mTORC1 activity and the production of IFNγ. This lipid accumulation is visualised as increased lipid droplets content in NK cells, generated using fatty acids synthesised within the macrophages. Genetic and pharmacological approaches show that fatty acid transfer from macrophages to NK cells requires cell-cell contact and is associated with CD36 protein transfer via trogocytosis. Blocking fatty acid synthesis specifically in macrophages prevents lipid accumulation in NK cells and restores both mTORC1 activity and IFNγ production. These findings define a previously unrecognized mechanism of macrophage-NK cell cross-regulation, revealing how metabolic exchange constrains NK effector function and establishing a feedback circuit with implications for hyperinflammation and immunotherapy.

## Abstract (web summary)

The metabolic state of NK cells affects their innate immune function, and although cell-intrinsic pathways are better known, their cellular environment could provide cues that are less studied. Here the authors show that in coculture and mouse models, activated macrophages donate membrane lipids and CD36 protein via trogocytosis, which leads to the accumulation of lipid droplets and inhibition of mTORc1 and IFNγ production.

## Introduction

Natural killer (NK) cells are cytotoxic lymphocytes of the innate immune system with critical roles in early defence against virally infected, transformed or otherwise stressed cells, and contribute to the overall response to bacterial infection^1^. Their ability to act rapidly and in an antigen-independent manner makes NK cells attractive effectors in immunotherapy^2^. However, realising their full potential depends on understanding not just intrinsic activation signals but also how external cellular environments shape their metabolic and functional states^3^.

Over the past decade, studies of NK cell metabolism in isolated or simplified culture systems have delineated key fuel preferences and regulatory networks. Glucose uptake, glycolysis, oxidative phosphorylation (OXPHOS) and mitochondrial biogenesis are upregulated in response to cytokine stimulation (e.g. IL-12, IL-15 and IL-18), and regulators such as mTORC1 and sterol regulatory element-binding proteins (SREBPs) are required for metabolic reprogramming^4^. Recently, the non-canonical citrate-malate shuttle (CMS), involving SREBP target genes, including *Acly* and *Slc25a1*, has been shown to be critical for coupling glycolysis with OXPHOS in activated NK cells. These findings, however, derive primarily from studies in vitro where soluble cytokines dominate, and may not fully capture the complexity of tissue niches in vivo.

In vivo, NK cells reside within complex tissue microenvironments alongside diverse innate immune cells, among which macrophages are ubiquitous and highly plastic. Activated macrophages provide a variety of stimuli to NK cells: they secrete cytokines such as IL-12, IL-15 and type I interferons; express activating or inhibitory ligands (e.g. Rae-1, a NKG2D ligand); and engage in receptor–ligand interactions that can either amplify or suppress NK cytotoxicity and cytokine production^5,6^. Conversely, NK cell-derived IFN-γ and cell–cell interactions can in turn modulate macrophage activation states, contributing to feedback loops of immune stimulation or suppression^1,7^. Cell–cell contact, whether through receptor engagement or synapse formation, is often required for maximal NK activation by macrophages^8,9^. Moreover, intravital imaging has shown that NK cells form sustained, sequential interactions with myeloid cells in vivo, highlighting significant opportunities for functional crosstalk^10,11^. These sustained contacts can lead to the transfer of membrane fragments and associated proteins to NK cells through trogocytosis, with clear consequences for NK‑cell phenotype^12^. For example, NK‑cell acquisition of MHC class II from dendritic cells generates MHCII^+^ NK cells capable of suppressing CD4^+^ T‑cell response^13^.

While immunological cross-regulation between macrophages and NK cells is well studied, much less is known about how metabolism participates in this crosstalk, especially by means other than soluble cytokines. The tumour microenvironment (TME), obesity and infection are known to impose metabolic constraints upon immune cells and to impair NK cell functions. These metabolic stressors include hypoxia, nutrient deprivation and the accumulation of metabolites like lactate. Some studies show lipid accumulation in NK or T cells can reduce effector functionality^14–16^. In macrophages, lipid uptake, FAO and de novo lipogenesis are features underpinning macrophage polarisation and immune suppressive functions in the context of cancer^17^. However, direct metabolic interactions between macrophages and NK cells has remained largely unexplored.

Here, we reveal a previously uncharacterised metabolic communication axis between macrophages and NK cells. The data show that macrophages synthesise fatty acids (FA) de novo, which are transferred to NK cells alongside plasma membrane proteins, such as CD36, via direct cell contact. This acquisition of FA is associated with the increased expression of FA handling genes that facilitate storage in lipid droplets (LD). Critically, this macrophage-derived lipid transfer to NK cells suppresses mTORC1 signalling and IFN-γ cytokine output. Inhibition of FA synthase in macrophages rescued both mTORC1 activity and IFN-γ production in the interacting NK cells. Together, our findings propose a metabolic negative feedback mechanism by which macrophages limit sustained NK activation, with implications for tuning NK responses in inflammatory disease and for improving NK-based immunotherapies.

Understanding this macrophage-NK metabolic axis opens opportunities for therapeutic intervention: targeting lipid synthesis in macrophages, or modulating the transfer of lipid, may help enhance NK effector function when desirable, in cancer, or limit overactivation in the case of sepsis or systemic inflammation.

## Results

### Peritoneal NK cells activate during LPS-induced peritonitis

**Fig. 1:**
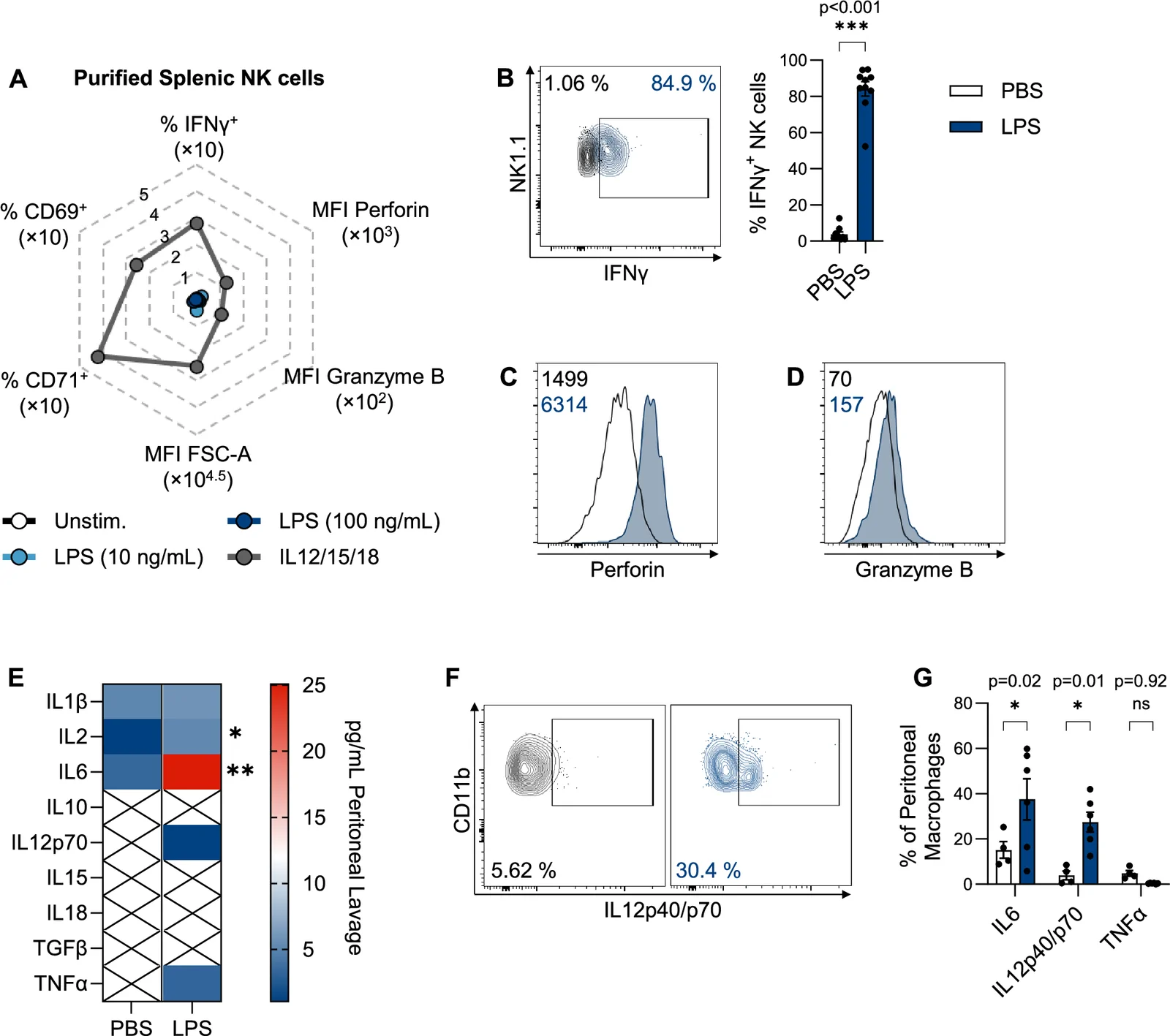
Peritoneal NK cells activate during LPS-induced peritonitis. **A** Splenic NK cells were MACS purified and stimulated with LPS (10 ng/mL, 100 ng/mL) for 24 h. Unstimulated NK cells were maintained in low-dose IL15 (2 ng/mL). NK cells were stimulated with IL12 (10 ng/mL), IL15 (10 ng/mL) and IL18 (50 ng/mL) as a positive control. IFNγ, perforin, granzyme B, FSC-A, CD71 and CD69 were used as measures of NK cell activation and were measured by flow cytometry. Data are the mean of four biological replicates. **B**–**G** C57BL/6 mice were administered LPS (2.4 mg/kg) or PBS by intraperitoneal injection. Twenty-four hours later, mice were sacrificed, and PECs were harvested by peritoneal lavage. **B**–**D** IFNγ, perforin and granzyme B expression by peritoneal NK cells was measured by flow cytometry. Data are mean ± SEM of *n* = 9 (PBS) and 10 (LPS) across three independent experiments. **E** Cytokine production in the peritoneal lavage was measured using a cytokine bead array. Data are the mean of six biological replicates across two independent experiments. **F**, **G** IL6, IL12p40/p70 and TNFα expression by peritoneal macrophages was measured by flow cytometry. Pooled data is mean ± SEM of *n* = 4 (PBS) and *n* = 6 (LPS) across two independent experiments. Statistical analysis was performed using unpaired two-tailed Welch’s t-tests (**B**, **E**, **G**).

To investigate NK cell activation in a hyperinflammatory environment in vivo, mice were injected with the TLR agonist, LPS, via intraperitoneal injection and NK cells analysed by flow cytometry after 24 h. The assumption was that NK cells would not respond directly to LPS challenge as they lack TLR expression (Immpres.co.uk, unpublished proteomic datasets), and NK activation would involve NK crosstalk with macrophages. Indeed, initial control experiments showed that murine splenic NK cells (called spNK hereafter) are not activated by LPS (10, 100 ng/mL) provided in vitro. The data showed no increase in the expression of activation markers, IFNγ or cytotoxic molecules compared to the positive control of cytokine-stimulated spNK cells (Fig. 1A). In contrast, NK cells from the peritoneum (called pNK hereafter) of LPS-challenged mice, analysed amongst peritoneal exudate cells (PECs), showed significant increases in the expression of IFNγ, perforin and granzyme B (Fig. 1B–D). Cytokine production in the peritoneal lavage was measured, showing a significant increase in the proinflammatory cytokines IL6 and IL2, confirming LPS-induced inflammation in this LPS-induced peritonitis model (Fig. 1E). Small peritoneal macrophages (SPMs) are the primary pro-inflammatory macrophage population present in the peritoneal cavity following LPS-challenge^1^. Analysis of cytokine production by peritoneal macrophages showed that these cells produce significant amounts of IL12, which alongside IL2 (Fig. 1E) is sufficient to induce a potent NK cell response (Fig. 1F)^18,19^.

### Increased metabolic activity of NK cells within inflamed peritoneum

**Fig. 2:**
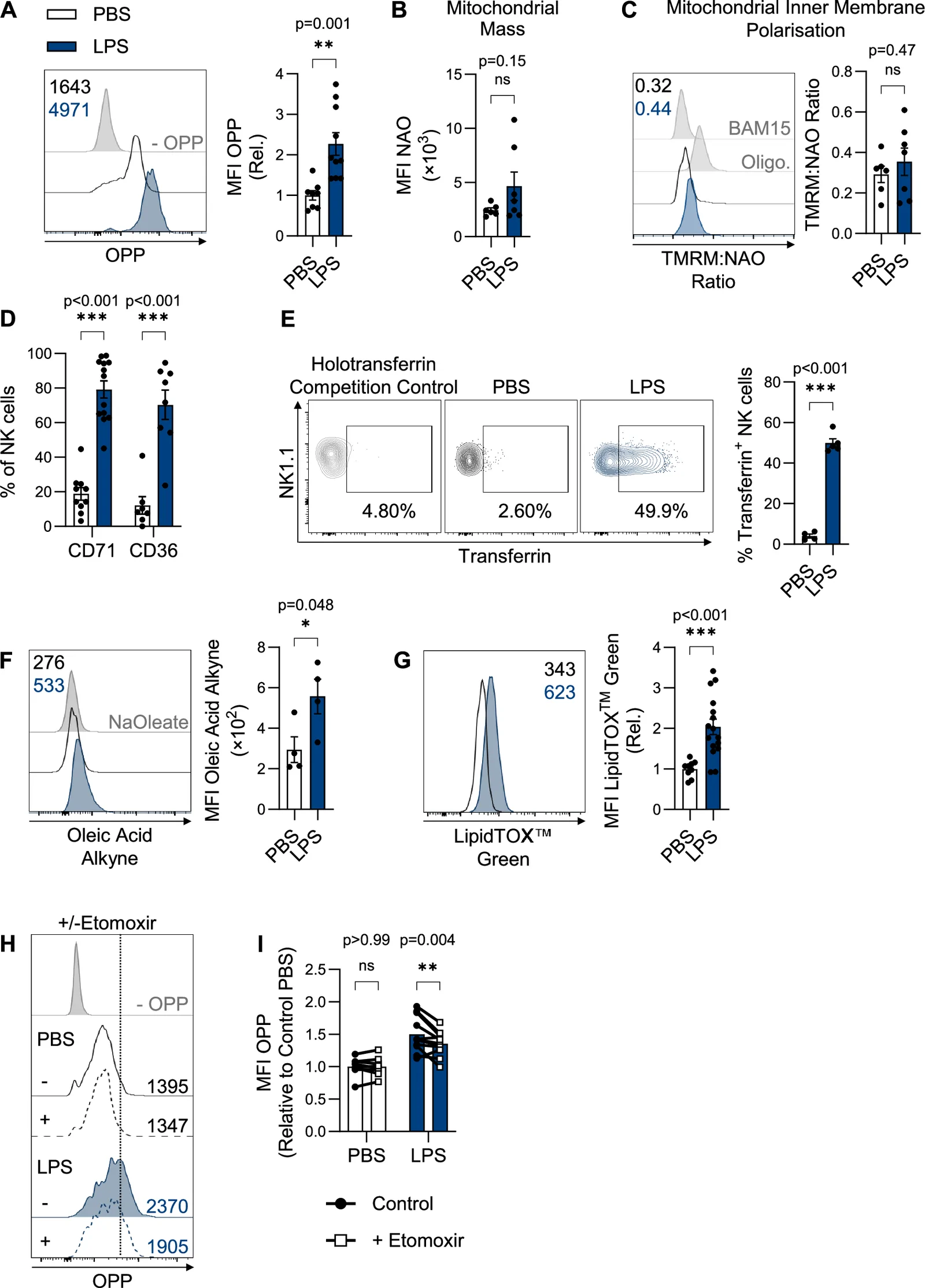
Increased metabolic activity of NK cells within inflamed peritoneum. C57BL/6 mice were administered LPS (2.4 mg/kg) or PBS by intraperitoneal injection. Twenty-four hours later, mice were sacrificed, and PECs were harvested by peritoneal lavage. **A** O-propargyl puromycin (OPP) (20 µM) incorporation into protein by PECs was measured by flow cytometry. Data are mean ± SEM of *n* = 8 (PBS) or 10 (LPS) across two independent experiments. **B**, **C** Nonylacridine orange (NAO) (100 nM) and TMRM (100 nM) staining of PECs was carried out before measurement by flow cytometry. FCCP and oligomycin were used as controls for TMRM measurements. Data are mean ± SEM of *n* = 6 (PBS) or 7 (LPS) across two independent experiments. **D** CD71 and CD36 expression were measured by flow cytometry. Data are mean ± SEM. For CD71 *n* = 10 (PBS) or 13 (LPS) across four independent experiments. For CD36 *n* = 7 (PBS) or 8 (LPS) across three independent experiments. **E** Transferrin-AF647 (5 µg/mL) uptake by PECs was performed before measurement by flow cytometry. Unlabelled holotransferrin (500 µg/mL) was used as a competition control. Pooled data are mean ± SEM of *n* = 4. **F** Oleic acid alkyne (6 µM) uptake by PECs was measured by flow cytometry. Sodium oleate (NaOleate) (60 µM) was used as a competition control. Data are mean ± SEM of *n* = 4. **G** LipidTOX™ Green staining of PECs was measured by flow cytometry. Pooled data is mean ± SEM of *n* = 9 (PBS) or *n* = 16 (LPS) across three independent experiments. **H**, **I** PECs were pre-treated with etomoxir (5 µM) for 10 min prior to 30 min O-propargyl puromycin (OPP) (20 µM) incorporation into protein before measurement by flow cytometry. Pooled data are mean ± SEM of *n* = 8 (PBS) or 11 (LPS) across two independent experiments. Statistical analysis was performed using unpaired two-tailed t-tests (**A**–**G**) or a two-way ANOVA repeated measures ANOVA with Šidák’s post-test (**I**).

To further understand the activation of pNK cells, metabolic parameters were measured. The incorporation of puromycin into newly translated proteins was used as a surrogate measurement of ATP consumption, based on the fact that this anabolic process is one of the most ATP-depending processes that occurs in the cell. The data showed that following LPS-challenge pNK cells have significantly increased rates of protein translation (Fig. 2A), but showed no increases in mitochondrial mass (Fig. 2B) or mitochondrial inner membrane polarisation (Fig. 2C). Expression of nutrient receptors on pNK cells was measured, revealing significantly increased expression of the transferrin receptor, CD71 and the lipid scavenging receptor, CD36 (Fig. 2D). The increase in CD71 expression was mirrored in a significant increase in uptake of iron-bound transferrin (Fig. 2E). Iron is an important factor to support metabolic processes in NK cells, including OXPHOS and DNA replication. The increase in CD36 expression, a scavenging receptor linked to the uptake of lipid species, including long-chain FA (LCFA), suggests an increase in fatty acid uptake. Indeed, there was an increase in uptake of a oleic acid-alkyne molecule, which was visualised by flow cytometry following the biorthogonal chemistry-mediated addition of a fluorophore (Fig. 2F). This oleic acid-alkyne is taken up through physiological routes because uptake was blocked by the addition of excess sodium oleate that competes for uptake into the pNK cell (Fig. 2F and Supplementary Fig. 2D). pNK cells from LPS-challenged mice also increased their accumulation of intracellular neutral lipids as indicated by increased LipidTOX™ Green staining (Fig. 2G). This dye stains neutral lipids such as di- and tri-acyl-glycerides (DAGs and TAGs) and cholesteryl esters, indicating a lipid storage phenotype in pNK cells, which is not commonly reported for activated NK cells; increased lipid storage has only been reported in NK cells responding to viral infections^20,21^. To assess whether stored lipids support energy metabolism, pNK cells were pre-treated with etomoxir for 10 min, an inhibitor of the carnitine shuttle system required for FAO, prior to measuring rates of protein translation. Following LPS-challenge, protein translation in pNK cells was acutely sensitive to inhibition of FAO (Fig. 2H), indicating that LCFA are important contributors to pNK cell energy metabolism. Nevertheless, 4 h exposure to etomoxir was not sufficient to impair effector molecule production by pNK cells (Supplementary Fig. 2A–C).

### Direct macrophage–NK cell interactions suppress maximal pNK cell responses

**Fig. 3:**
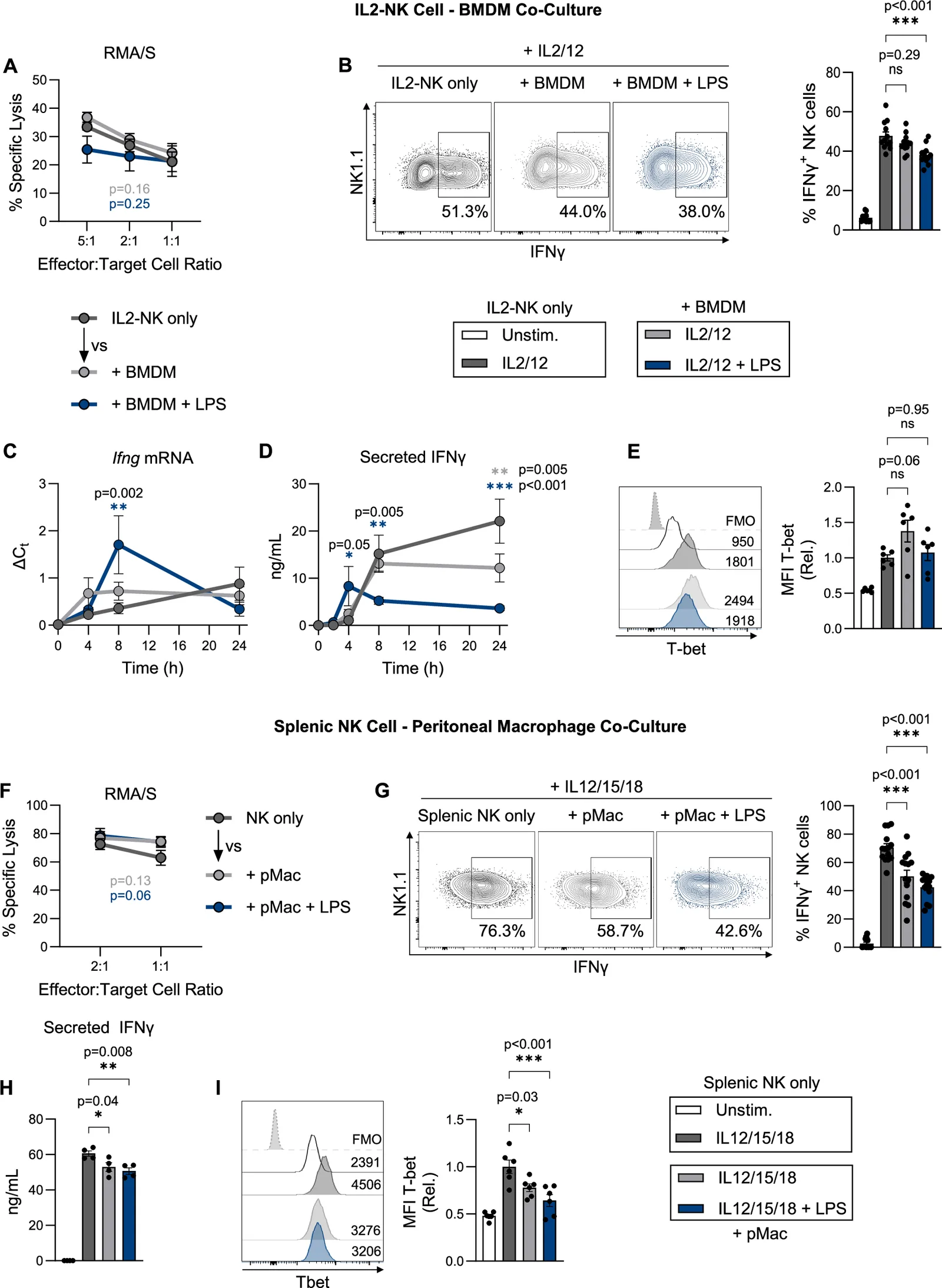
Direct interactions between macrophages and NK cells limit NK cell effector functions. **A**–**E** BMDMs were stimulated with LPS (10 ng/mL) or left unstimulated for 6 h. Then IL2-NK cells were directly co-cultured with BMDMs for 18 h in the presence of NK cell-activating cytokines IL2 (20 ng/mL) and IL12 (10 ng/mL). **F**–**I** NK cells were MACS purified from the spleen and were stimulated with IL12 (10 ng/mL), IL15 (10 ng/mL) and IL18 (50 ng/mL) for 18 h in the presence or absence of macrophages, which were purified from the peritoneal cavity. Macrophages were left unstimulated or stimulated with LPS (10 ng/mL). IL2-NK cell (**A**) or splenic NK cell (**F**) cytotoxicity against RMA/S target cells was assessed by fluorescence release cytotoxicity assay. Data are mean ± SEM of four biological replicates across two independent experiments. Statistical analysis was performed using a two-way ANOVA. **B**, **G** Intracellular IFNγ production was measured by flow cytometry. Pooled data are mean ± SEM of 11 biological replicates (**B**) or 14 biological replicates (**G**) across four independent experiments (**B**) or five independent experiments (**G**). **C** Purified IL2-NK cells were analysed by RT-qPCR for expression of Ifng mRNA and normalised to control genes Gapdh and Rplp0. Data are the mean of at least seven biological replicates across four independent experiments. **D**, **H** IFNγ secretion in co-culture medium was measured by cytokine bead array. Data are mean ± SEM of four biological replicates across two independent experiments. **E**, **I** T-bet expression was measured by flow cytometry. Pooled data ± SEM of six biological replicates across two independent experiments. Statistical analysis was performed using a two-way ANOVA (**A**, **F**), a one-way ANOVA with Tukey’s post-test (**B**, **E**, **G**–**I**), or a two-way ANOVA of Tukey’s post-test (**C**, **D**).

To investigate how macrophage–NK cell interactions might contribute to the metabolic and functional phenotype of pNK cells in the inflamed peritoneum, co-cultures were performed using bone marrow-derived macrophages (BMDMs) and IL2-expanded NK (IL2-NK) cells stimulated with IL2 and IL12. To address concerns that cultured immune cells have different metabolic features than those isolated from mice before analysis, co-cultures were also performed with peritoneal macrophages and splenic NK cells stimulated with IL12, IL15 and IL18, a potent stimulus for spNK cells^19^. Firstly, NK cell effector functions were characterised in these co-cultures. In both co-cultures, the presence of macrophages, with and without LPS stimulation, did not impact in vitro cytotoxicity of NK cells against RMA/S target cells (Fig. 3A, F).

In co-cultures of IL2-NK cells and LPS-stimulated BMDMs, perforin expression was unchanged (Supplementary Fig. 3B), and although granzyme B levels were significantly reduced, NK cells still expressed approximately twice as much granzyme B protein as unstimulated NK cells (Supplementary Fig. 3C). In co-cultures of splenic NK cells and peritoneal macrophages, there was a significantly reduction expression of perforin (Supplementary Fig. 3F), yet levels remained >2-fold higher than in unstimulated NK cells, and granzyme B expression was unchanged (Supplementary Fig. 3G). In both co-cultures, the presence of LPS-stimulated macrophages significantly reduced NK cell IFNγ production; % IFNγ NK cells were reduced by 26% and 44% in IL2-NK cells and spNK cells, respectively (Fig. 3B, G). Unstimulated peritoneal macrophages, but not unstimulated BMDM, also had a significant but less robust impact on IFNγ production in spNK cells, which showed a 23% reduction in IFNγ+ spNK cells (Fig. 3G).

Assessment of Ifng mRNA expression in IL‑2-expanded NK cells co‑cultured with BMDMs revealed a significant increase at 8 h compared with IL‑2-NK cells cultured alone. By 24 h, however, Ifng transcript levels were markedly reduced (Fig. 3C). A similar pattern was observed at the protein level. IL‑2-NK cells cultured alone continued to increase IFN‑γ secretion over the 24 h period, whereas NK cells co‑cultured with LPS‑stimulated BMDMs showed an early rise in IFNγ secretion at 4 h, followed by a pronounced suppression at 24 h (Fig. 3D). Comparable results were obtained in co‑cultures of splenic NK cells with peritoneal macrophages, where significantly less IFNγ was present in the supernatant after 24 h in the presence of macrophages (Fig. 3H). To investigate whether altered transcriptional regulation contributed to this suppression, we examined expression of T‑bet, a key transcription factor for IFNγ production. In IL‑2-NK cells co‑cultured with BMDMs, T‑bet expression was unchanged (Fig. 3E). In splenic NK cells co‑cultured with peritoneal macrophages, T‑bet expression was modestly reduced, but all NK cells remained T-bet positive with levels higher than unstimulated NK cells (Fig. 3I). We also assessed Eomes, another transcription factor that regulates IFNγ and is a discriminator between NK cells and ILC1 cells. In both co‑culture systems, Eomes expression was unchanged (Supplementary Fig. 3A, E).

### Macrophage–NK cell interactions induce LD accumulation in NK cells

**Fig. 4:**
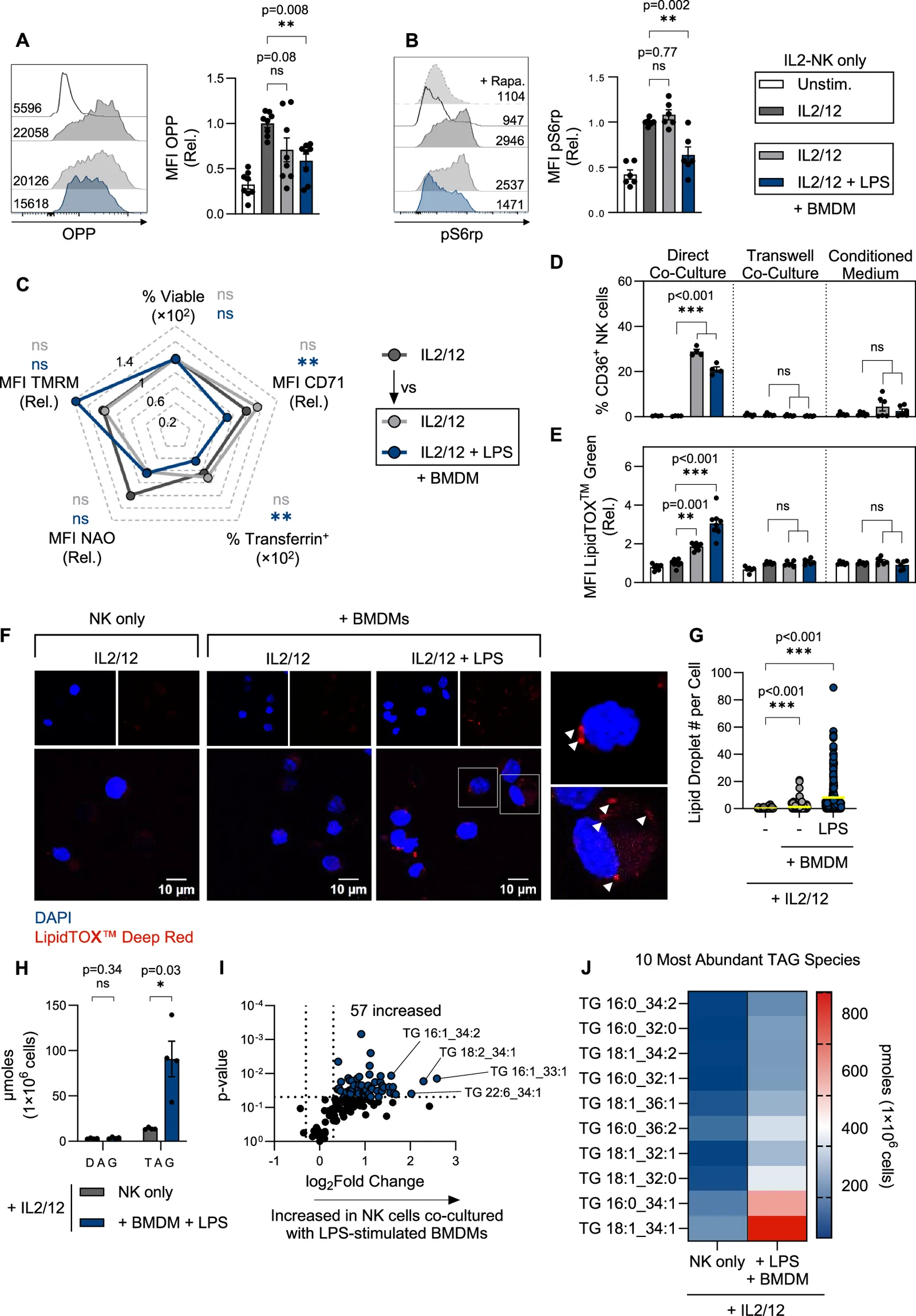
Direct interactions between macrophages and NK cells affect NK cell metabolism and induce LD accumulation. **A**–**C**, **F**–**J** BMDMs were stimulated with LPS (10 ng/mL) or left unstimulated for 6 h. Then, IL2-NK cells were directly co-cultured with BMDMs for 18 h in the presence of NK cell-activating cytokines IL2 (20 ng/mL) and IL12 (10 ng/mL). **D**, **E** BMDMs were stimulated with LPS (10 ng/mL) or left unstimulated for 6 h. Then, IL2-NK cells were either directly co-cultured with BMDMs or co-cultured in a trans-well for 18 h in the presence of NK cell-activating cytokines IL2 (20 ng/mL) and IL12 (10 ng/mL). Alternatively, NK cells were stimulated with IL2 (20 ng/mL) and IL12 (10 ng/mL) for 18 h in 70% conditioned medium collected from BMDMs previously stimulated with LPS (10 ng/mL) or unstimulated for 24 h. **A** O-propargyl puromycin (OPP) (20 µM) incorporation into proteins was measured by flow cytometry. Data are mean ± SEM of 11 biological replicates across four independent experiments. **B** pS6 levels in NK cells compared to rapamycin mTORC1 negative control. **C** NK cell viability, CD71 expression, transferrin-AF647 (5 µg/mL), nonylacridine orange (NAO) (100 nM) and TMRM (100 nM) were measured by flow cytometry. Data are the mean of at least four biological replicates across at least two independent experiments. **D** CD36 expression was measured by flow cytometry. Data are the mean ± SEM of four biological replicates across two independent experiments for direct co-culture, or six biological replicates across two independent experiments for trans-well and conditioned medium. **E** LipidTOX™ Green staining was measured by flow cytometry. Data are mean ± SEM of eight biological replicates across three independent experiments for direct co-culture, or six biological replicates across two independent experiments for trans-well and conditioned medium. **F**, **G** LipidTOX™ Deep Red and DAPI staining was carried out on fixed cells at 4 °C for 1 h, prior to measurement by confocal microscopy. Data are the mean of at least 172 cells from three biological replicates across three independent experiments. **H**–**J** Purified NK cells were analysed by FIA/MS for quantification of diacylglycerols (DAG) and triacylglycerols (TAG). Data are quantification of total DAGs and TAGs (**H**), a volcano plot showing the number of significantly increased DAG and TAG species (**I**) or quantification of the top 10 most abundant TAG species present (**J**). Statistical analysis was performed using a one-way ANOVA with Tukey’s post-test (**A**–**E**), a Kruskal–Wallis test with Dunn’s post-test (**G**) or unpaired two-tailed t-tests (**H**, **I**).

The impact of macrophages on NK cell metabolism was also assessed in vitro, using co-culture approaches. The data showed a significant decrease in rates of protein translation in NK cells cultured with LPS-stimulated BMDMs, suggestive of reduced ATP production, but there was no change in mitochondrial parameters (mitochondrial mass and membrane potential) or NK viability (Fig. 4A, C and Supplementary Fig. 4A–C). There was a reduction in mTORC1 activity in IL2-NK cells co-cultured with LPS-stimulated BMDMs, measured through phosphorylation of S6 ribosomal protein (pS6rp), with rapamycin (30 min) treated NK cells from the same co-culture used to visualise an mTORC1 'off' signal (Fig. 4B). Given the notable increase in CD71 expression and transferrin uptake in pNK cells in LPS-induced peritonitis, these parameters were also measured in IL2-NK-BMDM co-cultures. In the presence of LPS-stimulated BMDMs, there was a significant decrease in CD71 expression, which was mirrored by decreased transferrin uptake in IL2-NK cells (Fig. 4C and Supplementary Fig. 4D, E). Measurement of total intracellular iron in IL2-NK cells showed no difference in total iron content when NK cells were co-cultured with BMDMs (Supplementary Fig. 4F). As pNK cells showed increased lipid accumulation observed in the LPS-induced peritonitis model, the abundance of neutral lipids were quantified in co-cultured NK cells. In the presence of BMDMs, IL2-NK cells showed a significant increase in expression of CD36, which was not observed in trans-well co-cultured NK cells or those exposed to BMDM conditioned medium (Fig. 4D). Similarly, direct cell–cell interactions with BMDMs were required for increased neutral lipids abundance in IL2-NK cells, with co-cultured LPS-stimulated BMDMs inducing the highest lipid levels (Fig. 4D). Similar increases in CD36 expression and neutral lipid accumulation were shown using co-cultures of splenic NK cells and peritoneal macrophages in the absence of cytokine stimulation, though the magnitude of the effect was smaller (Supplementary Fig. 4H, I). mTORC1 signalling was measured in spNK cells co-cultured with peritoneal macrophages; significant decreases in pS6rp levels were measured in spNK cells stimulated with IL2/15/18 while co-cultured with LPS-stimulated peritoneal macrophages (Supplementary Fig. 4J–L). Unstimulated peritoneal macrophages also inhibited mTORC1 activity in co-cultures spNK cells but to a lesser degree (Supplementary Fig. J–L). Next, the distribution of neutral lipids in IL2-NK cells was analysed using confocal microscopy; the data revealed that neutral lipids are primarily stored in LDs, evident as LipidTOX^TM^ Deep Red staining foci (Fig. 4F, G). To quantify and characterise the neutral lipids that accumulate in NK cells as LDs, lipid metabolomics was performed for IL2-NK cells alone or following co-culture with LPS-stimulated BMDMs. These data revealed a significant increase in the abundance of intracellular TAGs, but not DAGs, in NK cells co-cultured with LPS-stimulated BMDMs (Fig. 4H and Supplementary Data 1). A total of 57 significantly increased neutral lipid species were detected within NK cells following co-culture with LPS-stimulated BMDMs, all of which were TAGs (Fig. 4I). Ranking of the top ten most abundant TAG species in NK cells following co-culture with LPS-stimulated BMDMs revealed that they were all esterified with palmitic acid (16:0) and oleic acid (18:1) (Fig. 4J).

### NK cells rapidly trogocytose CD36 protein from macrophages

**Fig. 5:**
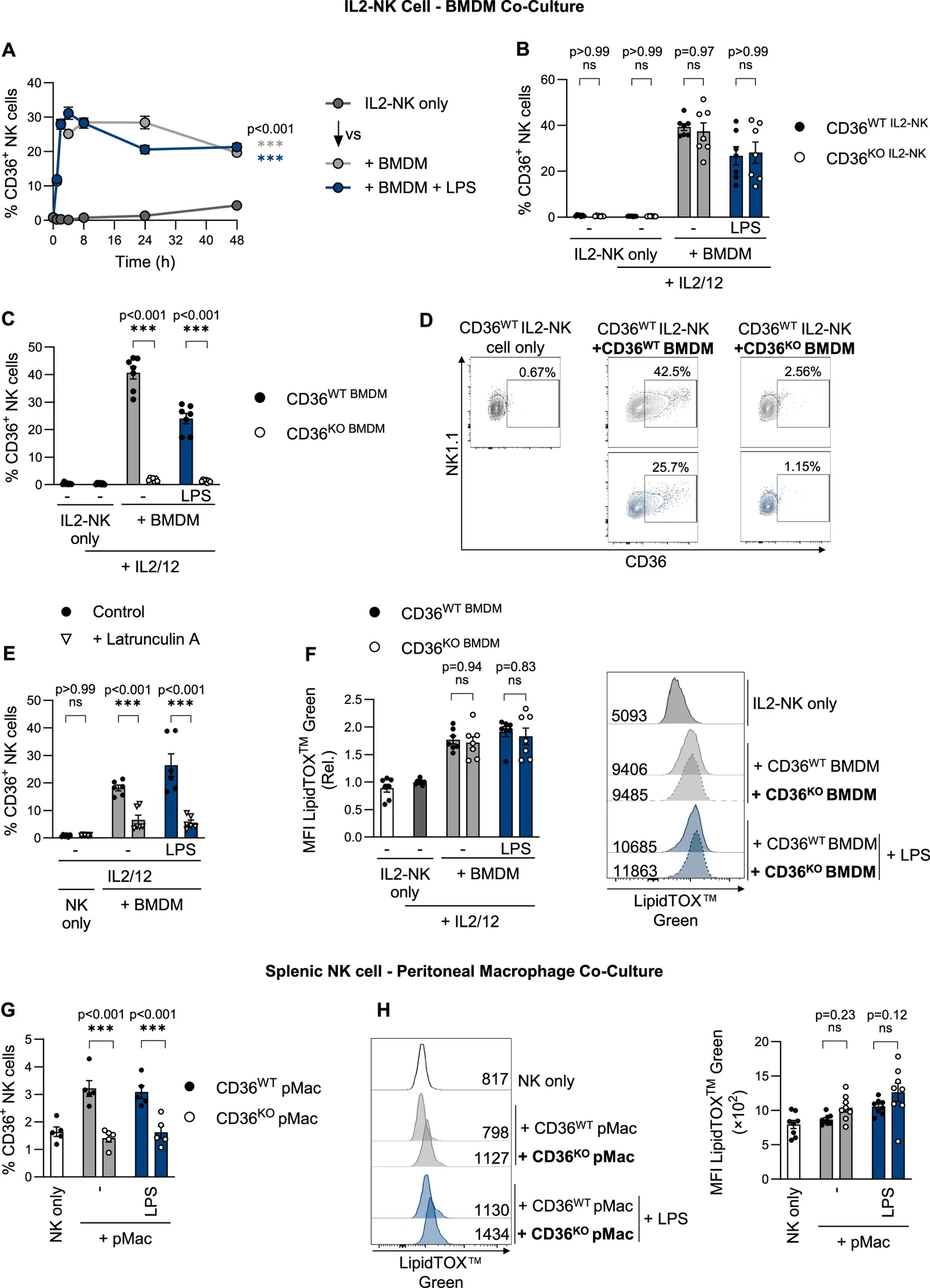
NK cells trogocytose CD36 following direct interactions with macrophages. **A** BMDMs were stimulated with LPS (10 ng/mL) or left unstimulated for 6 h. Then IL2-NK cells were directly co-cultured with BMDMs for the length of time stated on the graph in the presence of NK cell-activating cytokines IL2 (20 ng/mL) and IL12 (10 ng/mL). Data are the mean ± SEM of at least three biological replicates across two independent experiments. **B** CD36WT BMDMs were stimulated with LPS (10 ng/mL) or left unstimulated for 6 h. Then either CD36WT or CD36KO IL2-NK cells were directly co-cultured with BMDMs for 18 h in the presence of NK cell-activating cytokines IL2 (20 ng/mL) and IL12 (10 ng/mL). Data are mean ± SEM of seven biological replicates across two independent experiments. **C**, **D**, **F** CD36WT and CD36KO BMDMs were stimulated with LPS (10 ng/mL) or left unstimulated for 6 h. Then CD36WT IL2-NK cells were directly co-cultured with BMDMs for 18 h in the presence of NK cell-activating cytokines IL2 (20 ng/mL) and IL12 (10 ng/mL). Data are mean ± SEM of seven biological replicates across two independent experiments. **E** BMDMs were stimulated with LPS (10 ng/mL) or left unstimulated for 6 h. Then IL2-NK cells were directly co-cultured with BMDMs for 8 h in the presence of NK cell-activating cytokines IL2 (20 ng/mL) and IL12 (10 ng/mL). Co-cultures were carried out in the presence or absence of latrunculin A (10 µM). Data are mean ± SEM of six biological replicates across two independent experiments. **G**, **H** CD36WT NK cells were MACS purified from the spleen and were cultured for 18 h in the presence or absence of CD36WT or CD36KO macrophages, which were purified from the peritoneal cavity. Macrophages were left unstimulated or stimulated with LPS (10 ng/mL). Data are mean ± SEM of five biological replicates per group across two independent experiments (**G**) or eight biological replicates across three independent experiments (**H**). **A**–**E**, **G** CD36 expression was measured by flow cytometry. **F**, **H** LipidTOX™ Green staining was measured by flow cytometry. Statistical analysis was performed using a two-way ANOVA with Tukey’s post-test (**A**) or Šidák’s post-test (**B**, **C**, **E**–**H**).

The increase in CD36 expression seen in NK cells co-cultured with macrophages, is not seen in NK cells activated by cytokines in isolation, suggesting macrophages induce CD36 expression in NK cells. A time course of CD36 expression by IL2-NK cells revealed that the expression of CD36 on NK cells occurs rapidly, starting as early as 1 h after the start of the co-culture with BMDMs and reaching a plateau at 2 h (Fig. 5A). To investigate the importance of this rapid NK cell CD36 expression for LD accumulation in NK cells, CD36^KO^ mice (C57Bl/6 background, whole body deletion, Supplementary Fig. 5A) were challenged with LPS and PEC cells isolated before analysis of neutral lipid content. The data show that pNK cells have equivalent neutral lipid content in LPS-challenged CD36^KO^ and CD36^WT^ mice (Supplementary Fig. 5B). However, a caveat of this experiment is that the macrophages also lack CD36 expression, and it is not known how this impacts the system. Therefore, to confirm that CD36 expression is not essential for LD accumulation in NK cells, co-culture experiments were performed using CD36^WT^ BMDM and CD36^KO^ IL2-NK cells. The splenic cells used to generate IL2-NK cells were confirmed to be CD36-null (Supplementary Fig. 5A). Nevertheless, the co-cultured CD36^KO^ IL2-NK cells had equivalent levels of CD36 protein to CD36^WT^ IL2-NK controls (Fig. 5B). This unexpected result suggested that the CD36 protein was expressed in the CD36^WT^ BMDM cells and transferred to CD36^KO^ IL2-NK cells. To test this, the reverse experiment was performed using CD36^KO^ BMDM and CD36^WT^ IL2-NK cells. In this co-culture experiment, the CD36^WT^ IL2-NK cells did not increase CD36 expression and remained CD36 negative, thus providing evidence that NK cells are obtain CD36 protein from macrophages (Fig. 5C, D). This aligns with data showing that the increase in CD36 protein expression actually occurs before a significant increase in *Cd36* mRNA expression (Fig. 5A and Supplementary Fig. 5C). Together, these data argue IL2-NK cells acquire CD36 protein from BMDMs through trogocytosis, an active and actin-dependent process previously described to allow NK cells to take plasma membrane proteins, including as PD1 and MHCII, from other cells^13,22^. To confirm that the acquisition of CD36 by NK cells was an active process, co-cultures performed at 4 °C, which restricted the generation of CD36^+^ NK cells (Supplementary Fig. 5D). Next, a more direct approach was used to target trogocytosis; IL2-NK cells were co-cultured with BMDMs in the presence an inhibitor of actin polymerisation, latrunculin A, which significantly reduced CD36 expression by the IL2-NK cells by 75–80% (Fig. 5E). To support these results, similar experiments were performed with spNK cells co-cultured with CD36^KO^ peritoneal macrophages. No increase in CD36-positive spNK cells was observed when cultured with CD36^KO^ macrophages (Fig. 5G). In the LPS-induced peritonitis model, there was no difference in lipid content in CD36^KO^ mice (Supplementary Fig. 5B), arguing that CD36 is not required for lipid uptake and storage in pNK cells. In co-cultured NK cells, the data showed that the trogocytosis of CD36 from macrophages was not required for lipid accumulation in NK cells; the neutral lipid content of IL2-NK cells or spNK cells were equivalent when co-cultured CD36^WT^ and CD36^KO^ BMDM and peritoneal macrophages, respectively (Fig. 5F, H). Taken together, these data argue that the lipid accumulation in NK cells is occurs through CD36-independent mechanisms.

### De novo synthesised FA from macrophages accumulate in co-cultured NK cells

**Fig. 6:**
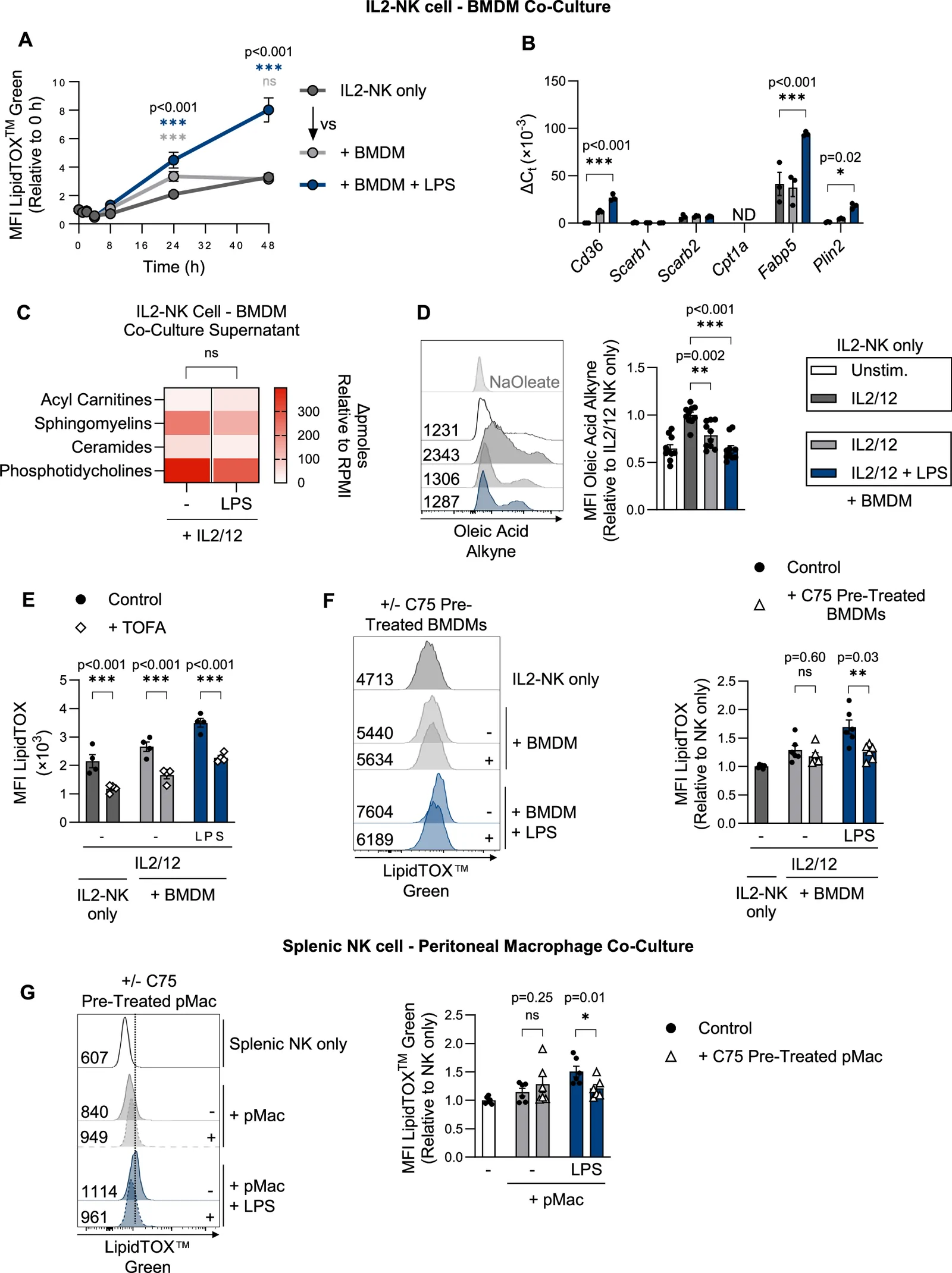
Macrophage-derived de novo synthesised FA are required for lipid accumulation in co-cultured NK cells. **A**, **B**, **D** BMDMs were stimulated with LPS (10 ng/mL) or left unstimulated for 6 h. Then IL2-NK cells were co-cultured with BMDMs for the length of time stated on the graph (**A**) or 18 h (**B**, **D**) in the presence of IL2 (20 ng/mL) and IL12 (10 ng/mL). **A** Data are mean ± SEM of at least three biological replicates across two independent experiments. **B** Purified IL2-NK cells were analysed by RT-qPCR for expression of Cd36, Scarb1, Scarb2, Cpt1a, Fabp5 and Plin2 mRNA and normalised to control genes Gapdh and Rplp0. Data are mean ± SEM of three biological replicates across two independent experiments. **C** BMDMs were stimulated with LPS (10 ng/mL) or left unstimulated for 24 h. IL2-NK cells were activated for 24 h with IL2 (20 ng/mL) and IL12 (10 ng/mL). NK cells were then co-cultured with BMDMs for a further 24 h before lipids in the cell culture medium were measured by FIA/MS. Data are the mean of three biological replicates. **D** Oleic acid alkyne (6 µM) uptake by NK cells was measured by flow cytometry. Sodium oleate (NaOleate) (60 µM) was used as a competition control. Data are mean ± SEM of 10 biological replicates across three independent experiments. **E** BMDMs were stimulated with LPS (10 ng/mL) or left unstimulated for 6 h. Then IL2-NK cells were co-cultured with BMDMs for 18 h in the presence of IL2 (20 ng/mL) and IL12 (10 ng/mL) in the presence or absence of TOFA (15 µM). Data are mean ± SEM of four biological replicates. **F** BMDMs were stimulated with LPS (10 ng/mL) or left unstimulated for 6 h in the presence or absence of C75 (40 µM). C75 was washed off before IL2-NK cells were co-cultured with BMDMs for 18 h in the presence of IL2 (20 ng/mL) and IL12 (10 ng/mL). Data are the mean ± SEM of six biological replicates across three independent experiments. **G** Macrophages were purified from the peritoneal cavity and pre-treated for 2 h with C75 (40 µM). C75 was washed off before macrophages were left unstimulated or stimulated with LPS (10 ng/mL). Then NK cells were MACS purified from the spleen and cultured for 18 h in the presence or absence of macrophages. Data are mean ± SEM of four biological replicates across two independent experiments. Statistical analysis was performed using a two-way ANOVA with Tukey’s post-test (**A**) or Šidák’s post-test (**E**–**G**), unpaired two-tailed t-test (**C**) or a one-way ANOVA with Tukey’s post-test (**B**, **D**).

A time course experiment revealed that lipid accumulation by IL2-NK cells in the presence of LPS stimulated BMDMs was significantly increased at after 24 h of the co-culture (Fig. 6A). However, there was a trend towards increase lipids after just 8 h, which was confirmed to be significant with addition replicates focusing on this time point (Supplementary Fig. 6A). The expression of lipid transporters and lipid-binding proteins at 24 h was increased, including the expression of *Fabp5* and *Plin2*, proteins important for storage of intracellular FA (Fig. 6B). While Fig. 4D shows that direct co-culture is required for lipid accumulation, this does not necessarily mean that lipids are transferred directly to physically interacting NK cells. Another explanation could be that contact-dependent signals from macrophages induce lipid handling proteins in NK cells, including PLIN2 and FABP5 (Fig. 6B), allowing NK cells to uptake and store FA from the medium. Whether FA are secreted from macrophages for the purpose of uptake into NK cells was also not known. To investigate these alternative mechanisms, targeted lipid metabolomics was performed on the culture medium from BMDM IL2-NK cells cocultures, comparing cocultures with unstimulated and LPS-stimulated BMDM. The data showed that the increases in lipid species in either co-culture condition only occurred in the picomolar range (Fig. 6C). Were BMDM stimulating IL2-NK cells to take up exogenous lipids in the provided media, one might expect to see a depletion of lipid species in the LPS-stimulated co-culture; however, there was no change in the abundance of these lipid species between both these co-culture conditions (Fig. 6C and Supplementary Data 2). Alternatively, were macrophages releasing lipids into the media to be taken up and stored by IL2-NK cells, an accumulation of lipid species in the media might be predicted. However, balance secretion of FA from macrophages and uptake by NK cells might be consistent with these metabolomic data. However, arguing against this scenario is measurements of the FA uptake capacity of IL2-NK cells following cocultured with LPS-stimulated BMDM. Here the data showed a significantly reduced rate of fatty acid uptake in IL2-NK cells, measured with alkyne-oleic acid (Fig. 6D). Considering oleic acid was the most abundant esterified fatty acid in intracellular TAGs in NK cells (Fig. 4I), the decrease in the IL2-NK cells ability to take up oleic acid alkyne argues against a contribution for uptake of extracellular FA to the generation of LDs within NK cells. Neutral lipid accumulation in co-cultured NK cells is an active process and is blocked then the co-culture is performed at 4 °C (Supplementary Fig. 6B). Next, we tested if de novo synthesis of FA or cholesterol esters is required. Co-cultures were performed in the presence of 5-(tetradecyloxy)-2-furoic acid (TOFA), a pharmacological inhibitor of acetyl-CoA carboxylase (ACC) 1, the first enzyme in the de novo FA synthesis pathway, and zaragozic acid, an inhibitor of squalene synthase, the first committed step of cholesterol synthesis. The data show that TOFA treatment, but not zaragozic acid, significantly reduced lipid accumulation in NK cells in all conditions (Fig. 6E and Supplementary Fig. 6C). These data show that de novo synthesis of FA is required for the formation of LDs in NK cells, whereas cholesterol synthesis, for the generation of cholesterol esters, is dispensable. However, this experiment did not discriminate between fatty acid synthesis in macrophages or NK cells. To identify which cell type makes the FA that accumulate in the NK cells, an irreversible inhibitor of fatty acid synthase (FASN) called 4-methylene-2-octyl-5-oxotetrahydrofuran-3-carboxylic acid (C75) was used^23^. BMDMs were pre-treated with C75, and the cells were subsequently extensively washed before co-culture with NK cells; in these co-cultures, macrophage FASN is inactivated while NK cell FASN is not pharmacologically impaired. BMDM tolerate treatment with C75; cell viability was not compromised (data not shown and ref. ^24^). Both experiment formats, IL2-NK cells plus BMDM and spNK plus peritoneal macrophages, gave the same result. Pre-treatment of macrophages with C75 significantly reduced the abundance of neutral lipids in NK cells that were co-cultured with LPS-stimulated macrophages, bringing neutral lipid staining down to levels comparable to NK cells co-cultured with unstimulated macrophages (Fig. 6F, G). C75 pretreatment had no impact on the lipid levels in NK cells cultured with unstimulated macrophages. Taken together, these experiments demonstrate that FA generated within inflammatory macrophages, by de novo *FA* synthesis, then accumulate within 8 h in physically interacting NK cells.

### FA from macrophage suppress NK cell IFNγ production

IFNγ expression in NK cells has consistently been associated with activated mTORC1 signalling^18,25,26^. Data in Fig. 4B showed decreased phosphorylation of S6rp in NK cells, indicating reduced mTORC1 activity. mTORC1 was originally characterised as an amino‑acid sensing protein kinase, but in this scenario mTORC1 inhibition is not associated with amino acid limitations. The amino‑acid transporter SLC7A5 is crucial for transporting amino acids to support mTORC1 activity in NK cells^27^. However, there was no decrease in SLC7A5 activity, measured using a kynurenine-based uptake assay^28^, in lipid loaded IL2-NK cells (Supplementary Fig. 7A). We also measured the abundance of extracellular amino acids required to sustain mTORC1 signalling and confirmed that these metabolites remained readily available in the culture supernatant (Supplementary Fig. 7B). These data support an alternative mechanism for the inhibition of mTORC1 in these NK cells.

**Fig. 7:**
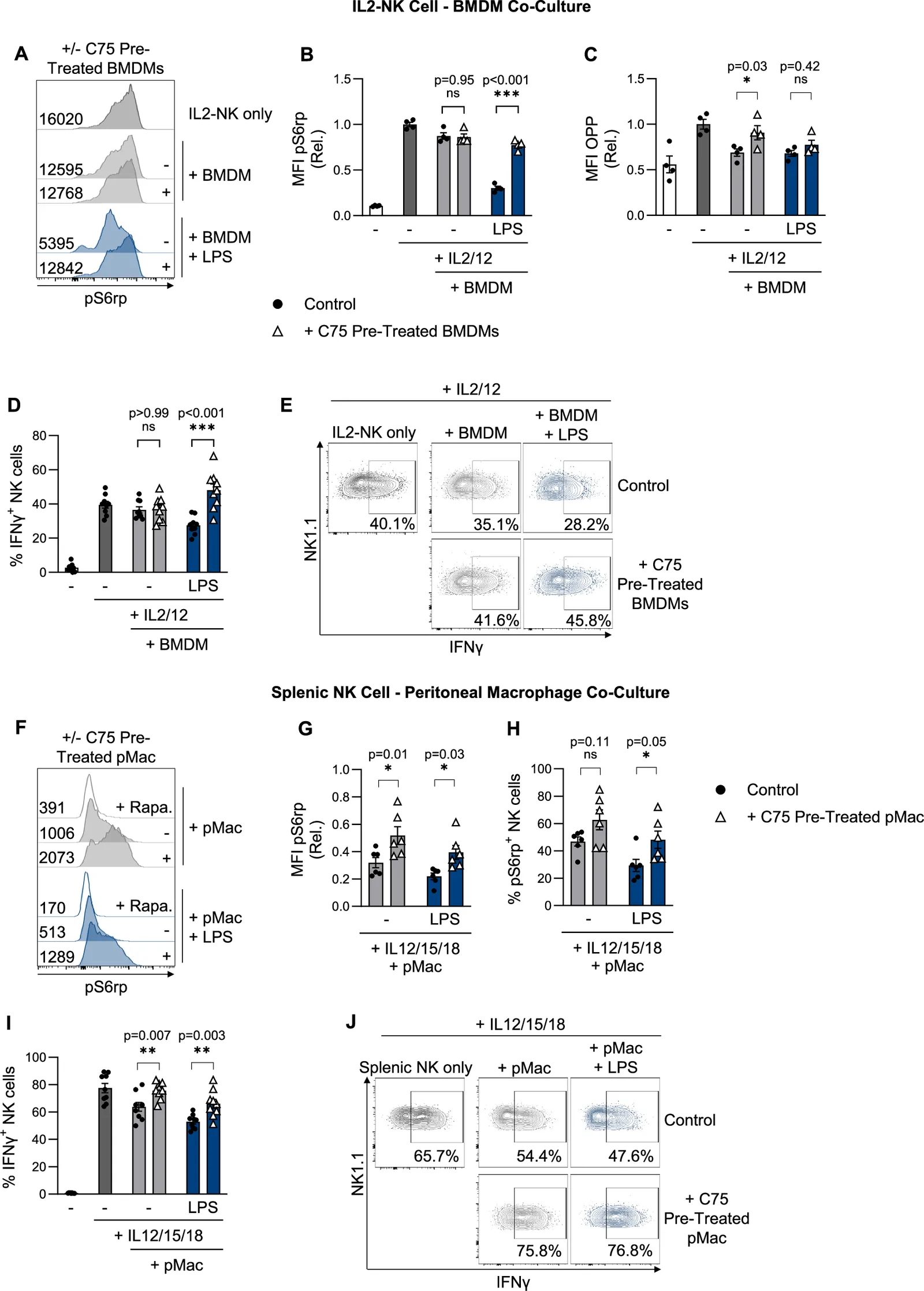
Macrophage-derived de novo synthesised FA suppress NK cell activation and IFNγ production. **A**–**E** BMDMs were stimulated with LPS (10 ng/mL) or left unstimulated for 6 h in the presence or absence of C75 (40 µM). C75 was washed off before IL2-NK cells were co-cultured with BMDMs for 18 h in the presence of IL2 (20 ng/mL) and IL12 (10 ng/mL). **F**–**J** Macrophages were purified from the peritoneal cavity and pre-treated for 2 h with C75 (40 µM). C75 was washed off before macrophages were left unstimulated or stimulated with LPS (10 ng/mL). Then NK cells were MACS purified from the spleen and were stimulated with IL12 (10 ng/mL), IL15 (10 ng/mL) and IL18 (50 ng/mL) for 18 h in the presence or absence of macrophages. **A**, **B**, **F**–**H** Phosphorylation of S6rp was measured by flow cytometry. Pooled data are the mean ± SEM of four biological replicates across two independent experiments (**B**) or six biological replicates across three independent experiments (**G**, **H**). **C** O-propargyl puromycin (OPP) (20 µM) incorporation into proteins was measured by flow cytometry. Data are mean ± SEM of four biological replicates across two independent experiments. **D**, **E**, **I**, **J** IFNγ expression was measured by flow cytometry. Pooled data are mean ± SEM of nine biological replicates across three independent experiments (**D**) or nine biological replicates across four independent experiments (**I**). Statistical analysis was performed using a two-way ANOVA and Šidák’s post-test (**B**–**D**, **G**–**I**).

Experiments were performed using C75 to investigate if FA generated in the macrophage can affect mTORC1 activity in interacting NK cells. Previous studies have shown in BMDM that C75 treatment affects LPS signalling and cytokine outputs, including TNFα and IL6. However, in these experiments we have added IL2 and IL12 cytokines to ensure full cytokine stimulation signals are received by NK cells. The experimental result was clear; pretreatment of LPS‑stimulated BMDMs with C75 restored mTORC1 activity in interacting IL2-NK cells (Fig. 7A). However, the metabolic rescue was not complete because protein translation rates did not increase in IL2-NK cells when macrophages were pretreated with C75 (Fig. 7C). Nevertheless, despite reduced translation rates, the increased mTORC1 activity was accompanied by the marked recovery of IFN‑γ production (Fig. 7D, E). These findings demonstrate that blocking the ability of BMDMs to synthesise and transfer FA to IL2-NK cells reverses the macrophage‑dependent inhibition of mTORC1 activity and IFN‑γ production.

Further evidence was generated using C75-pre-treated peritoneal macrophages co-cultured with spNK cells. As the literature does not describe the impact of C75 on peritoneal macrophages, we first investigated peritoneal macrophages in isolation. In the presence of C75, peritoneal macrophages had reduced production of proinflammatory IL12, IL1β, IL6 and TNFα (Supplementary Fig. 7D), similar to cytokine measurements for C75-treated BMDM^24^. Peritoneal macrophages showed no change in the production of the anti-inflammatory cytokines IL10 and TGFβ (Supplementary Fig. 7D). In addition, C75-treated peritoneal macrophages exhibited reduced CD40 expression, a co‑stimulatory ligand for CD40L on NK cells (Fig. 7E)^29^. Taken together, these results show that FASN activity is required for the full capacity of peritoneal macrophages to activate local NK cells. However, in our co‑culture system, the addition of exogenous IL‑12, IL‑15 and IL‑18 ensures robust NK‑cell activation irrespective of macrophage‑derived cytokine support. This allows us to isolate and evaluate any suppressive effects arising specifically from FA transfer from peritoneal macrophages to NK cells. In this experiment, co‑culture of spNK cells with peritoneal macrophages resulted in reduced mTORC1 and IFNγ production, and this was exacerbated with LPS-stimulated peritoneal macrophages, as observed previously (Supplementary Fig. 4J–L). Importantly, for peritoneal macrophages treated with or without LPS, pretreatment with C75 significantly increased mTORC1 activity within interacting spNK cells, measured as increased MFI of pS6rp and increased in pS6rp+ NK cells (Fig. 7F–H). Importantly, increased mTORC1 signalling was accompanied by a robust rescue of IFN‑γ production (Fig. 7I, J). Taken together, these data support the conclusion that macrophage‑derived FA synthesis is a key suppressive event that limits NK cell IFN‑γ production.

Overall, this study has demonstrated that physical interactions of NK cells with inflammatory macrophages result in a previously unappreciated level of metabolic crosstalk, wherein FA synthesised in macrophages are transferred directly to NK cells, leading to an accumulation of LDs and a decrease in the IFNγ output. This represents an additional mechanism for inflammatory systems to sustain balanced responses, a metabolic-mediated negative feedback loop to limit excess inflammation and damage.

## Discussion

Fatty acid (FA) synthesis, transport and storage are central to cellular energetics, membrane biogenesis and signalling, and their immunoregulatory roles are now recognised across innate and adaptive immune cells. Prior studies have established that lipid accumulation and altered FA handling can profoundly change NK cell phenotype and function, particularly in pathophysiological settings such as obesity and cancer. For example, obesity-associated lipid exposure drives lipid accumulation in NK cells and impairs mTORC1-dependent glycolysis, trafficking and cytotoxicity^16^. Impaired NK cell metabolism and function in humans with obesity can be revived by metabolic interventions such as GLP-1 therapy; peripheral NK from treated patients showed improved mTORC1 activity and IFNγ production^30^. A number of studies have directly studied mTORC1 signalling in NK cells and described reduced IFNγ production when mTORC1 activity was inhibited^18,25,26^. These data, together with studies of NK dysfunction in lipid-rich tumours and other metabolic microenvironments, have led to the view that FA accumulation in NK cells is largely deleterious^31,32^.

At the same time, other recent work emphasizes that NK cells can actively utilise FA and FA-oxidation (FAO) during immune challenge: NK cells increase FA uptake and CD36 expression after acute viral infection, and CPT1A-dependent FAO supports NK responses in infection and tumour models^20,21^. Thus, FA biology in NK cells is context-dependent and can support or constrain effector responses depending on the stimulus, duration and local milieu.

Our data refine this emerging model, supporting an additional and more subtle layer of NK cell regulation by lipids. The data argue that macrophages can actively deliver newly synthesised FA to NK cells through direct contact, thereby reshaping NK cell metabolism and cytokine production. When stimulated with LPS, macrophages triggered lipid‑droplet accumulation in NK cells composed of predominantly palmitic and oleic acid-containing TAGs and induced the expression of fatty‑acid-handling genes. Pharmacological inhibition of FASN specifically in macrophages prevented lipid accumulation in NK cells, linking de novo synthesis of FA in macrophages FA to LD-mediated regulation of NK cells. The hallmark products of de novo fatty‑acid synthesis are PA and then OA, after SCD1‑dependent desaturation of PA. These FA species match exactly with FA that overwhelmingly dominate the FA chains in the TAGs that accumulate in NK cells. The data show that LD affect multiple aspects of NK cell biology including metabolism, signal transduction and cytokine output; LD fuel OXPHOS while concurrently inhibiting mTORC1 signalling and reducing IFN‑γ production. Interestingly, the accumulation of LD does not account for every aspect of macrophage regulation of NK cell metabolism, as interventions that prevented LD accumulation in NK cells did not reverse decreased rates of protein translation.

Our findings position mTORC1 as a key node linking macrophage-derived lipid transfer to suppression of NK cell function. While mTORC1 inhibition is typically associated with nutrient limitation, our data support a model in which lipid accumulation contributes directly to mTORC1 attenuation. Importantly, inhibition of fatty acid synthesis in macrophages restored both mTORC1 activity and IFN-γ production, demonstrating functional reversibility through upstream metabolic intervention. The mechanisms underlying this effect remain to be defined but are likely multifactorial. Uptake of membrane material via contact-dependent transfer may increase endo-lysosomal flux, potentially perturbing lysosomal function; lysosomal stress is suggested as a mechanism for the dislocation of mTORc1 from the lysosome, hence mTORc1 inhibition^33,34^. In parallel, rapid lipid droplet formation is consistent with ER lipid buffering and stress responses, which can suppress mTORC1^35–37^.

The rapid appearance of the scavenger receptor CD36 on NK cells once they interact with macrophages was striking. Notably, CD36 did not arise from NK‑cell transcriptional upregulation but was instead acquired directly from macrophages via trogocytosis. Trogocytosis, the transfer of membrane fragments and associated proteins between immune cells, has long been recognised across T, B and NK cells and is known to alter NK‑cell phenotype and function^38–40^. For example, NK cells can acquire inhibitory receptors that suppress anti‑tumour activity; CAR‑NK cells can capture tumour antigens with functional consequences; and trogocytosis of MHC class II from dendritic cells enables NK‑mediated inhibition of CD4 T cells^13,22,41^. Our findings therefore identify CD36 as part of the repertoire of proteins that NK cells can obtain from neighbouring cells. Although CD36 is a canonical FA scavenger and has been implicated in FA uptake and immune dysfunction in several contexts^42^, our genetic knockout experiments show that bulk lipid accumulation in NK cells during co‑culture occurs largely independently of CD36. In fact, the data argue against classical FA uptake as the mechanism of lipid accumulation in NK cells, as lipids did not accumulate lipids in conditioned media and transwell assays, indicating that physical interaction is essential. In fact, NK cells in co-culture with macrophages have a decreased capacity for FA uptake, measured using an accurate biorthogonal chemistry-based uptake assay approach developed in the lab^43^. It was interesting to note that despite the fact that these NK cells were highly enriched with oleic acid-containing TAGs, their capacity to take up exogenously supplied oleic acid was reduced.

IL-2-activated NK cells in these co-cultures continued to accumulate neutral lipids for at least 48 h, nearly doubling their lipid content between 24 and 48 h. Prolonged lipid loading can trigger ferroptosis or lipotoxic stress when free FA exceed storage capacity^44^. Yet NK cells maintained viability and showed substantially reduced transferrin uptake after cytokine stimulation, suggesting a shift to limit iron import, a potential adaptation to reduce iron-driven lipotoxicity. Certainly, genetic and pharmacological studies have shown that suppression of transferrin-receptor-mediated iron import protects against ferroptotic death^45^. Total intracellular iron remained unchanged in co-cultured IL2-NK cells, supporting an iron-restriction strategy to evade ferroptosis. NK cells also upregulated PLIN2, a lipid-droplet coat protein that stabilises TAG/DAG stores, and confocal imaging confirmed LD deposition. These data suggest that NK cells adapt to sustained macrophage-derived lipid influx by restricting iron uptake to mitigate ferroptotic risk and sequestering free FA as PLIN2-stabilised LDs, thereby buffering against lipotoxicity and preserving viability.

The literature describes a process in macrophages known as focal exocytosis, in which newly synthesised or recycled phospholipids are targeted to discrete regions of the plasma membrane^46^. During this process, endosomes are trafficked to membrane sites, such as the phagocytic cup or points of cell-cell contact, where they deliver and concentrate membrane lipids and proteins through endosome exocytosis^47,48^. This targeted delivery provides a clear route to supply newly synthesised FA, after incorporated into new phospholipids in the endosome membrane, directly to the point of contact with NK cells. The metabolomic dataset was very distinctive in that it shows that the TAGs in the LD are predominantly made up of PA and OA FA chains, a strong argument that NK cells are extracting lipids a region of macrophage membrane highly enriched for newly synthesised phospholipids. Were this not the case and NK cells were acquiring phospholipid species from typical of plasma‑membranes, we would expect a broader distribution of FA chain lengths and degrees of saturation reflecting the endogenous macrophage phospholipid landscape.

Mechanistically, lipid transfer via direct contact could occur through multiple non-exclusive routes. One route for transfer into NK cells is via trogocytosis of membrane patches carrying lipids and membrane proteins into NK cell membranes, a process supported by the observed rapid CD36 acquisition we document and by the literature on trogocytosis^22^. A second mechanism is passive or energy-dependent 'flip-flop' of unionised FA across adjacent plasma membranes: biophysical work shows that protonated FA can translocate across bilayers on millisecond timescales^49^, and that recipient cells use fatty acid binding proteins (FABPs) to solubilise and traffic lipids intracellularly. In lymphocytes and tissue-resident T cells, organ-specific FABP isoforms have been shown to coordinate FA uptake and usage^50^, and FABP5 in particular has been implicated in lymphocyte lipid handling and function^51^. We observed increased *Fabp5* expression in NK cells co-cultured with activated macrophages, consistent with an induced intracellular program to accept and manage acquired lipids and to prevent lipotoxicity. Phospholipid trafficking to the ER can involve vesicular, in the case of trogocytosis, and non-vesicular routes, the case of a flip-flop mechanism^52^. In the ER, phopsholipids are may be broken down, releasing FA chains that are then repackaged by esterification into TAGs, and then stored in LD, a process coordinated by perilipins such as PLIN2 that stabilise neutral lipid storage^53^. We find PLIN2 upregulation in NK cells in our co-culture system, consistent with a controlled, storage-oriented response to lipid influx. Recent quantitative mapping of lipid flux has shown that directional, non-vesicular lipid transport, driven by energy-dependent lipid flipping and lipid-transfer protein machinery, is a principal route for rapid movement of lipids between membranes^52^. Importantly, such mechanisms operate on timescales compatible with the LD formation we detect^52^. For instance, plasma membrane phosphatidylcholine starts to accumulate as neutral lipid species, such as TAGs, after 7 h^52^. Thus, while our finding that NK cells acquire CD36 via trogocytosis remains robust, the bulk accumulation of neutral lipids in NK cells are not acquired by traditional FA uptake routes. The increased lipids in co-cultured NK cells can be reconciled with alternative contact-dependent routes, including energy-coupled flip-flop and/or trogocytosis, consistent with the existing literature^54^. Much focus has been placed on protein transfer via trogocytosis, but this process also involves the transfer of energy‑rich lipid metabolites between interacting cells, and this can be considered a transfer of fuel^55^. In agreement with this idea, NK cells that accumulate LDs catabolise FA via FAO to support mitochondrial respiration and energy production.

Macrophage‑derived lipids suppress mTORC1 activity and reduce IFN‑γ production, but there is no accompanying decrease in NK cell cytotoxicity. Previous studies using rapamycin, a direct mTORC1 inhibitor, show that the loss of mTORC1 activity inhibits IFN‑γ production, the expression of perforin and granzyme B, and NK cell cytotoxicity. However, in our system, mTORC1 activity is reduced but not lost in LD-loaded NK cells; pS6rp levels remain considerably higher that rapamycin-negative controls. Correspondingly, macrophage‑derived lipids suppressed IFN‑γ, but did not impair cytotoxicity. While granzyme B and perforin expression were modestly reduced, NK cells still retained substantial levels of both proteins (well above FMO controls), consistent with preserved killing capacity.

Our data provides insight into the temporal dynamics of NK cell-macrophage crosstalk. We observe rapid acquisition of CD36 by NK cells within 1 h of co-culture, showing rapid onset of trogocytosis. Inflammatory macrophages initially enhance NK cell IFN-γ production, followed by suppression coinciding with the emergence of lipid accumulation in NK cells. These findings support a temporal sequence in which early activating signals are progressively overridden by contact-dependent metabolic regulation. While this framework defines the kinetics of the interaction, our system does not resolve the precise duration or frequency of contact required to drive lipid transfer and functional suppression. Addressing this will require high-resolution live-cell imaging and dynamic tracking approaches to quantify interaction times and link these to metabolic and functional outcomes at the single-cell level.

A key result in this study was the finding that inhibiting FASN, in macrophages only, was sufficient to prevent lipid accumulation in NK cells, to restore mTORC1 activity and IFN‑γ output. The data described in this study, taken altogether with our understanding of macrophage and NK cells biology, allow us to propose a plausible sequence of events as follows: (1) focal exocytosis directs newly synthesised phospholipids to the macrophage-NK cell contact site, (2) NK cells perform trogocytosis, capturing CD36‑containing membrane fragments into endosomes, (3) CD36 is returned to the NK cell plasma membrane via the re‑cycling endosomes where it can be detected by flow cytometry^56^, (4) internalised membrane phospholipids from trogocytosis, or contact dependent 'flip-flop', are trafficked to the ER, metabolised and repackaged into TAGs and LDs^52^. This model integrates our experimental findings with established cellular pathways and provides a coherent explanation for how macrophages deliver newly synthesised lipids to NK cells in a CD36‑independent, contact‑dependent manner. Nevertheless, the precise mechanism of lipid transfer from macrophage to NK cells still remains to be elucidated and will be an important avenue for future investigation.

What might be the physiological consequence of macrophage-to-NK lipid transfer? NK-derived IFNγ is a major macrophage activator, promoting microbicidal and pro-inflammatory programmes. Unrestrained IFN-γ can contribute to pathology: IFNγ neutralisation prolongs survival in perforin-deficient mice with fatal hyperinflammation^57^, and IFN-γ has been implicated in LPS-driven damage^58^. Clinical data also link NK cell abundance and activation with early mortality in septic shock^59,60^. In this light, macrophage-dependent lipid transfer that locally down-regulates NK IFNγ could function as a negative feedback mechanism that prevents persistent macrophage hyperactivation and limits immunopathology. The contact dependence we observe suggests the feedback would operate in a spatially restricted fashion, dampening IFN-γ where macrophages are intensely activated while preserving NK functions at other sites.

Our study has limitations that should guide future work. First, while pharmacologic FASN inhibition and genetic CD36-KO macrophages support a macrophage-dependent lipid source and a role for trogocytosis, we have not fully delineated the molecular machinery of lipid translocation, such as the distinct contributions of trogocytosis versus passive flip-flop or vesicular transfer. Second, the net effect of macrophage lipid transfer on other NK functions, including migration and persistence, and its relevance in distinct in vivo contexts such as infection and sterile inflammation will require targeted models.

In summary, our data define a previously unknown metabolic communication axis in which activated macrophages synthesise and deliver FA to neighbouring NK cells through a contact- dependent mechanism such as trogocytosis, driving lipid storage, inhibiting mTORC1 activity and repressing IFN-γ production. This macrophage-to-NK lipid circuit reframes lipids as active paracrine regulators of innate immunity and suggests that targeting macrophage lipid synthesis or the transfer process itself could be exploited therapeutically to relieve lipid-mediated NK suppression in cancer, or conversely to limit NK-driven immunopathology in hyperinflammatory states.

## Methods

### Animals

Male and female C57BL/6JOlaHsd (6–12 weeks, 057, Inotiv) mice were used in this study. CD36^KO^ (B6.129S1-*Cd36*tm1Mfe/J) mice were purchased from Jackson Laboratories. For experiments using wild-type mice, animals were co-housed. For experiments using CD36^KO^ mice, wild-type controls were age- and sex-matched and were housed separately. All mice were maintained in compliance with project license, EU and Health Products Regulatory Authority regulations, with approval of the University of Dublin’s ethical review board. To induce LPS-induced peritonitis, mice were administered LPS from *E*. coli (O111:B4, 19661, Cayman Chemical, 2.4 mg/kg). Mice were weighed and administered LPS in a volume of 100 µL PBS using a 27 G needle by intraperitoneal injection. Twenty-four hours later, mice were culled by cervical dislocation before spleens and PECs were harvested for further analysis.

### In vitro IL2-NK cell-BMDM co-culture

Splenic NK cell enrichment was done with MojoSort Mouse NK Cell Isolation Kit (480050, BioLegend). The NK cells were then cultured for 6 days in the presence of IL2 (NCI Clinical Repository, 200 ng/mL). On day 4, NK cells were fed with the same dose of IL2. BMDMs were cultured in parallel in 10% L929-conditioned medium. On day 6, BMDMs were left unstimulated or stimulated with LPS from *E. coli* (O111:B4, 19661, Cayman Chemical, 10 ng/mL) for 6 h before cultured IL2-NK cells were added in a 1:1 ratio for a further 18 h in the presence of IL2 (NCI Clinical Repository, 20 ng/mL) and IL12 (130096707, Miltenyi Biotec, 10 ng/mL). When C75 (10005270, Cayman Chemical, 40 µM) was used in the co-cultures, this was added for the initial 6 h stimulation before being washed to specifically treat BMDMs. TOFA (10005263, Cayman Chemical, 15 µM) and zaragozic acid A (ZA, 17452, Cayman Chemical, 10 µM) were added to the cultures for the entire duration of the co-culture. Trans-well co-cultures were performed using a 0.4 µm trans-well. IL2-NK cells were alternatively cultured in 70% BMDM-conditioned medium, following stimulation of BMDMs with LPS from *E. coli* (O111:B4, 19661, Cayman Chemical, 10 ng/mL) for 18 h.

### In vitro splenic NK cell-peritoneal macrophage co-culture

PECs were harvested and cultured for 4 h to allow for peritoneal macrophages to adhere to the culture dish. All non-adherent cells were removed. Splenic NK cell enrichment was done with MojoSort Mouse NK Cell Isolation Kit (480050, BioLegend). Splenic NK cells were then co-cultured for 18 h. Peritoneal macrophages were stimulated with LPS (O111:B4, 19661, Cayman Chemical, 10 ng/mL) in the co-culture. Splenic NK cells were stimulated with IL12 (130096707, Miltenyi Biotec, 10 ng/mL), IL15 (2101510UG, PeproTech, 10 ng/mL) and IL18 (9139, BioTechne, 50 ng/mL). Unstimulated NK cells were cultured in low-dose IL15 (2 ng/mL). When C75 (10005270, Cayman Chemical, 40 µM) was used in co-cultures, this was added for 2 h prior to the addition of NK cells and was washed out to ensure inhibition of FASN specifically in macrophages.

### Flow cytometry

Surface and viability stains were done at 4 °C for 20 min in the dark. Cells were fixed with Cyto-FAt Fix-Perm Buffer Set (426803, BioLegend) and stained intracellular in corresponding buffer. Where cytokine staining was required, cells were cultured in the presence of GolgiPlug™ (555029, BD Biosciences) for 4 h. Dead cells were excluded with Zombie NIR™ (1:1000, 423107, BioLegend). Cells were analysed with a Canto II flow cytometer (BD) and BD DIVA 8.0 software. Antibodies were used as follows: NK1.1 (PK136, PECy5, 108716, BioLegend, 1:200), NK1.1 (PK136, BV421, 108732, BioLegend, 1:200), NK1.1 (29A1.4, BV786, 137637, BioLegend, 1:200), CD3 (145-2C11, FITC, 11003185, eBioscience, 1:200) CD3 (145-2C11, APC, 100312, BioLegend, 1:200), CD3 (17A2, BV510, 100234, BioLegend, 1:200), CD3 (17A2, BV650, 100229, BioLegend, 1:200), CD19 (1D3, APC, 550992, BioLegend, 1:200), CD19 (1D3, BV650, 563235, BioLegend, 1:200), CD40 (3-23, FITC, 124608, Biolegend, 1:200), CD49b (DX5, BV421, 563063, BD Biosciences, 1:200), CD49b (DX5, PE, 553858, BD Biosciences, 1:200), CD11b (M1/70, PECy7, 552850, BD Biosciences, 1:1000), F4/80 (BM8, BV510, 123135, BioLegend, 1:400), IFNγ (XMG1.2, BV421, 505830, BioLegend, 1:100), perforin (S16009B, APC, 154404, BioLegend, 1:800), granzyme B (NGZM, PECy7, 25889882, eBioscience, 1:100), IL12p40/p70 (C15.6, V450, 561456, BD Biosciences, 1:100), IL6 (MP5-20F3, APC, 504504, BioLegend, 1:100), TNFα (MP6-XT22, PECy7, 25732182, Invitrogen, 1:100), CD69 (H1.2F3, APCCy7, 104526, BioLegend, 1:200), CD71 (C2, BV510, 563112, BD Biosciences, 1:200), CD36 (CRF D-2712, PE, 562702, BD Biosciences, 1:200), EOMES (Dan11mag, AF488, 53487482, eBioscience, 1:500, overnight stain), T-bet (eBio4B10, APC, 14585282, eBioscience, 1:500, overnight stain), pS6rp (D57.2.2E, APC, 14733S, Cell Signalling, 1:800, overnight stain).

Cells were resuspended in complete RPMI plus tetramethyl rhodamine methyl ester (TMRM, 100 nM, T668, Invitrogen) and nonylacridine orange (NAO, 100 nM, A3568, Invitrogen) and incubated for 30 min at 37 °C. Oligomycin (2 µM, Merck) and BAM15 (2.5 µM, Merck) were added to cells to induce hyperpolarisation or hypopolarisation, respectively. Cells were analysed with LSR Fortessa flow cytometer (BD) and BD DIVA 8.0 software. Cells were stained with LipidTOX™ Green (H34475, Invitrogen, 1X) for 20 min at 4 °C.

### Cytokine bead array

For measurements of cytokines in the peritoneal cavity, a peritoneal lavage was carried out using 4 mL PBS. For measurements of cytokine and chemokine levels in the culture medium of peritoneal macrophages, cells were stimulated with LPS (O111:B4, 19661, Cayman Chemical, 10 ng/mL) in the presence or absence of C75 (10005270, Cayman Chemical, 40 µM). A custom Legendplex™ cytokine bead array (BioLegend) was used to measure the levels of cytokines and chemokines in the lavage or cell culture supernatant, according to the manufacturer’s instructions and analysed using a NovoCyte (Agilent) flow cytometer.

### Transferrin uptake

Cells were washed with serum-free RPMI supplemented with 5% BSA and incubated with Transferrin-AlexaFluor647 (T23366, Invitrogen, 5 µg/mL) for 10 min at 37 °C. Unlabelled holotransferrin (T4132, Merck, 500 µg/mL) was used as a competition control. Uptake was stopped by washing with ice-cold acid wash (PBS supplemented with 150 mM NaCl and 20 mM citric acid, pH 5). Cells were stained for surface markers and analysed by flow cytometry.

### Kynurenine uptake

Cells were washed from culture medium and surface antibody stained before being resuspended in HBSS in the presence or absence of kynurenine (200 µM, K8525, Merck), 2-amino-2-norbornanecarboxylic acid (BCH, 10 mM A7902, Merck) or leucine (5 mM, L8912, Merck) for 5 min at 37 °C. Uptake was stopped by fixation with PFA (1%). Cells were washed with PBS and analysed on a Canto II flow cytometer (BD).

### Oleic acid alkyne click chemistry

Cells were washed with serum-free RPMI and stained for surface markers before being incubated with oleic acid alkyne (Van Kasteren Lab, Leiden Institute of Chemistry, 6 µM) for 10 min at 37 °C. Sodium oleate (143191, Merck, 60 µM) was used as a competition control. Uptake was stopped by washing cells with ice-cold acid wash and fixing with PFA (1%). Cells were permeabilised with perm buffer (PBS supplemented with 1% w/v BSA and 0.01% w/v saponin) before being treated with click chemistry mix (PBS containing 1 mM copper (II) sulfate, 10 mM sodium-L-ascorbate, 1 mM tris-hydroxypropyltriazolylmethylamine and 5 µM azide-AlexaFluor488) for 1 h at RT. Cells were washed three times with PBS and analysed on a Canto II flow cytometer (BD).

### Puromycin click chemistry

Cells were washed with HBSS and stained for surface markers before being incubated with O-propargyl puromycin (OPP) (NU93105, Jena Bioscience, 20 µM) for 30 min at 37 °C. When this was performed in the presence of etomoxir (11969, Cayman Chemical, 5 µM), cells were pre-treated for 10 min before OPP incorporation was performed in the presence of the inhibitor. Incorporation was stopped by fixing cells with PFA (1%). Cells were washed before being permeabilised with perm buffer (PBS supplemented with 1% w/v BSA and 0.01% w/v saponin) and treated with click chemistry mix (PBS containing 1 mM copper (II) sulfate, 10 mM sodium-L-ascorbate, 1 mM tris-hydroxypropyltriazolylmethylamine and 5 µM azide-AlexaFluor488) for 1 h at RT. Cells were washed three times with PBS and analysed on a Canto II flow cytometer (BD).

### Fluorescence release cytotoxicity assay

RMA/S target cells were stained with calcein AM (564061, BD Biosciences, 10 µM) for 30 min at 37 °C. Cells were then washed and co-cultured with NK cells in a V-bottom plate in NK cell to target cell ratios of 5:1, 2:1 and 1:1. For measurements of spontaneous and maximum cell death, only target cells were added to the well, and they were left untreated or treated with Triton-X-100 (0.2%). Cells were incubated for 3 h at 37 °C. Following incubation, culture supernatants were taken and transferred to a black plate. Fluorescence of released calcein AM in the supernatant was measured on a FLUOstar OPTIMA microplate reader (BMG LabTech), with an excitation wavelength of 495 nm and an emission wavelength of 515 nm. Specific lysis was calculated as: %specific lysis = ((exp-spon)/(max-spon)) × 100.

### Iron measurements by GFAAS

Cells were washed from culture medium and were resuspended in PBS in metal-free tubes containing 1% Triton-X-100. Cell-free samples were used as background controls for each experiment. Samples were digested by adding a digestion buffer, 50% nitric acid by volume with 0.1% digitonin (Sigma-Aldrich D141) to the sample in a 1:1 ratio, then heated at 70 °C for 2 h in a heat block. The digested samples were cooled to room temperature and centrifuged for 5 min at 6000 × *g*. The supernatant was collected and then diluted in dilute 0.2% nitric acid for measurement via the graphite furnace atomic absorption spectrometer (PerkinElmer model 900z). Iron is measured in the digestion buffer to assess iron contamination, and sample iron levels are quantified using a standard curve of known iron concentrations.

### Confocal microscopy

NK cells were washed from culture medium and fixed with PFA (1%) for 15 min at RT. Cells were washed and stained with LipidTOX™ Deep Red (H34477, Invitrogen, 1×) and DAPI (10236276001, Merck, 1 µg/mL) for 1 h at 4 °C in the dark. Cells were washed with PBS and resuspended in Mowiol®4-88 (475904, Merck) mounting medium and embedded on a glass microscope slide. Cells were imaged using a Leica TCS SP8 STED (Leica Microsystems) using LASX software. LipidTOX™ Deep Red was excited at 630 nm and the emission was detected at 650 nm. DAPI was excited at 360 nm and the emission was detected at 460 nm.

### Real-time PCR

RNA lysates were obtained and stored using the RNeasy mini kit (74104, Qiagen) according to the manufacturer’s instructions. RNA purity and abundance was measured using a Nanodrop spectrophotometer (ND-100, Nanodrop® Technologies). cDNA was synthesised using q-Script supermix (95047-025, Quantabio) and reverse transcription was carried out using a ^3^Prime Thermal Cycler (Techne). Real-time PCR was performed using iQ SYBR Green (163795753, VWR) based detection on a QuantStudio 3 RT-qPCR (Applied Biosciences) system. Relative changes in RNA were calculated by the Δ*C*_t_ method: 2^((*C*_t_ of endogenous control gene) − (*C*_t_ of gene of interest)). PCR primer sequences are included in Supplementary Table 1.

### Quantification of diacylglycerols and triacylglycerols

NK cells were purified prior to co-culture with BMDMs for 18 h. Following co-culture 3 × 10^6^ NK cells were harvested and snap-frozen in liquid nitrogen. The metabolites were extracted in isopropanol, sonicated, centrifuged and prepared for analysis. The diacylglycerols and triacylglycerols were semi-quantified by FIA/MS using MxP® Quant 500 XL kit (Biocrates) in the Brennan Lab, University College Dublin.

### Cell culture supernatant metabolomics

BMDMs were left unstimulated or stimulated for 18 h in the presence of LPS from *E. coli* (O111:B4, 19661, Cayman Chemical, 10 ng/mL). IL2-NK cells were stimulated with IL2 (NCI Clinical Repository, 20 ng/mL) and IL12 (130096707, Miltenyi Biotec, 10 ng/mL) for 18 h. The cells were then co-cultured for a further 24 h in serum-free medium. Cell culture supernatants were harvested and snap frozen in liquid nitrogen before quantification of metabolites by LC/MS and FIA/MS using the MxP® Quant 500 XL kit (Biocrates) in the Brennan Lab, University College Dublin. Concentration changes in the co-culture supernatants are versus serum-free medium controls.

### Statistical analysis

GraphPad Prism (10.4.1) (GraphPad Software) was used for statistical analysis. A two-tailed Welch’s t-test (parametric) was used for two groups with unequal variance. A two-tailed Mann–Whitney *U* test (non-parametric) was used for analysis of two groups. A one-way ANOVA (parametric) was used for analysis of more than two groups with Tukey’s post-test for multiple comparisons. A Kruskal–Wallis test (non-parametric) was used to compare more than two groups with Dunn’s post-test for multiple comparisons. Analysis of data with more than two groups and two independent variables was carried out using a two-way ANOVA. When data with two independent variables required multiple comparisons within groups, a Šidák’s post-test was performed. When data with two independent variables required multiple comparisons across groups, a Tukey’s post-test was performed. A *p* < 0.05 was considered statistically significant, *p < 0.05, **p < 0.01 and ***p < 0.001.

### Reporting summary

Further information on research design is available in the Nature Portfolio Reporting Summary linked to this article.

## Supplementary information

Supplementary Information

Description of Additional Supplementary Files

Supplementary Data 1

Supplementary Data 2

Reporting Summary

Transparent Peer Review File

## Source data

Source Data

## Data Availability

The authors declare that the supporting findings of this study are available within the paper. All Source data is linked to the article. Source data are provided with this paper.
