# Supplementary material for: Macrophages tune NK cell IFNγ production through direct lipid transfer
Source: Nat Commun. 2026 Aug 8;17:9527. doi: 10.1038/s41467-026-76444-0 (PMC13547247; doi:10.1038/s41467-026-76444-0)
Supplement: Supplementary file 1 [file 41467_2026_76444_MOESM1_ESM.pdf]

# Macrophages Tune NK Cell IFNγ Production Through Direct Lipid Transfer

Cathal Keane, Shauna Mayock, Carrie Corkish, Ella Rogerson, Cristhiane Favero de Aguiar, Ya Gao, Sarah Kenny, Lorraine Brennan, Suzanne M. Cloonan, David K. Finlay.

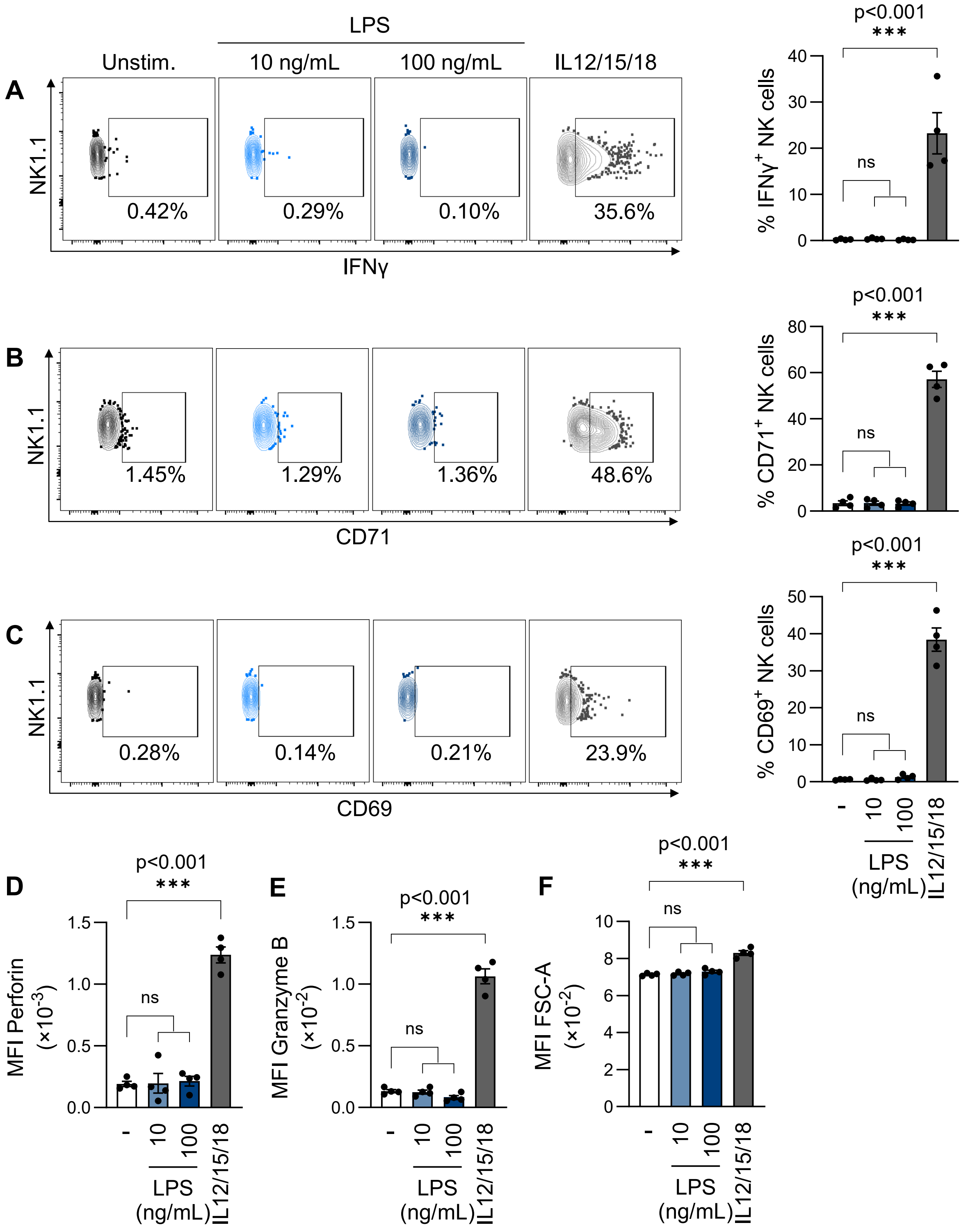


**Supplementary Figure 1**

(A-F) Splenic NK cells were MACS purified and stimulated with LPS (10 ng/mL, 100 ng/mL) for 24 h. Unstimulated NK cells were maintained in low-dose IL15 (2 ng/mL). NK cells were stimulated with IL12 (10 ng/mL), IL15 (10 ng/mL) and IL18 (50 ng/mL) as a positive control. IFNγ (A), CD71 (B), CD69 (C), perforin (D), granzyme B (E) and FSC-A (F) were used as measures of NK cell activation and were measured by flow cytometry. Data is representative of 4 biological replicates. Statistical analysis was performed using a one-way ANOVA with Tukey's post-test.

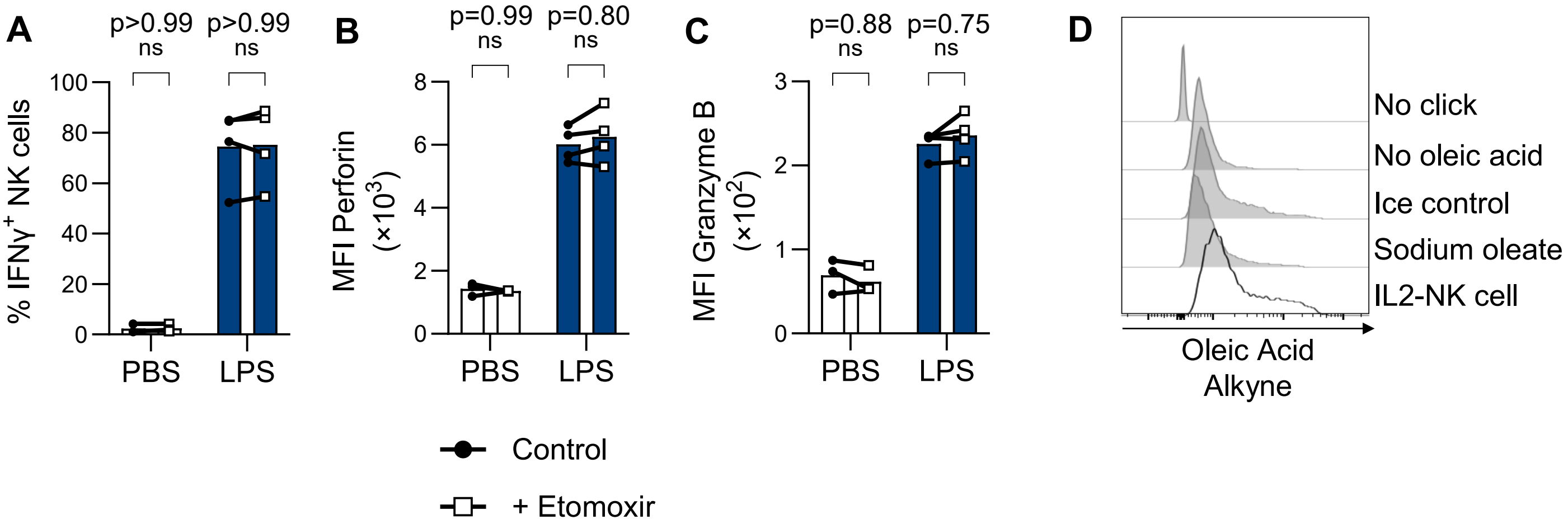


**Supplementary Figure 2**

(A-C) C57BL/6 mice were administered LPS (2.4 mg/kg) or PBS by intraperitoneal injection. 24 h later mice were sacrificed, and PECs were harvested by peritoneal lavage. PECs were treated with etomoxir (5 μM) for 4 h prior to measurement of IFNγ, perforin and granzyme B by flow cytometry. Statistical analysis was performed using a two-way repeated measures ANOVA with Šidák's post-test. (D) Oleic acid alkyne (6 μM) uptake by IL2-NK cells was measured using click chemistry by flow cytometry. No click and no oleic acid controls were used to confirm specificity of the probe. Uptake was performed on ice to confirm active transport. Sodium oleate (NaOleate) (60 μM) was used as a competition control.

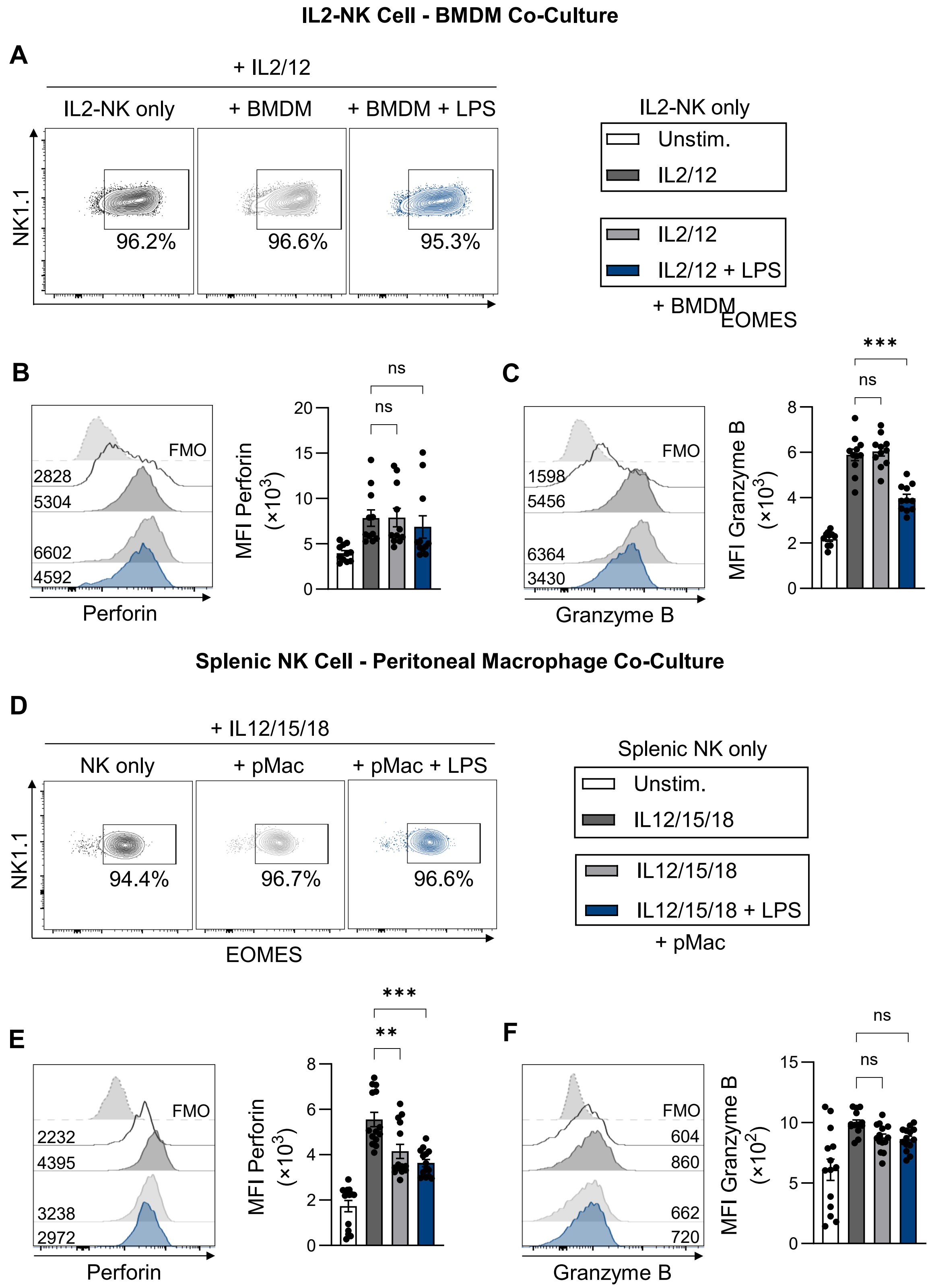


**Supplementary Figure 3**

(A-C) BMDMs were stimulated with LPS (10 ng/mL) or left unstimulated for 6 h. Then IL2-NK cells were directly co-cultured with BMDMs for 18 h in the presence of NK cell-activating cytokines IL2 (20 ng/mL) and IL12 (10 ng/mL). (D-F) NK cells were MACS purified from the spleen and were stimulated with IL12 (10 ng/mL), IL15 (10 ng/mL) and IL18 (50 ng/mL) for 18 hours in the presence or absence of macrophages which were purified from the peritoneal cavity. Macrophages were left unstimulated or stimulated with LPS (10 ng/mL). (A,D) EOMES expression by NK cells was measured by flow cytometry. Data is representative of 6 biological replicates across 2 independent experiments. (B,C,E,F) Perforin and granzyme B expression by NK cells was measured by flow cytometry. Pooled data is mean ± SEM of 11 biological replicates (B,C) or 14 biological replicates (E,F) across 4 independent experiments (B,C) or 5 independent experiments (E,F). Statistical analysis was performed using a one-way ANOVA with Tukey's post-test.

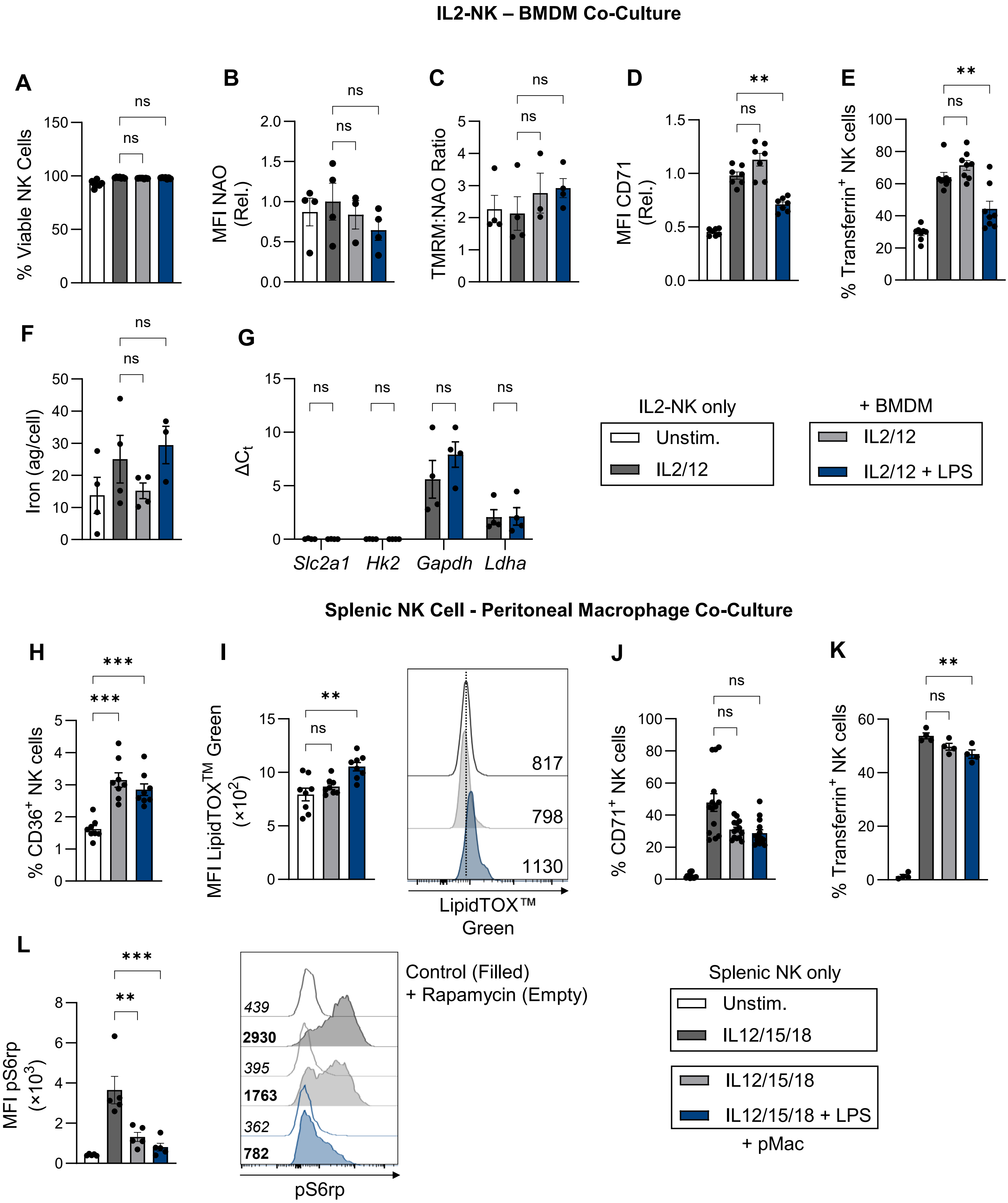

IL2-NK – BMDM Co-Culture
A
% Viable NK Cells
ns
B
MFI NAO (Rel.)
C
TMRM:NAO Ratio
D
MFI CD71 (Rel.)
**
E
% Transferrin+ NK cells
F
Iron (ag/cell)
G
ΔCt
Slc2a1
Hk2
Gapdh
Ldha
IL2-NK only
Unstim.
IL2/12
+ BMDM
IL2/12
IL2/12 + LPS
Splenic NK Cell - Peritoneal Macrophage Co-Culture
H
% CD36+ NK cells
***
I
MFI LipidTOX™ Green (×10²)
817
798
1130
LipidTOX™ Green
J
% CD71+ NK cells
K
% Transferrin+ NK cells
L
MFI pS6rp (×10³)
Control (Filled)
+ Rapamycin (Empty)
439
2930
395
1763
362
782
pS6rp
Splenic NK only
Unstim.
IL12/15/18
IL12/15/18
IL12/15/18 + LPS
+ pMac

**Supplementary Figure 4**

(A-G) BMDMs were stimulated with LPS (10 ng/mL) or left unstimulated for 6 h. Then IL2-NK cells were directly co-cultured with BMDMs for 18 h in the presence of NK cell-activating cytokines IL2 (20 ng/mL) and IL12 (10 ng/mL). (H-L) NK cells were MACS purified from the spleen and were left unstimulated or stimulated with IL12 (10 ng/mL), IL15 (10 ng/mL) and IL18 (50 ng/mL) for 18 hours in the presence or absence of macrophages which were purified from the peritoneal cavity. Macrophages were left unstimulated or stimulated with LPS (10 ng/mL). (A) NK cell viability was measured using Zombie NIR™ viability dye by flow cytometry. Data is mean +/- SEM of 7 biological replicates across 3 independent experiments. (B,C) Nonylacridine orange (NAO, 100 nM) and TMRM (100 nM) staining of IL2-NK cells was carried out before measurement by flow cytometry. Data is mean ± SEM of 4 biological replicates across 2 independent experiments. (D,J) CD71 expression was measured by flow cytometry. Data is mean ± SEM of 7 biological replicates (D) or 14 biological replicates (J) across 3 independent experiments (D) or 5 independent experiments (J). (E,K) Transferrin-AF647 (5 µg/mL) uptake was performed before measurement by flow cytometry. Data is mean ± SEM of 8 biological replicates across 3 independent experiments (E) or 4 biological replicates (K). (F) Iron content was measured in purified NK cells by GFAAS and was normalised to cell count. Data is mean ± SEM of at least 3 biological replicates across 3 independent experiments. (G) Purified IL2-NK cells were analysed by RT-qPCR for expression of Slc2a1, Hk2, Gapdh, and Ldha mRNA and normalised to controls genes Rplp0 and Hprt. Data is mean +/- SEM of 4 biological replicates across 2 independent experiments. (H) CD36 staining was measured by flow cytometry. Data is mean ± SEM of 8 biological replicates across 2 independent experiments. (I) LipidTOX™ staining was measured by flow cytometry. Data is mean ± SEM of 8 biological replicates across 2 independent experiments. (L) Phosphorylation of S6rp was measured by flow cytometry. Cells were treated with rapamycin (20 nM) for 20 mins to confirm mTORc1 activity. Pooled data is mean +/- SEM of 5 biological replicates across 2 independent experiments. Statistical analysis was performed using a one-way ANOVA with Tukey's post-test (A-F,H-L) or unpaired t-tests (G).

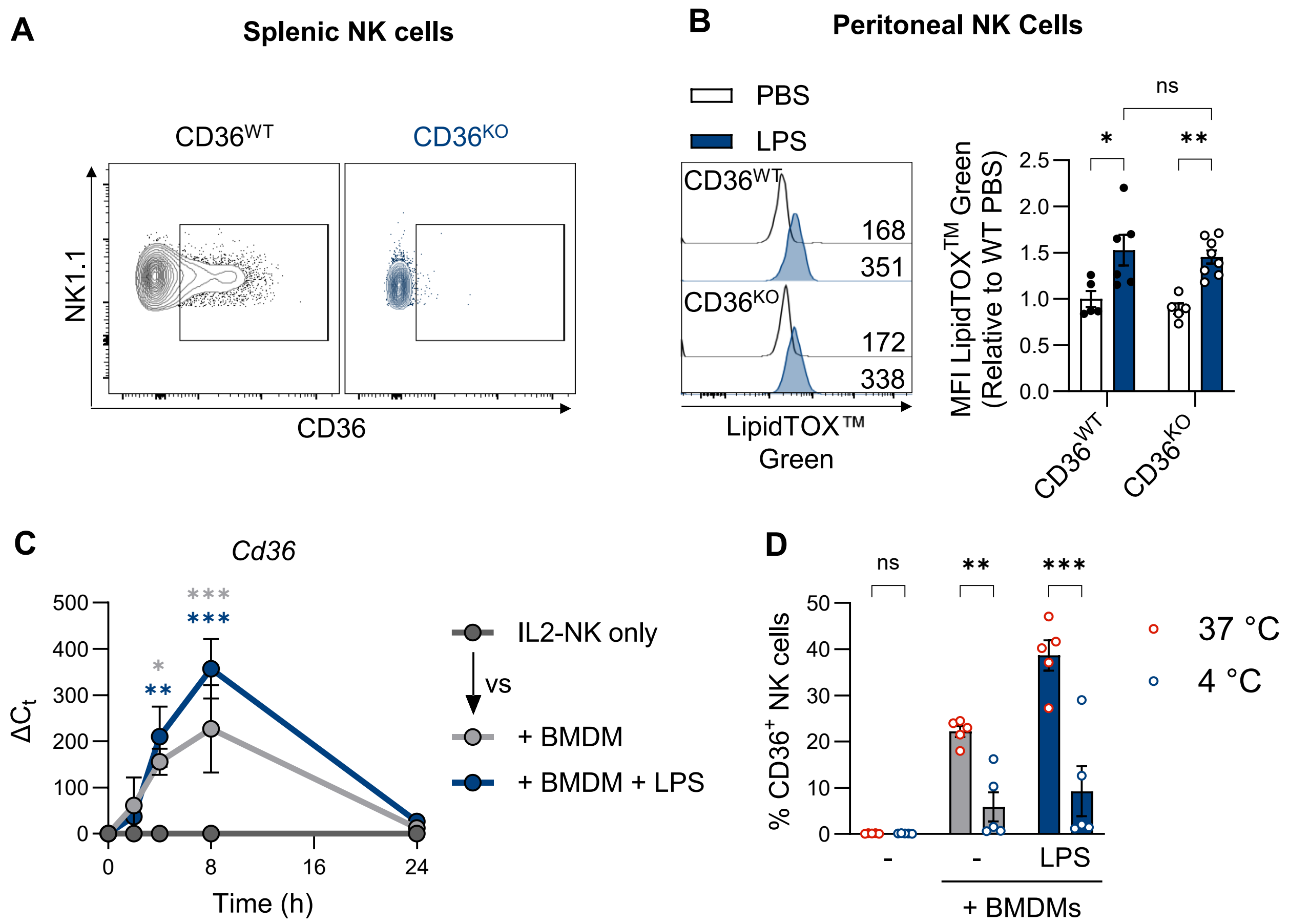


**Supplementary Figure 5**

(A) Age and sex matched CD36$^{WT}$ and CD36$^{KO}$ mice were sacrificed before spleens were harvested and CD36 expression was measured in splenocytes by flow cytometry. (B) Age and sex matched CD36$^{WT}$ and CD36$^{KO}$ mice were administered LPS (2.4 mg/kg) or PBS by intraperitoneal injection. 24 h later mice were sacrificed, and PECs were harvested by peritoneal lavage. LipidTOX™ green staining was measured by flow cytometry. (C) BMDMs were stimulated with LPS (10 ng/mL) or left unstimulated for 6 h. Then IL2-NK cells were directly co-cultured with BMDMs for 18 h in the presence of NK cell-activating cytokines IL2 (20 ng/mL) and IL12 (10 ng/mL). Purified NK cells were analysed by RT-qPCR for expression of *Cd36* mRNA and normalised to control genes *Gapdh* and *Rplp0*. Data is mean +/- SEM of 3 biological replicates. (D) BMDMs were stimulated with LPS (10 ng/mL) or left unstimulated for 6 h. Then IL2-NK cells were directly co-cultured with BMDMs for 8 h in the presence of NK cell-activating cytokines IL2 (20 ng/mL) and IL12 (10 ng/mL). Co-cultures were carried out at either 37°C or 4°C. LipidTOX™ staining was measured by flow cytometry. Data is mean ± SEM of 6 biological replicates across 2 independent experiments. Statistical analysis was performed using a two-way ANOVA with Tukey's post-test (B,C) or Šidák's post-test (D).

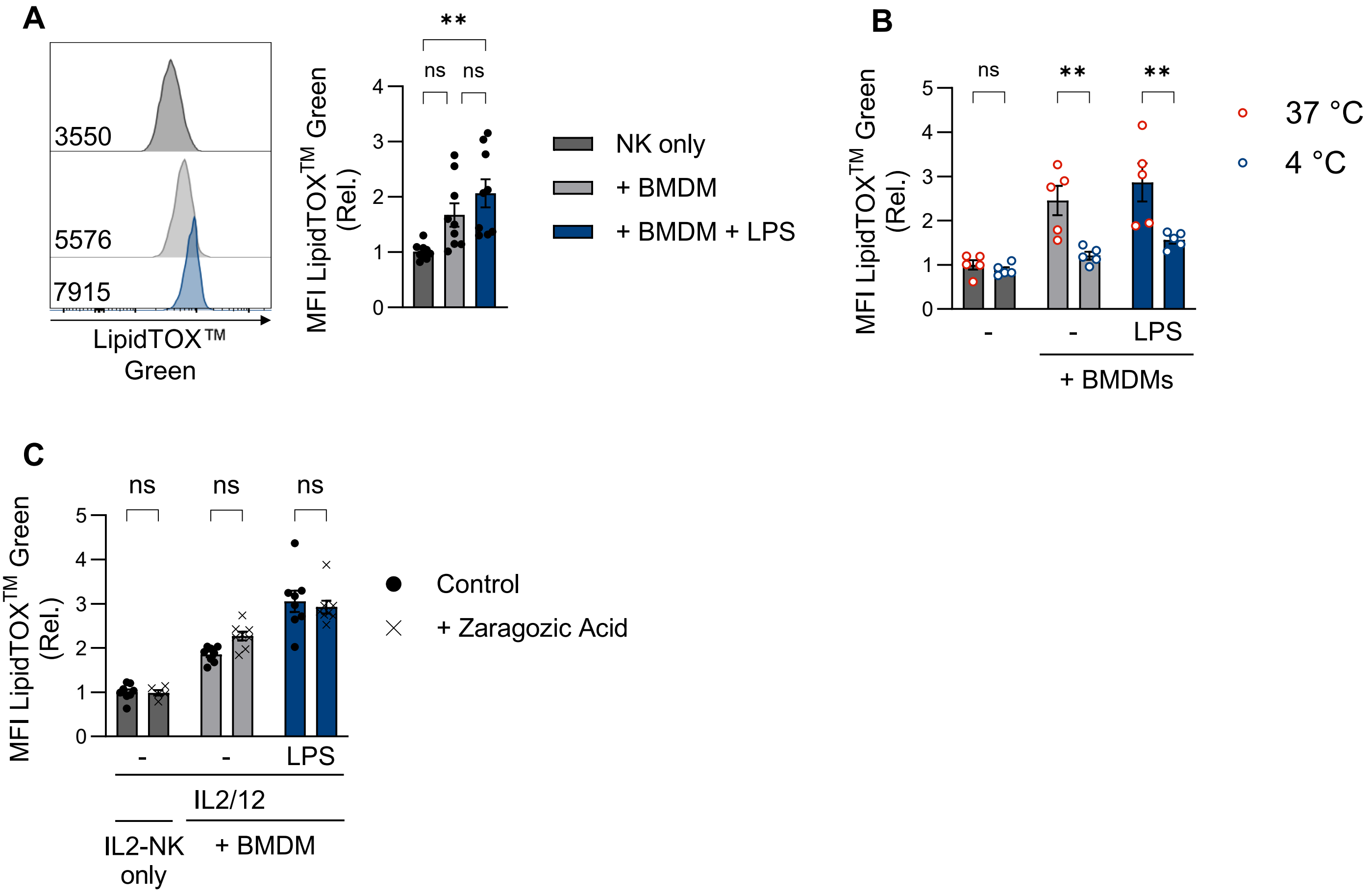


**Supplementary Figure 6**

(A) BMDMs were stimulated with LPS (10 ng/mL) or left unstimulated for 6 h. Then IL2-NK cells were co-cultured with BMDMs for 8 h in the presence of IL2 (20 ng/mL) and IL12 (10 ng/mL). LipidTOX™ staining was measured by flow cytometry. Data is mean ± SEM of 9 biological replicates across 3 independent experiments. (B) BMDMs were stimulated with LPS (10 ng/mL) or left unstimulated for 6 h. Then IL2-NK cells were co-cultured with BMDMs for 8h in the presence of IL2 (20 ng/mL) and IL12 (10 ng/mL). Co-Cultures were performed at 37°C or 4°C. ). LipidTOX™ staining was measured by flow cytometry. Data is mean ± SEM of 5 biological replicates across 2 independent experiments. (C) BMDMs were stimulated with LPS (10 ng/mL) or left unstimulated for 6 h. Then IL2-NK cells were co-cultured with BMDMs for 18 h in the presence of IL2 (20 ng/mL) and IL12 (10 ng/mL) in the presence or absence of zaragozic acid A (10 μM). LipidTOX™ staining was measured by flow cytometry. Data is mean ± SEM of 8 biological replicates across 3 independent experiments. Statistical analysis was performed using a one-way ANOVA with Tukey=s post-test (A) or a two-way ANOVA with Šidák's post-test (B,C).

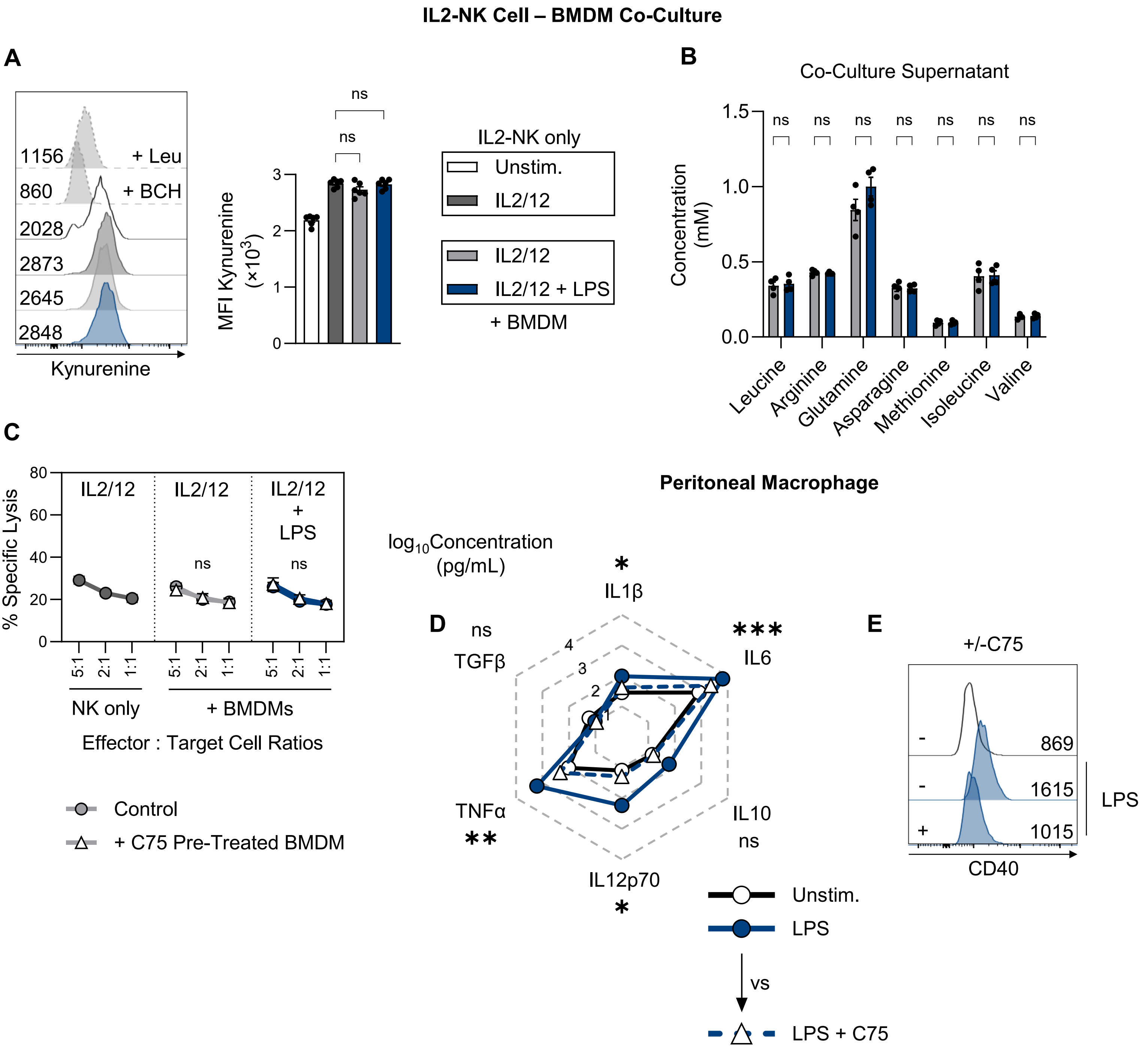


**Supplementary Figure 7**
(A,B) BMDMs were stimulated with LPS (10 ng/mL) or left unstimulated for 6 h. Then IL2-NK cells were co-cultured with BMDMs for 18 h in the presence of IL2 (20 ng/mL) and IL12 (10 ng/mL). (A) Kynurenine (200 µM) uptake was carried out in the presence or absence of leucine (5 mM) or BCH (10 mM) and was measured by flow cytometry. Pooled data is mean ± SEM of 6 biological replicates across 2 independent experiments. (B) Co-culture supernatants were harvested, and amino acids were quantified by LC/MS. Data is mean ± SEM of 4 biological replicates. (C) BMDMs were stimulated with LPS (10 ng/mL) or left unstimulated for 6 h in the presence or absence of C75 (40 µM). C75 was washed off before IL2-NK cells were co-cultured with BMDMs for 18 h in the presence of NK cell-activating cytokines IL2 (20 ng/mL) and IL12 (10 ng/mL). NK cell cytotoxicity against RMA/S target cells was measured by fluorescence release cytotoxicity assay. Data is mean ± SEM of 4 biological replicates across 2 independent experiments. (D,E) Macrophages were purified from the peritoneal cavity and pre-treated for 2 h with C75 (40 µM). C75 was then washed off before macrophages were left unstimulated or stimulated with LPS (10 ng/mL) for a further 18 h. Data is mean ± SEM of 4 biological replicates. Cytokine production was measured in cell culture supernatants using a cytokine bead array (D). CD40 expression by peritoneal macrophages was measured using flow cytometry (E). Statistical analysis was performed using a one-way ANOVA with Tukey's post-test (A,D) unpaired t-tests (B) or a two-way ANOVA (C).

Peritoneal NK cells *ex vivo* – Functional Panel

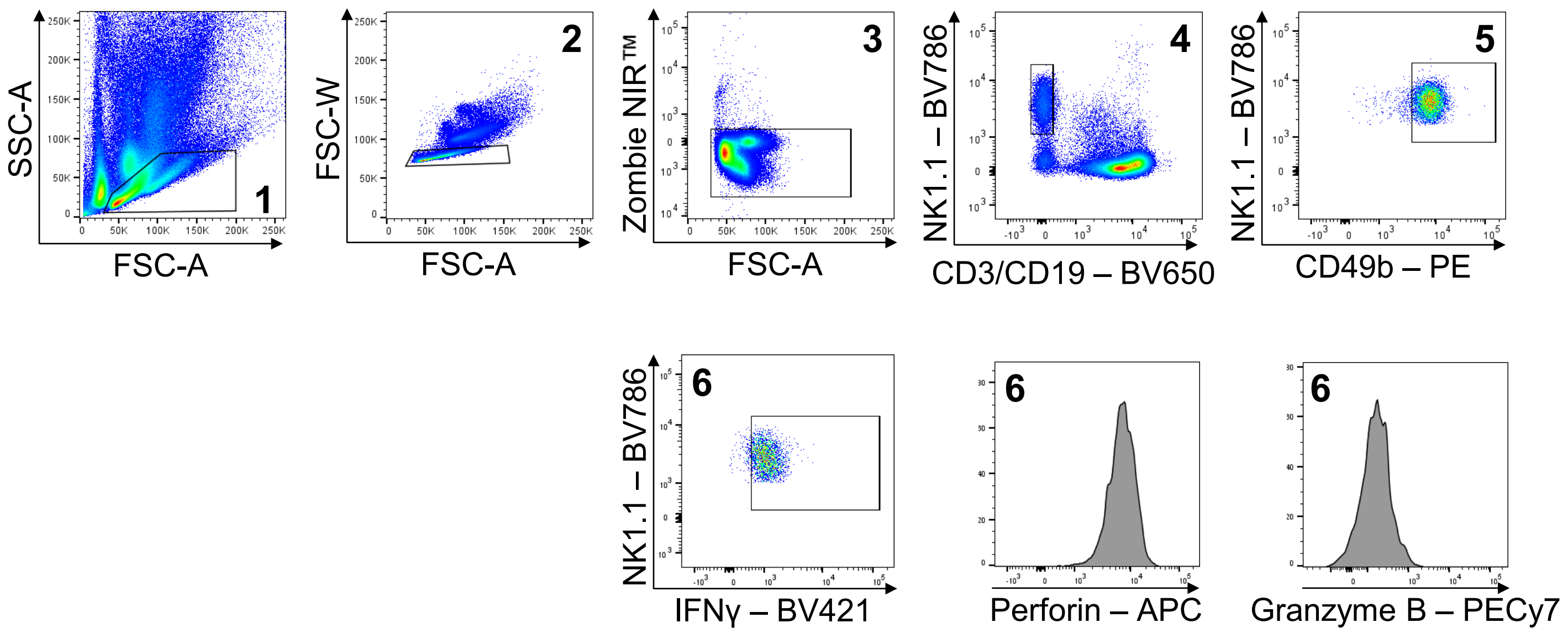


Peritoneal Macrophages *ex vivo* – Functional Panel

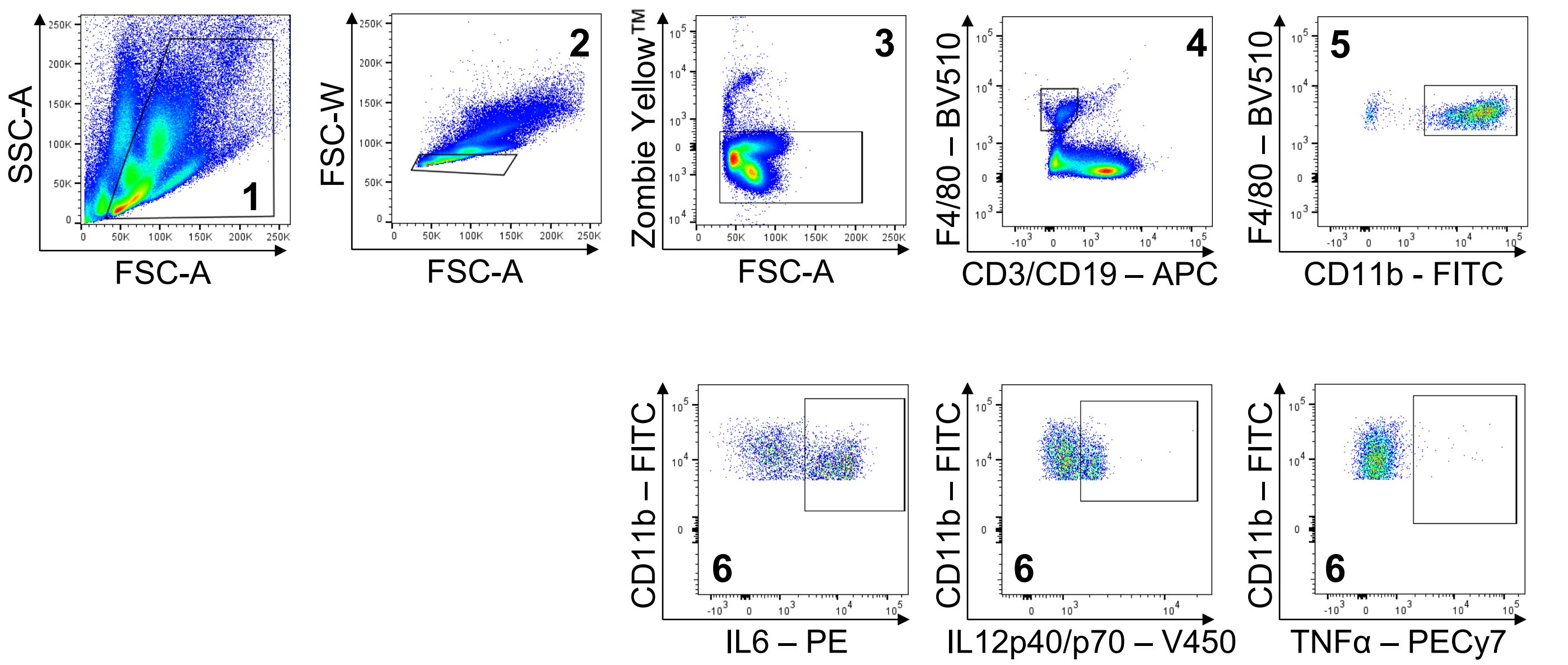


Peritoneal NK cells *ex vivo* – Click Chemistry Panel

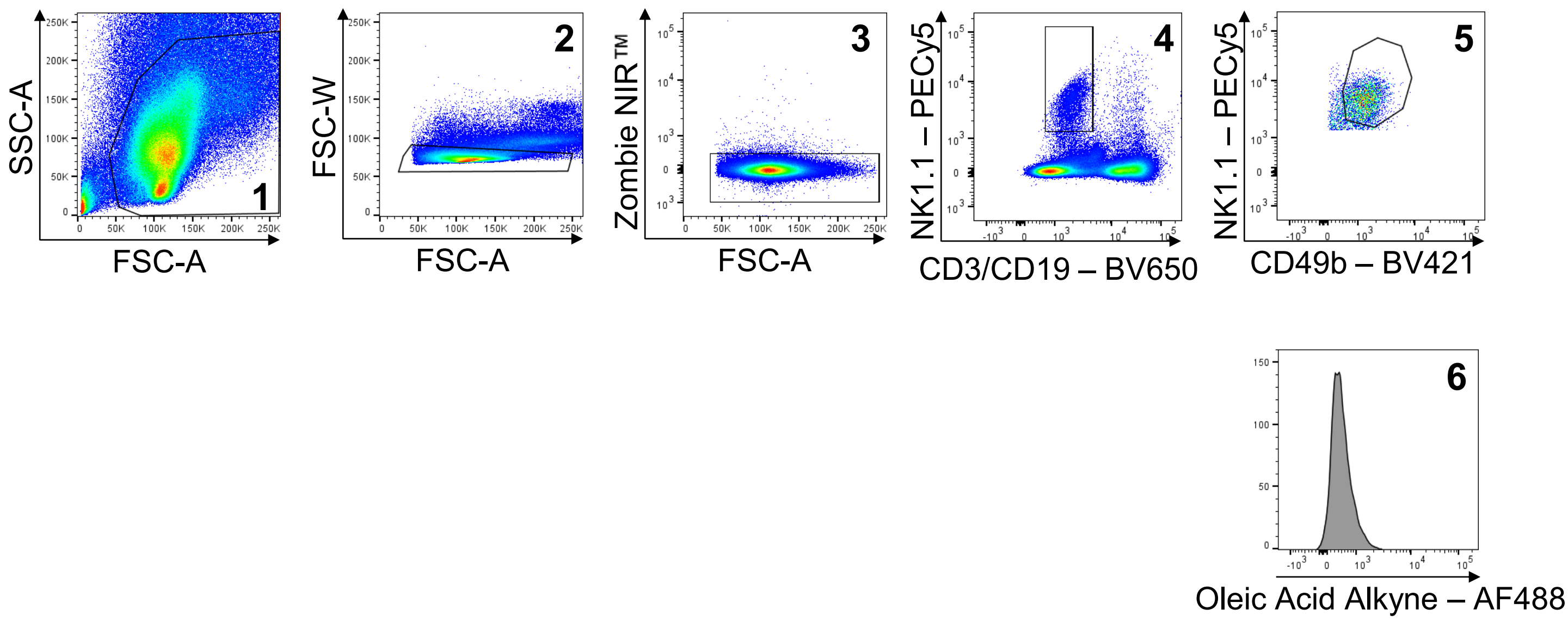

Peritoneal NK cells *ex vivo* – Mitochondrial Parameters

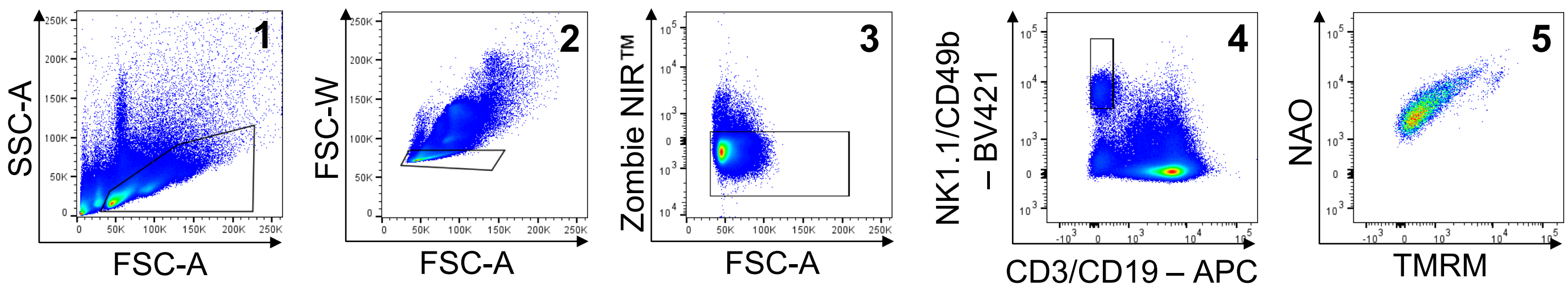


Peritoneal NK cells *ex vivo* – LipidTOX™ Green and CD36

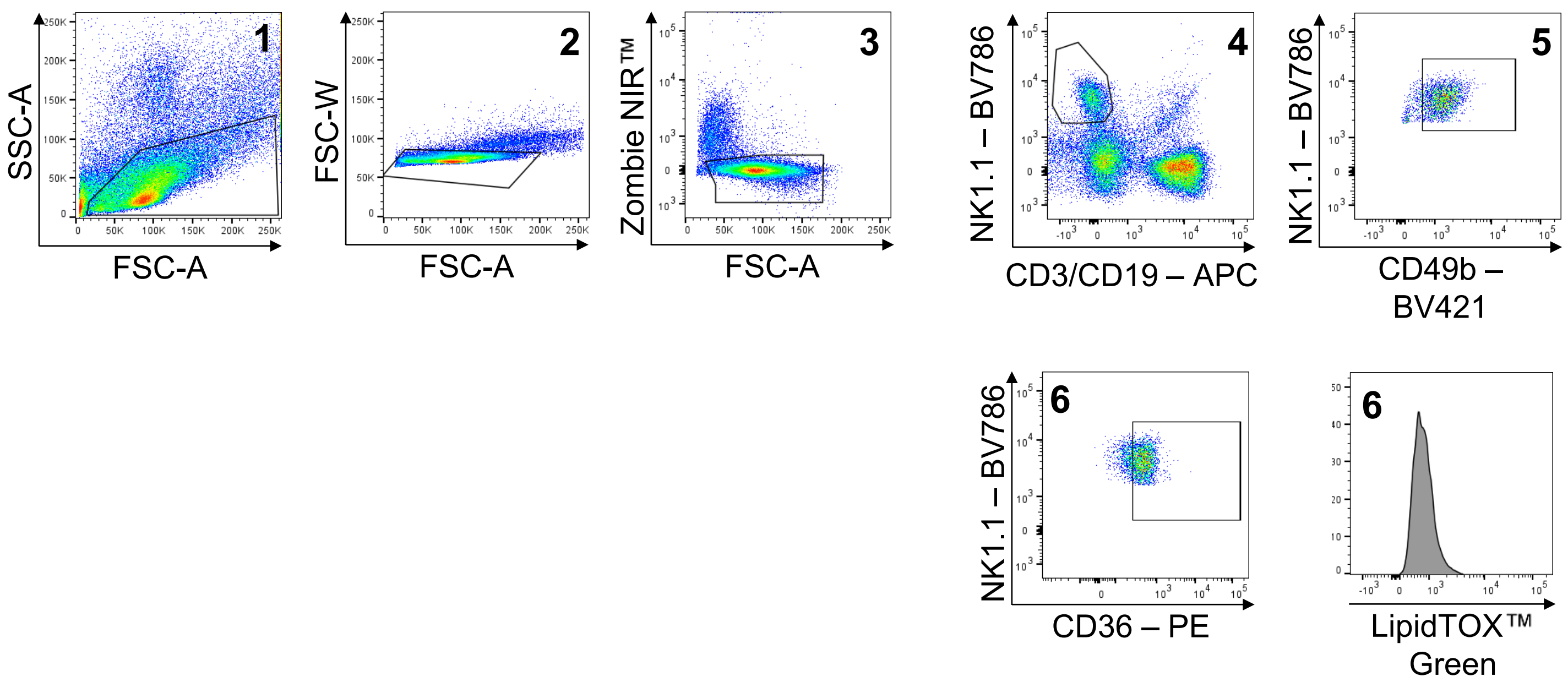


Peritoneal NK cells *ex vivo* – Transferrin Uptake

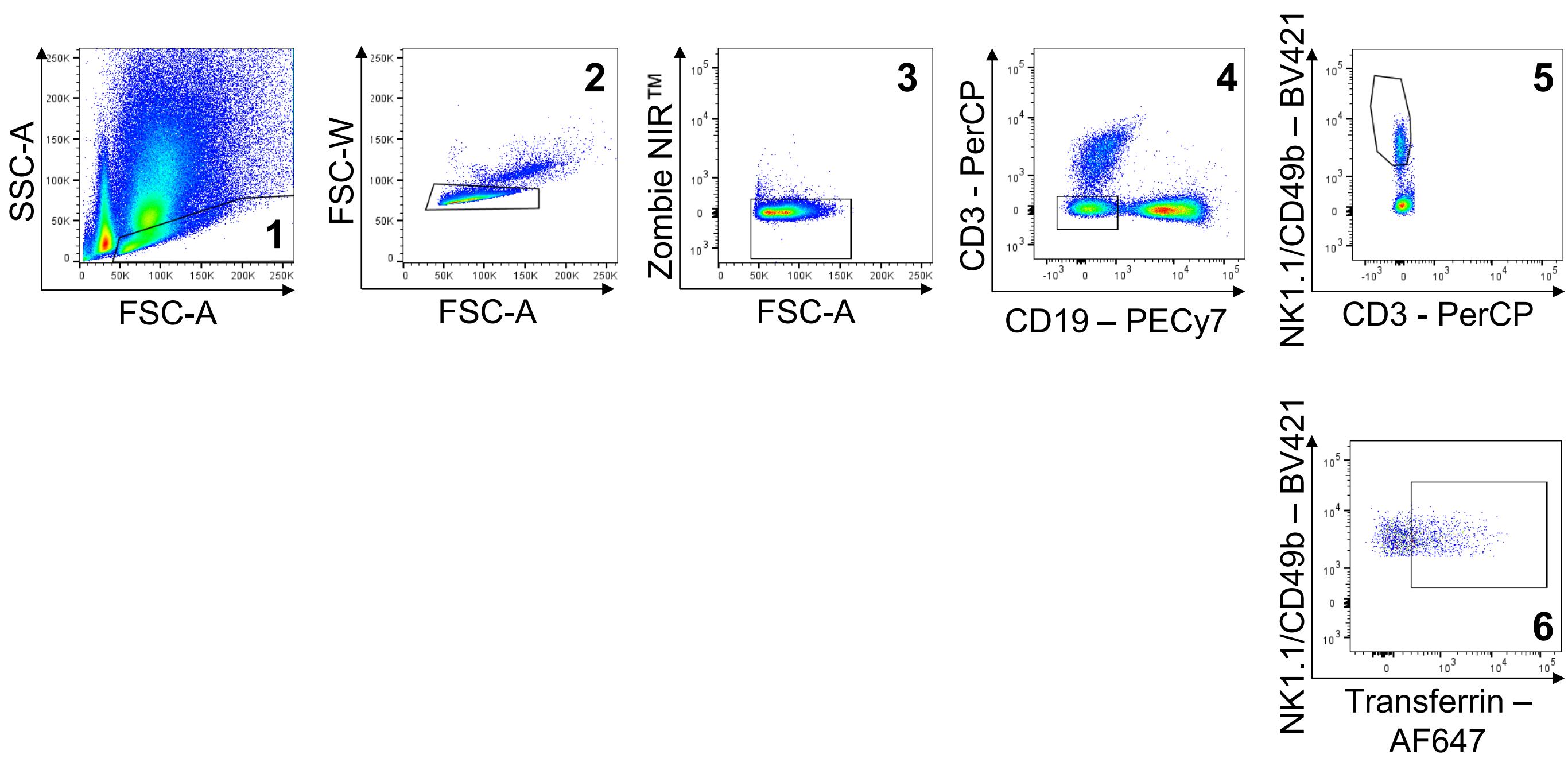

Transferrin Uptake Panel – Cultured NK cells

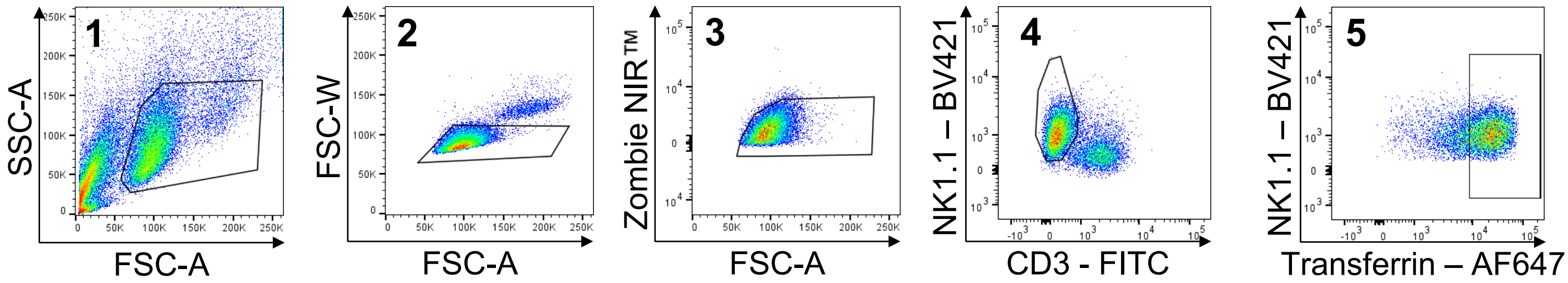


EOMES and T-bet – Cultured NK cells

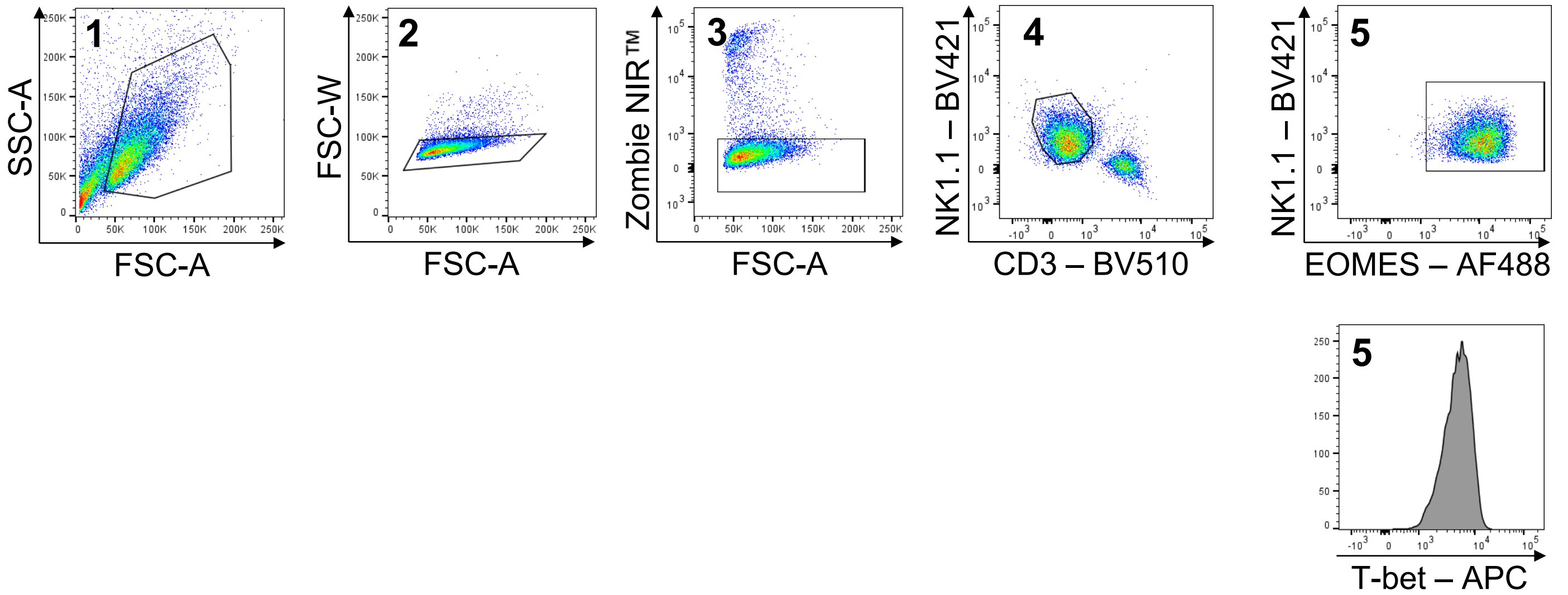


Click Chemistry Panel – Cultured NK cells

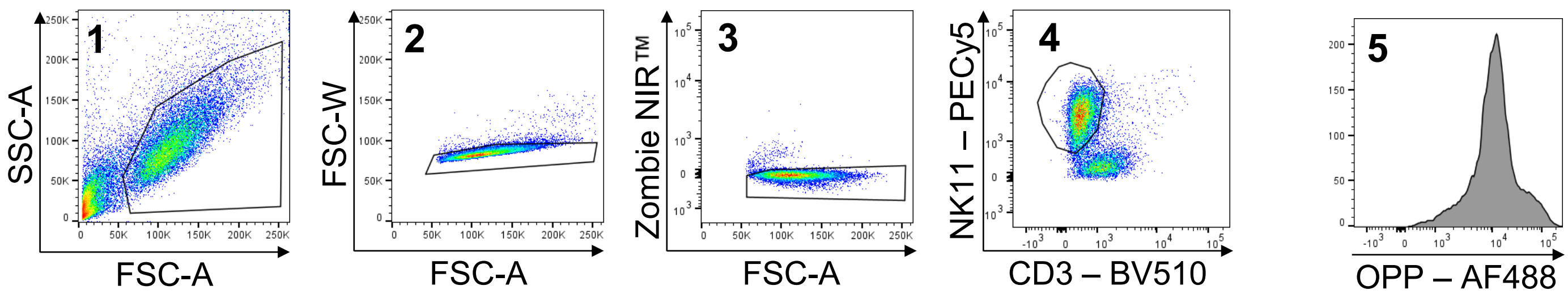


Mitochondrial Parameters – Cultured NK cells

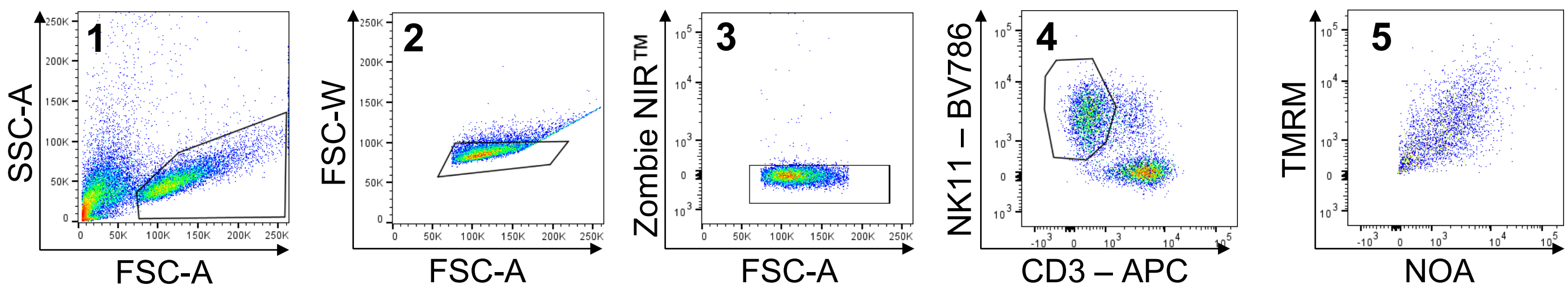


Peritoneal Macrophages – Cultured

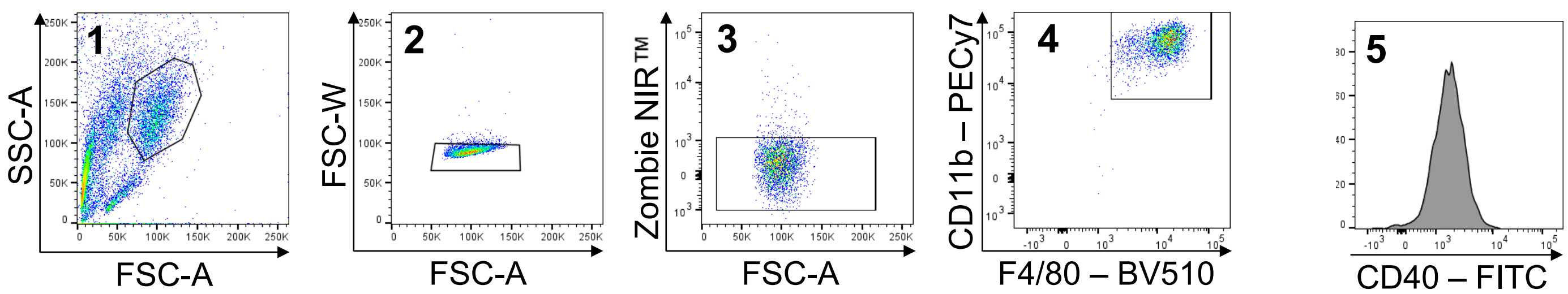

## Kynurenine Uptake Panel – Cultured NK cells

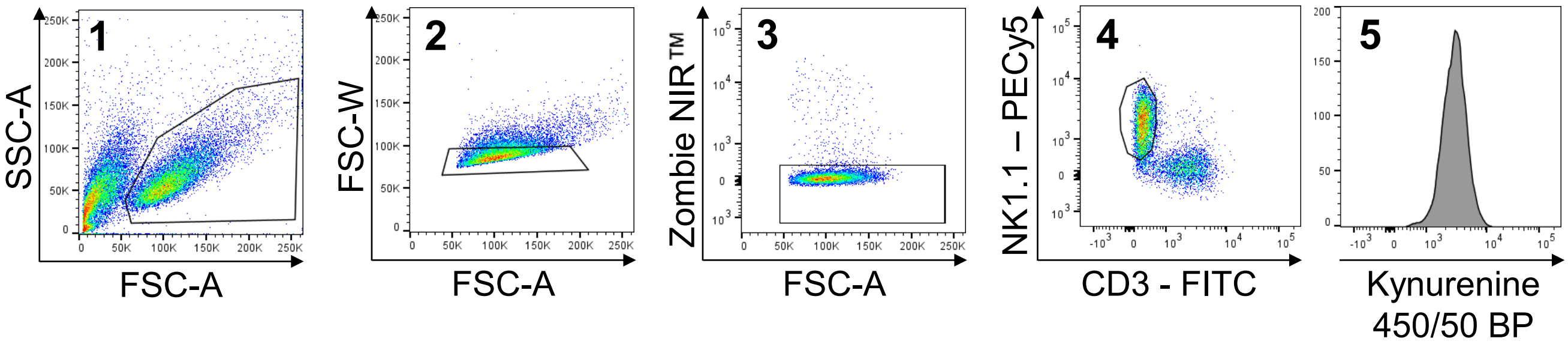


## pS6rp Panel – Cultured NK cells

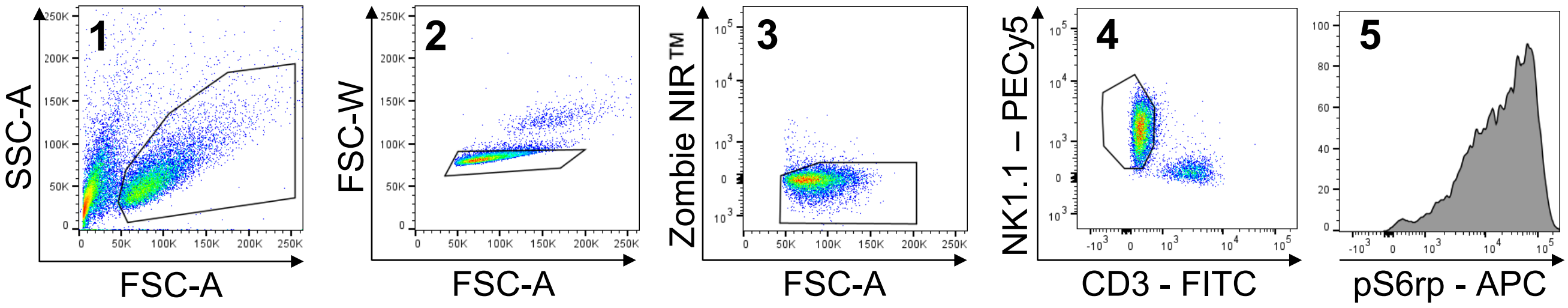


## LipidTOX and CD36 – Cultured NK cells

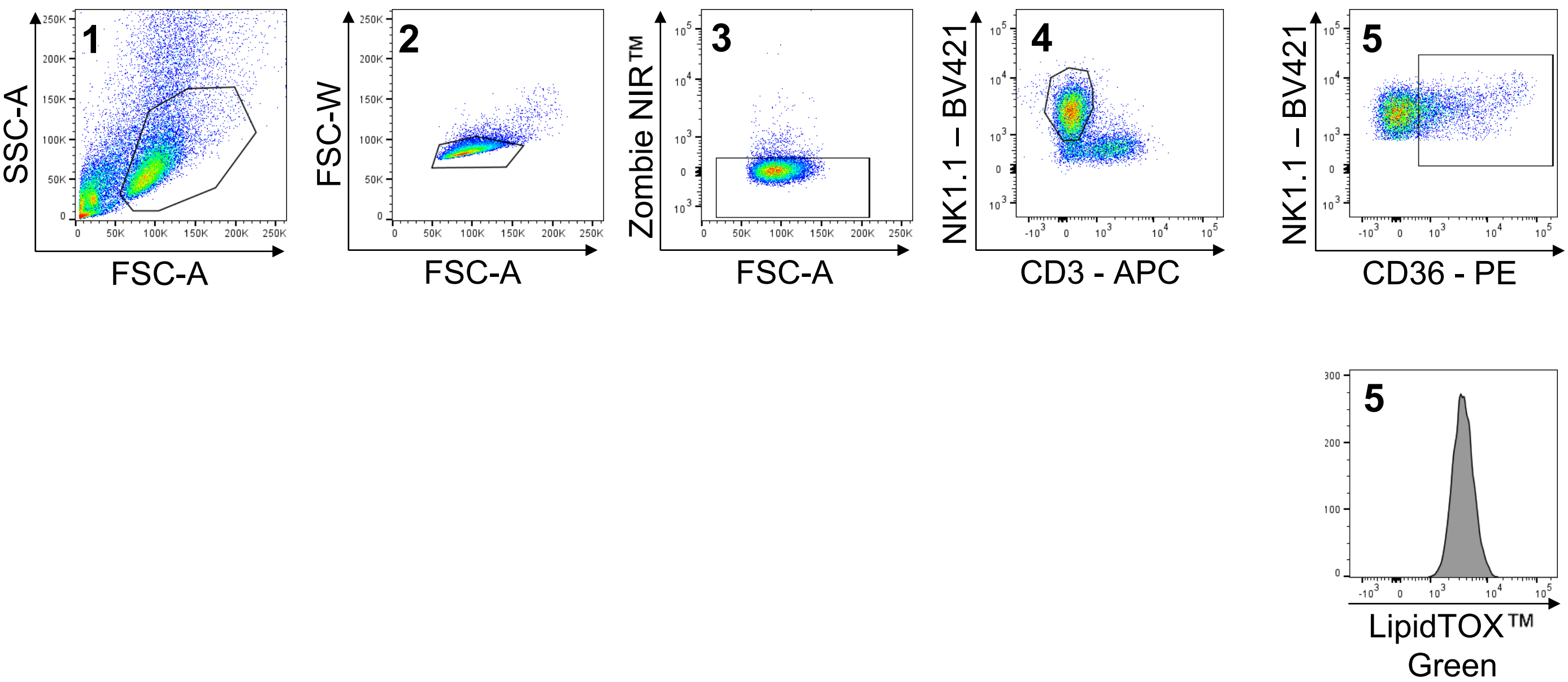


## LipidTOX and CD36 – Cultured NK cells

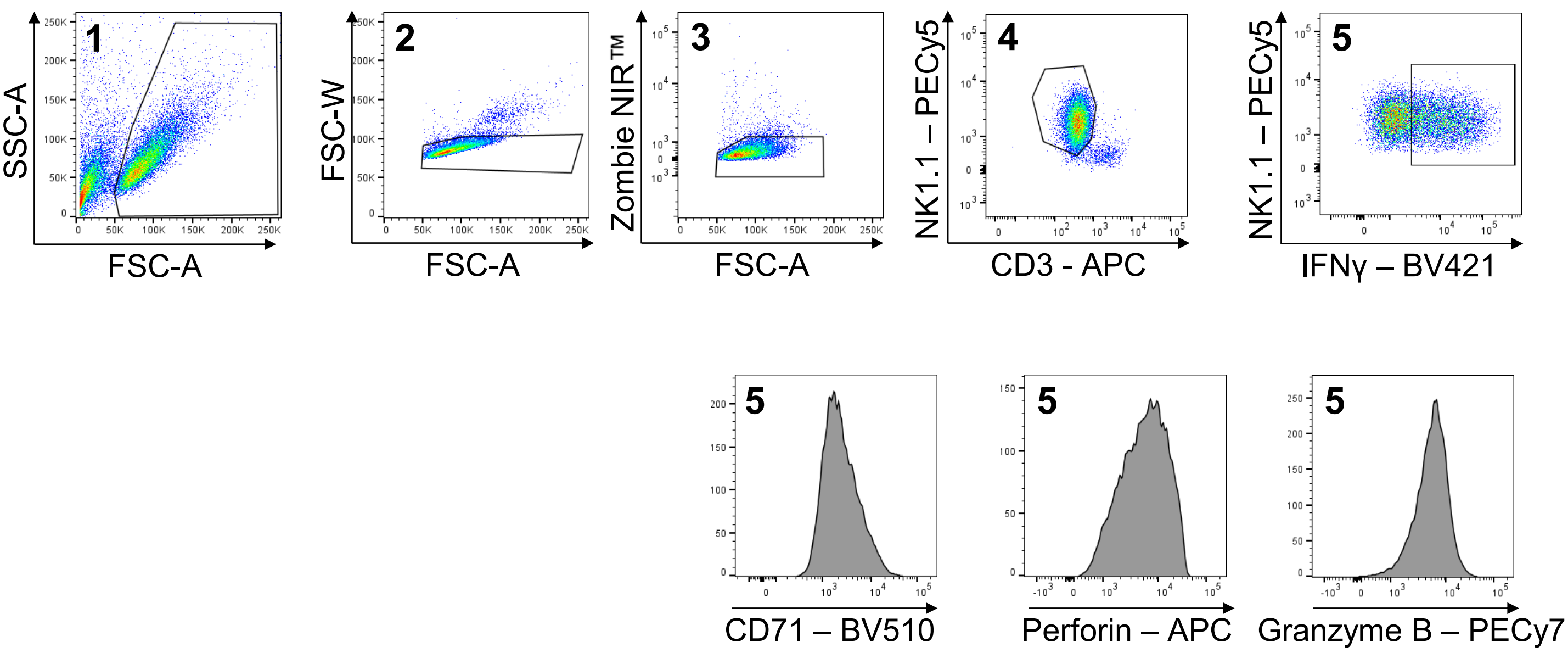

**Supplementary Table 1**

| Primer/Gene Name | Primer Sequence |
|---|---|
| **Gapdh Forward** | 5’-CATGGCCTTCCGTGTTCCTA-3’ |
| **Gapdh Reverse** | 5’-CCTGCTTCACCACCTTCTTGAT-3’ |
| **Rplp0 Forward** | 5’-CATGTCGCTCCGAGGGAAG-3’ |
| **Rplp0 Reverse** | 5’-CAGCAGCTGGCACCTTATTG-3’ |
| **Hprt Forward** | 5′-TGATCAGTCAACGGGGGACA-3′ |
| **Hprt Reverse** | 5′-TTCGAGAGGTCCTTTTCACCA-3’ |
| **Slc2a1 Forward** | 5’-GGAATCGTCGTTGGCATCCT-3’ |
| **Slc2a1 Reverse** | 5’-CGAAGCTTCTTCAGCACACTC-3’ |
| **Hk2 Forward** | 5’-TCGCCTGCTTATTCACGGAG-3’ |
| **Hk2 Reverse** | 5’-TCGCCTGCTTATTCACGGAG -3’ |
| **Ldha Forward** | 5’-CTGGGAGAACATGGCGACTC-3’ |
| **Ldha Reverse** | 5’-ATGGCCCAGGATGTGTAACC-3’ |
| **Cd36 Forward** | 5′-GGACATTGAGATTCTTTTCCTCTG-3′ |
| **CD36 Reverse** | 5′-GCAAAGGCATTGGCTGGAAGAAC -3′ |
| **Scarb1 Forward** | 5′- ACACCCGAATCCTCGCTGGAAT -3′ |
| **Scarb1 Reverse** | 5′- CCGTTGGCAAACAGAGTATCGG -3′ |
| **Scarb2 Forward** | 5′- TAGCCAACACCTCCGAAAACGC -3' |
| **Scarb2 Reverse** | 5′- CGAACTTCTCGTCGGCTTGGTA -3′ |
| **Cpt1a Forward** | 5’-GACTCCGCTCGCTCATTCC-3’ |
| **Cpt1a Reverse** | 5’-GCCATTCTTGAATCGGATGAACT-3’ |
| **Fabp5 Forward** | 5′-GACGACTGTGTTCTCTTGTAACC-3′ |
| **Fabp5 Reverse** | 5′- TGTTATCGTGCTCTCCTTCCCG-3′ |
| **Plin2 Forward** | 5'-CAGCCAACGTCCGAGATTG-3' |
| **Plin2 Reverse** | 5'-CACATCCTTCGCCCCAGTT-3' |
