# Supplementary material for: Macrophages tune NK cell IFNγ production through direct lipid transfer
Source: Nat Commun. 2026 Aug 8;17:9527. doi: 10.1038/s41467-026-76444-0 (PMC13547247; doi:10.1038/s41467-026-76444-0)
Supplement: Supplementary file 2 [file 41467_2026_76444_MOESM2_ESM.pdf]

Description of Additional Supplementary Files

Supplementary Data 1. Metabolomic analysis of DAG and TAG in NK cells

Supplementary Data 2. Metabolomics of media from cultures of BMDM +/- LPS stimulation
