# Supplementary material for: Macrophages tune NK cell IFNγ production through direct lipid transfer
Source: Nat Commun. 2026 Aug 8;17:9527. doi: 10.1038/s41467-026-76444-0 (PMC13547247; doi:10.1038/s41467-026-76444-0)
Supplement: Supplementary file 3 [file 41467_2026_76444_MOESM5_ESM.pdf]

nature portfolio

Corresponding author(s): Prof. David K Finlay

Last updated by author(s): Mar 30, 2026

Reporting Summary

Nature Portfolio wishes to improve the reproducibility of the work that we publish. This form provides structure for consistency and transparency in reporting. For further information on Nature Portfolio policies, see our Editorial Policies and the Editorial Policy Checklist.

Statistics

For all statistical analyses, confirm that the following items are present in the figure legend, table legend, main text, or Methods section.

n/a	Confirmed	
☐	☒	The exact sample size (*n*) for each experimental group/condition, given as a discrete number and unit of measurement
☐	☒	A statement on whether measurements were taken from distinct samples or whether the same sample was measured repeatedly
☐	☒	The statistical test(s) used AND whether they are one- or two-sided<br>*Only common tests should be described solely by name; describe more complex techniques in the Methods section.*
☐	☒	A description of all covariates tested
☐	☒	A description of any assumptions or corrections, such as tests of normality and adjustment for multiple comparisons
☐	☒	A full description of the statistical parameters including central tendency (e.g. means) or other basic estimates (e.g. regression coefficient) AND variation (e.g. standard deviation) or associated estimates of uncertainty (e.g. confidence intervals)
☐	☒	For null hypothesis testing, the test statistic (e.g. *F*, *t*, *r*) with confidence intervals, effect sizes, degrees of freedom and *P* value noted<br>*Give P values as exact values whenever suitable.*
☒	☐	For Bayesian analysis, information on the choice of priors and Markov chain Monte Carlo settings
☒	☐	For hierarchical and complex designs, identification of the appropriate level for tests and full reporting of outcomes
☒	☐	Estimates of effect sizes (e.g. Cohen's *d*, Pearson's *r*), indicating how they were calculated

Our web collection on statistics for biologists contains articles on many of the points above.

Software and code

Policy information about availability of computer code

Data collection	Data collection for flow cytometry experiments was performed with Becton Dickinson (BD) FACSDiva software. Data collection for real-time qPCR was performed using QuantStudio Real-time PCR software. Data collection for confocal microscopy was performed using LASX imaging software.
Data analysis	Flow cytometry data was analysed using FlowJo (TreeStar). Confocal microscopy images were analysed using ImageJ (NIH). Statistical analysis was performed using GraphPad Prism (GraphPad Software).

For manuscripts utilizing custom algorithms or software that are central to the research but not yet described in published literature, software must be made available to editors and reviewers. We strongly encourage code deposition in a community repository (e.g. GitHub). See the Nature Portfolio guidelines for submitting code & software for further information.

Data

Policy information about availability of data

All manuscripts must include a data availability statement. This statement should provide the following information, where applicable:

- Accession codes, unique identifiers, or web links for publicly available datasets
- A description of any restrictions on data availability
- For clinical datasets or third party data, please ensure that the statement adheres to our policy

The source files for metabolomics of IL2-NK cells co-cultured with LPS-stimulated BMDMs are provided as supplementary material. The source files for

metabolomics of co-culture medium of IL2-NK cell co-cultures are provided as supplementary material. All source data has been provided as source data files. All other data supporting the findings of this study are available from the corresponding author on reasonable request.

Research involving human participants, their data, or biological material

Policy information about studies with human participants or human data. See also policy information about sex, gender (identity/presentation), and sexual orientation and race, ethnicity and racism.

Reporting on sex and gender	N/A
Reporting on race, ethnicity, or other socially relevant groupings	N/A
Population characteristics	N/A
Recruitment	N/A
Ethics oversight	N/A

Note that full information on the approval of the study protocol must also be provided in the manuscript.

Field-specific reporting

Please select the one below that is the best fit for your research. If you are not sure, read the appropriate sections before making your selection.

☒ Life sciences ☐ Behavioural & social sciences ☐ Ecological, evolutionary & environmental sciences

For a reference copy of the document with all sections, see nature.com/documents/nr-reporting-summary-flat.pdf

Life sciences study design

All studies must disclose on these points even when the disclosure is negative.

Sample size	No statistical method was used to predetermine sample sizes, though all experiments were performed using at least 3 biological replicates across at least 2 independent experiments to ensure reproducibility.
Data exclusions	In Supplementary Figure 4C, there is a data point missing from one group (IL2/12-stimulated IL2-NK cells + BMDM) as the sample was lost during acquisition on the cytometer.<br>In supplementary Figure 4F, there is a data point missing from one group (IL2/12-stimulated IL2-NK cells + BMDM + LPS) as there was heavy metal contamination introduced to the sample during data acquisition.
Replication	To verify reproducibility, experiments were performed using at least 3 biological replicates across two independent experiments unless clearly stated in the figure legend. All attempts at replication were successful.
Randomization	For in vivo experiments, mice were randomly assigned to experimental groups. For in vitro experiments this was not required as samples were obtained from uniform primary cell cultures.
Blinding	The investigators were not blinded during experiments. This was not possible as individual investigators carried out the experiments and were required to be aware of the experimental groups and outcomes.

Reporting for specific materials, systems and methods

We require information from authors about some types of materials, experimental systems and methods used in many studies. Here, indicate whether each material, system or method listed is relevant to your study. If you are not sure if a list item applies to your research, read the appropriate section before selecting a response.

Materials & experimental systems

n/a	Involved in the study	
☐	☒	Antibodies
☐	☒	Eukaryotic cell lines
☒	☐	Palaeontology and archaeology
☐	☒	Animals and other organisms
☒	☐	Clinical data
☒	☐	Dual use research of concern
☒	☐	Plants

Methods

n/a	Involved in the study	
☒	☐	ChIP-seq
☐	☒	Flow cytometry
☒	☐	MRI-based neuroimaging

Antibodies

Antibodies used

1. NK1.1 (PK136, PECy5, 108716, BioLegend, 1:200)
2. NK1.1 (PK136, BV421, 108732, BioLegend, 1:200)
3. NK1.1 (PK136, BV786, 568224, BioLegend, 1:200)
4. CD3 (145-2C11, FITC, 11003185, eBioscience, 1:200)
5. CD3 (145-2C11, APC, 100312, BioLegend, 1:200)
6. CD3 (17A2, BV510, 100234, BioLegend, 1:200)
7. CD3 (17A2, BV650, 100229, BioLegend, 1:200)
8. CD19 (1D3, APC, 550992, BioLegend, 1:200)
9. CD19 (1D3, BV650, 563235, BioLegend, 1:200)
10. CD40 (3-23, FITC, 124608, Biolegend, 1:200)
11. CD49b (DX5, BV421, 563063, BD Biosciences, 1:200)
12. CD49b (DX5, PE, 553858, BD Biosciences, 1:200)
13. CD11b (M1/70, PECy7, 552850, BD Biosciences, 1:1000)
14. F4/80 (BM8, BV510, 123135, BioLegend, 1:400)
15. IFNγ (XMG1.2, BV421, 505830, BioLegend, 1:100)
16. Perforin (S16009B, APC, 154404, BioLegend, 1:800)
17. Granzyme B (NGZM, PECy7, 25889882, eBioscience, 1:100)
18. IL12p40/p70 (C15.6, V450, 561456, BD Biosciences, 1:100)
19. IL6 (MP5-20F3, APC, 504504, BioLegend, 1:100)
20. TNFα (MP6-XT22, PECy7, 25732182, Invitrogen, 1:100)
21. CD69 (H1.2F3, APCCy7, 104526, BioLegend, 1:200)
22. CD71 (C2, BV510, 563112, BD Biosciences, 1:200)
23. CD36 (CRF D-2712, PE, 562702, BD Biosciences, 1:200)

Validation

1. https://www.biolegend.com/en-gb/products/pe-cyanine5-anti-mouse-nk-1-1-antibody-3142
2. https://www.biolegend.com/en-ie/products/brilliant-violet-421-anti-mouse-nk-1-1-antibody-7150
3. https://www.biolegend.com/fr-fr/search-results/brilliant-violet-785-anti-mouse-nk-1-1-antibody-10367
4. https://www.thermofisher.com/antibody/product/CD3e-Antibody-clone-145-2C11-Monoclonal/11-0031-82
5. https://www.biolegend.com/fr-lu/products/apc-anti-mouse-cd3epsilon-antibody-21
6. https://www.biolegend.com/fr-ch/products/brilliant-violet-510-anti-mouse-cd3-antibody-7990
7. https://www.biolegend.com/nl-be/products/brilliant-violet-650-anti-mouse-cd3-antibody-7843
8. https://www.biolegend.com/en-dk/products/apc-anti-mouse-cd19-antibody-13680
9. https://www.biolegend.com/en-ie/products/brilliant-violet-650-anti-mouse-cd19-antibody-25681
10. https://www.biolegend.com/sv-se/products?GroupID=BLG5890&productid=4982
11. https://www.bdbiosciences.com/en-ie/products/reagents/flow-cytometry-reagents/research-reagents/single-color-antibodies-ruo/bv421-rat-anti-mouse-cd49b.563063?tab=product_details
12. https://www.bdbiosciences.com/en-eu/products/reagents/flow-cytometry-reagents/research-reagents/single-color-antibodies-ruo/pe-rat-anti-mouse-cd49b.553858?tab=product_details
13. https://www.bdbiosciences.com/en-ie/products/reagents/flow-cytometry-reagents/research-reagents/single-color-antibodies-ruo/pe-cy-7-rat-anti-cd11b.552850?tab=product_details
14. https://www.biolegend.com/en-gb/products/brilliant-violet-510-anti-mouse-f4-80-antibody-8934?GroupID=BLG5319
15. https://www.biolegend.com/en-ie/products/brilliant-violet-421-anti-mouse-ifn-gamma-antibody-7154
16. https://www.biolegend.com/en-ie/products/apc-anti-mouse-perforin-antibody-15281?GroupID=ImportedGROUP1
17. https://www.thermofisher.com/antibody/product/Granzyme-B-Antibody-clone-NGZB-Monoclonal/25-8898-82
18. https://www.fishersci.com/shop/products/anti-il-12-p40-p70-v450-clone-c15-6-bd/BDB561456
19. https://www.biolegend.com/en-ie/products/apc-anti-mouse-il-6-antibody-7035
20. https://www.thermofisher.com/antibody/product/TNF-alpha-Antibody-clone-MP6-XT22-Monoclonal/25-7321-82
21. https://www.biolegend.com/fr-ch/products/apc-cyanine7-anti-mouse-cd69-antibody-6986
22. https://www.bdbiosciences.com/en-br/products/reagents/flow-cytometry-reagents/research-reagents/single-color-antibodies-ruo/BV510-Rat-Anti-Mouse-CD71.563112?tab=product_details
23. https://www.bdbiosciences.com/en-eu/products/reagents/flow-cytometry-reagents/research-reagents/single-color-antibodies-ruo/pe-mouse-anti-mouse-cd36.562702?tab=product_details

Eukaryotic cell lines

Policy information about cell lines and Sex and Gender in Research

Cell line source(s)	RMA/S (murine Rauscher virus induced T cell lymphoma) cells were obtained from the lab of Prof. Elisabeth Littwitz-Salomon, University Hospital Essen. L929 cells were obtained from the lab of Prof. Luke O'Neill, Trinity College Dublin.
Authentication	Cell lines were not authenticated.
Mycoplasma contamination	Cell lines were negative for mycoplasma contamination following PCR testing (PanReac AppliChem).
Commonly misidentified lines (See ICLAC register)	No commonly misidentified cell lines were used.

Animals and other research organisms

Policy information about studies involving animals; ARRIVE guidelines recommended for reporting animal research, and Sex and Gender in Research

Laboratory animals	6-20 week old, male and female, C57BL/6 mice were obtained from Inotiv. CD36KO (B6.129S1-Cd36tm1Mfe/J) mice were purchased from Jackson Laboratories.
Wild animals	No wild animals were used in this study.
Reporting on sex	Both male and female mice were used in this study and no sex-related differences were observed or reported.
Field-collected samples	No field-collected samples were used in this study.
Ethics oversight	All mice were maintained in compliance with project license, EU, and Health Products Regulatory Authority regulations, with approval of the University of Dublin's ethical review board.

Note that full information on the approval of the study protocol must also be provided in the manuscript.

Plants

Seed stocks	N/A
Novel plant genotypes	N/A
Authentication	N/A

Flow Cytometry

Plots

Confirm that:

- [x] The axis labels state the marker and fluorochrome used (e.g. CD4-FITC).
- [ ] The axis scales are clearly visible. Include numbers along axes only for bottom left plot of group (a 'group' is an analysis of identical markers).
- [x] All plots are contour plots with outliers or pseudocolor plots.
- [x] A numerical value for number of cells or percentage (with statistics) is provided.

Methodology

Sample preparation	For ex vivo experiments, spleens were passed through a 70 µm strainer, or a peritoneal lavage was performed before staining. For in vitro experiments cells were incubated for indicated times before a sample was taken for staining. Cells were washed with FACS buffer to remove any remaining culture medium. Cells were incubated with Fc blocking antibody for 5 min at 4°C to prevent non-specific binding of FACS antibodies to the cells. Zombie™ viability dyes (Invitrogen) were used as viability stains and added to the cell suspension. Cells were then stained with saturating concentrations of antibodies specific for surface markers and incubated for 20 min at 4°C in the dark. The stained sample was then washed with FACS buffer and resuspended in FACS buffer for analysis.

Where intracellular staining was required 100 µL of cell suspension was taken 4 h before the end of the incubation and transferred to a U bottom 96 well plate. When samples were obtained directly ex vivo the cell suspension was resuspended in 100 µL cRPMI and transferred to a U bottom 96 well plate. GolgiPlug™ (0.2 µg/mL, BD Biosciences) was added to the sample and incubated for 4 h in an incubator at 37°C and 5% CO_2. Cells were surface stained, followed by fixation and permeabilisation using the Cytofix/Cytoperm kit (BD Biosciences) according to the manufacturer's instructions. Intracellular antibodies were added to the permeabilised cells at saturating concentrations for a further 30 mins at 4°C in the dark before the cells were washed with FACS buffer and analysed.

Puromycin incorporation was used as a measure of protein translation. Cells were washed from stimulation medium or directly taken ex vivo and transferred to a U bottom 96 well plate. Cells were surface stained before being washed with pre-warmed HBSS. Samples were then incubated with O-propargyl puromycin (20 µM, Jena Bioscience) in HBSS for 30 mins at 37° C. If this was performed in the presence of various cell treatments, cells were pre-treated with these treatments for 10 mins at 37°C prior to addition of O-propargyl puromycin. After incubation, incorporation was stopped by fixing cells with PFA (1%, Merck) for 15 mins at RT. Cells were washed with PBS before being permeabilised with click chemistry permeabilisation buffer for 15 mins at RT. Cells were then treated with a click chemistry mix containing copper (II) sulfate (1 mM, Merck), sodium-L-ascorbate (10 mM, Merck), tris-hydroxypropyltriazolylmethylamine (1 mM, Click Chemistry Tools), aminoguanidine hydrochloride (10 mM, Cayman Chemical) and azide fluorophore (5 µM, Jena Bioscience) for 1 h at RT. After staining, cells were washed three times with PBS and samples were analysed on FACS Canto II (BD).

Oleic acid alkyne was used as a measure of oleic acid uptake using click chemistry. Cells were washed from stimulation medium or directly taken ex vivo and transferred to a U bottom 96 well plate. Cells were washed with pre-warmed RPMI before being resuspended in RPMI containing oleic acid alkyne (6 µM, Leiden Institute of Chemistry, Leiden University, The Netherlands). Cells were incubated at 37°C for 10 mins. Sodium oleate (60 µM, Merck) was used as a competition control. Uptake was stopped by placing cells on ice and washing twice with acid wash prior to fixing with PFA (1%, Merck). Cells were washed with PBS before being permeabilised with click chemistry perm buffer for 15 mins at RT. Cells were then treated with a click chemistry mix containing copper (II) sulfate (1 mM, Merck), sodium-L-ascorbate (10 mM, Merck), tris-hydroxypropyltriazolylmethylamine (1 mM, Click Chemistry Tools), aminoguanidine hydrochloride (10 mM, Cayman Chemical) and azide fluorophore (5 µM, Jena Bioscience) for 1 h at RT. After staining, cells were washed three times with PBS and samples were analysed on FACS Canto II (BD).

Acridine Orange was used as an indicator of mitochondrial mass. Cells were washed from stimulation medium and added to a U bottom 96 well plate. Cell suspensions were then resuspended in RPMI containing Acridine Orange (100 nM, Invitrogen) for 30 mins prior to surface staining and analysis. Analysis was carried out using LSR Fortessa (BD, 488 nm laser, 530/30 BP filter for detection of MitoTracker™ Green and Acridine Orange). Tetramethyl rhodamine (TMRM) was used as an indicator of mitochondrial inner membrane polarisation. Cells were washed from stimulation medium or directly taken ex vivo and added to a U bottom 96 well plate. Cell suspensions were then resuspended in RPMI containing TMRM (100 nM, Invitrogen) for 30 mins prior to surface staining and analysis. Oligomycin (2 µM, Merck) and FCCP (20 µM, Merck) were used as positive and negative controls respectively. Analysis was carried out using LSR Fortessa (BD, 561 nm laser, 582/15 BP filter for detection of TMRM).

Fluorescently labelled holotransferrin was used to assess capacity for transferrin uptake by cells. Cells were washed from stimulation medium or directly taken ex vivo with transferrin uptake buffer. Cells were then incubated with transferrin uptake buffer at 37°C and 5% CO_2. Cells were washed with transferrin uptake buffer and resuspended in transferrin uptake buffer containing AlexaFluor647-conjugated holotransferrin (5µg/mL, Invitrogen) for 10 mins at 37°C. Uptake was stopped by placing cells on ice and washing twice with acid wash prior to surface staining and analysis. Unlabelled holotransferrin (500 µg/mL, Merck) was used as a competition control.

LipidTOX™ Green was used as an indicator of neutral lipid content of cells. Cells were washed from stimulation medium or directly taken ex vivo and added to a U bottom 96 well plate. Cells were washed from stimulation media and surface stained in the presence of LipidTOX™ Green (1X, Invitrogen) before being washed with FACS buffer and analysed using FACS Canto II (BD, 488 nm laser, 530/30 BP filter for detection of LipidTOX™ Green).

Instrument

Cells were analysed using FACS Canto II (BD) and LSR Fortessa (BD).

Software

Flow cytometry data was collected using FACSDiva (BD) and analysed using FlowJo (TreeStar).

Cell population abundance

Flow cytometry was used for quantification analysis only, no post-sorting fractions were collected.

Gating strategy

All gating strategies are included as a supplementary file.

☒ Tick this box to confirm that a figure exemplifying the gating strategy is provided in the Supplementary Information.
