# Supplementary material for: Macrophages tune NK cell IFNγ production through direct lipid transfer
Source: Nat Commun. 2026 Aug 8;17:9527. doi: 10.1038/s41467-026-76444-0 (PMC13547247; doi:10.1038/s41467-026-76444-0)
Supplement: Supplementary file 4 [file 41467_2026_76444_MOESM6_ESM.pdf]

nature portfolio

# Peer Review File

# Macrophages Tune NK Cell IFNγ Production Through Direct Lipid Transfer

Corresponding Author: Dr David Finlay

**This file contains all reviewer reports in order by version, followed by all author rebuttals in order by version.**

Version 0:

Reviewer comments:

Reviewer #1

(Remarks to the Author)
This is a very interesting study from an excellent research team on the interaction between macrophages and NK cells in mice. The major finding suggests that activated macrophages can substantially downregulate IFN-gamma production in NK cells via direct transfer of fatty acids. The experiments are well done and the data is mostly nicely presented.
Major comments:
1. The entire premise is set up by an experiment injecting LPS i.p. and assuming that LPS cannot directly activate NK cells "as they lack TLR expression", presumably TLR4 in particular. Published data suggests that NK cells do express TLRs including TLR4. The authors need to show some evidence that they are not directly activating NK cells in vivo by confirming that NK cells do not express TLRs. They have also not referenced any previous studies.
2. It is not clear to me why Figure 1A is presented in this format unlike Fig.1B, C, D and F.
3. There are some nice experiments with inhibitors that indirectly suggest fatty acids are transferred directly to NK cells leading to an accumulation. Why is it not possible to label the fatty acids synthesised in macrophages so that after co-culture they can be detected in NK cells to give definitive proof of cell-to-cell transfer

Reviewer #2

(Remarks to the Author)
This is an interesting and very good manuscript investigating the impact of LPS on the interaction of macrophages and NK cells and subsequent activation of the NK cells. The data presented largely supports the hypothesis that LPS alters lipid metabolism within macrophages and this leads to an increase in intracellular lipid droplets. Accumulation of these lipid droplets correlates with decreased production of pro-inflammatory cytokines by the macrophages, including IL-6, IL-12, along with CD40.

Long chain fatty acids within lipid droplets are subsequently transferred to co-cultured NK cells, which alters their metabolism and increases production of several effector molecules (perforin, granzyme B, IL-12), while inhibiting IFNγ. Interestingly, these changes do not appear to impact cytolytic functions of the NK cells.

When in vitro co-cultured with LPS-activated BMDC or splenic macrophages, NK cells had increased protein synthesis, mitochondrial mass, CD71 and CD36 levels. There is also an increase in intracellular lipid droplets. When lipid oxidation is inhibited pharmacologically, protein synthesis is decreased, but there is no significant change in effector molecules (IFNγ, granzyme B, TNFα). This is interpreted as due to metabolic changes within the NK cells.

They showed that the CD36 is acquired by the NK cells from the macrophages via trogocytosis in an actin-dependent mechanism. Surprisingly, the CD36 is not necessary for lipid accumulation within the NK cell.

The lipid accumulation in the NK cell is not due to de novo synthesis within the NK cell, but appears to be dependent upon de novo synthesis within the macrophages. Pre-treatment of macrophages with the pharmacological FASN inhibitor, C75, decreased macrophage expression of proinflammatory cytokines, CD40 expression, and blocked accumulation of lipid droplet deposition. NK cells interacting with these treated macrophages have increased IFNγ expression, in contrast to the inhibition seen with LPS-treated macrophages with intact lipid metabolism.

I appreciated the thorough discussion of potential mechanisms of the intercellular lipid transfer. The authors propose several mechanisms including trogocytosis, but there are limited examples of cytoplasmic transfer during trogocytosis. The second model involves the appearance of these lipids on the outer leaflet on the macrophage and subsequent uptake by the NK cell. The intercellular transfer mechanism is a very intriguing question, as it is in line with the initial 2003 trogocytosis paper from Joly and Hudrisier that suggested that trogocytosis may be a way for effector cells to capture metabolic products, specifically lipids.

The data is well presented and clearly support the conclusions. There is one question that needs to be addressed by the authors. What is the physiological relevance of these lipid droplet-associated gene expression differences if they don't alter the effector functions they assessed? Did they test the cytolytic ability of the in vivo-generated NK cells? The in vitro co-culture data show that there are changes to several molecules in the NK cells (perforin, granzyme B, IL-12, IFNγ), but these changes don't change their cytolytic capacity. This calls into question whether this lipid transfer actually inhibits the NK cells. The manuscript would be improved with effector data from the in vivo-generated NK cells, but additional clarification from the authors in the discussion would suffice.

Reviewer #3

(Remarks to the Author)
The study demonstrates that activated macrophages can transfer newly synthesized fatty acids to NK cells via direct cell–cell contact, leading to the suppression of IFN-γ production. However, several critical aspects of this mechanism warrant further investigation.
1. The mechanism underlying lipid transfer remains incompletely understood. Although the study proposes that CD36 is transferred to NK cells through trogocytosis, it reports that CD36 does not influence lipid accumulation in recipient NK cells. This raises questions about the actual pathway of fatty acid transfer-whether through extracellular vesicles (e.g., exosomes), membrane nanotubes, or direct membrane fusion-and the functional role of CD36 in this process. To address these uncertainties, live-cell imaging combined with super-resolution microscopy should be employed to dynamically visualize and track the cellular events associated with lipid transfer.
2. The specific classes of fatty acids being transferred have not been definitively identified. A comprehensive lipidomic profiling approach is recommended to characterize the molecular composition of the transferred lipids from macrophages to NK cells.
3. The assessment of NK cell function remains incomplete. Although IFN-γ production is significantly impaired, the effects on cytotoxic function-assessed via perforin and granzyme B expression-show inconsistent changes across experimental models, with no observed alteration in overall cytotoxicity. This apparent "functional decoupling" between cytokine production and cytolytic activity necessitates mechanistic explanation. Furthermore, the impact of lipid accumulation on NK cell viability, proliferation, and differentiation has not been evaluated. It is therefore essential to systematically examine markers of NK cell exhaustion, proliferative capacity, and the expression profiles of activating and inhibitory surface receptors.
4. The downstream mechanisms by which intracellular lipid accumulation suppresses IFN-γ production in NK cells remain unclear. Specifically, it is unknown whether accumulated lipids or lipid droplets interfere with key metabolic signaling pathways (e.g., mTOR, AMPK), mitochondrial bioenergetics, transcriptional regulation (e.g., STAT4 activation), or secretory machinery. To identify potential regulatory nodes, integrated omics analyses, including transcriptomics, metabolomics, or phosphoproteomics, should be performed on NK cells following co-culture with macrophages.
5. The rationale for the selected co-culture duration requires clarification. Given that the duration of macrophage–NK cell interactions in vivo may be considerably shorter than those used in prolonged co-culture experiments, it is important to assess whether the observed phenotypic and functional changes reflect physiological conditions or are artifacts of extended in vitro exposure
6. While pharmacological inhibition using C75 was employed to block fatty acid synthesis, genetic validation is lacking. The use of macrophage-specific fatty acid synthase (FASN) conditional knockout models would provide more definitive evidence supporting the role of de novo lipogenesis in mediating immunosuppressive effects on NK cells.

Version 1:

Reviewer comments:

Reviewer #2

(Remarks to the Author)
The revisions to the manuscript significantly improve it and alleviate prior concerns. Thank you to the authors for critically evaluating and responding thoughtfully to the prior critique. I recommend publishing this revised manuscript.

I have been asked to review the responses to the comments from reviewer #1. In my opinion, they adequately addressed their concerns and the paper should be published.

Reviewer #3

(Remarks to the Author)

We greatly appreciate your comprehensive responses and supplementary explanations to our inquiries. Nearly all questions have been addressed with sufficient detail, and the challenges associated with unfeasible experiments have also been discussed.

minor revision (If feasible):

1.Future studies could further validate the downstream mechanisms by which intracellular lipid accumulation inhibits IFN-γ production in NK cells. Previous research has shown that upon uptake of lipids from macrophages, NK cells exhibit reduced mTORC1 activity, which ultimately suppresses IFN-γ production. Whether the inhibition of IFN secretion induced by lipid accumulation in NK cells can be reversed via mTORC1 activation warrants investigation.

2. The article proposes that NK cells and macrophages form sustained interactions, and such direct crosstalk indeed plays a critical role in regulating NK cell function. Determining the optimal contact duration between NK cells and macrophages via dynamic monitoring may enhance the scientific rigor of experimental design.

**Open Access** This Peer Review File is licensed under a Creative Commons Attribution 4.0 International License, which permits use, sharing, adaptation, distribution and reproduction in any medium or format, as long as you give appropriate credit to the original author(s) and the source, provide a link to the Creative Commons license, and indicate if changes were made.
In cases where reviewers are anonymous, credit should be given to 'Anonymous Referee' and the source.
The images or other third party material in this Peer Review File are included in the article's Creative Commons license, unless indicated otherwise in a credit line to the material. If material is not included in the article's Creative Commons license and your intended use is not permitted by statutory regulation or exceeds the permitted use, you will need to obtain permission directly from the copyright holder.
To view a copy of this license, visit https://creativecommons.org/licenses/by/4.0/

23rd March 2026

Dear Expert reviewers

Thank you for taking the time to read and critically assess our manuscript. We greatly appreciate the insightful comments and constructive suggestions provided by the reviewers. These have been invaluable in strengthening the clarity and depth of our study, and the additional experiments performed during revision have meaningfully enhanced the overall story. Below, we provide a detailed, point-by-point response (in red) to each of the reviewers' comments.

**Reviewer #1** (Remarks to the Author) – Expert on NK cells and metabolism

This is a very interesting study from an excellent research team on the interaction between macrophages and NK cells in mice. The major finding suggests that activated macrophages can substantially downregulate IFN-gamma production in NK cells via direct transfer of fatty acids. The experiments are well done and the data is mostly nicely presented.

Major comments:

1. The entire premise is set up by an experiment injecting LPS i.p. and assuming that LPS cannot directly activate NK cells "as they lack TLR expression", presumably TLR4 in particular. Published data suggests that NK cells do express TLRs including TLR4. The authors need to show some evidence that they are not directly activating NK cells in vivo by confirming that NK cells do not express TLRs. They have also not referenced any previous studies.

We thank the reviewer for raising this important point. We agree that the literature on TLR4 expression in NK cells is heterogeneous, varies by species, and is further complicated by the distinction between intracellular and surface localisation. Several studies report that human NK cells can express low or context-dependent intracellular TLR4 and may respond to LPS under specific activating conditions (e.g., cytokine co-stimulation)(Souza-Fonseca-Guimaraes, Parlato et al. 2012, Kanevskiy, Erokhina et al. 2014, Kanevskiy, Erokhina et al. 2019). In contrast, a detailed comparison of full LPS and re-LPS stimulation demonstrated that human

NK cells express little to no surface TLR4, and that their responses do not follow canonical TLR4-dependent signalling pathways; the authors concluded that NK cells “do not express or express very low level of surface TLR4” (Kanevskiy, Erokhina et al. 2019)

To address this issue directly in our system, we examined Tlr4 expression in murine NK cells using quantitative proteomics. We have generated two independent ex vivo proteomic datasets from naïve splenic NK cells and in vivo-activated NK cells (18 h post-poly(I:C)). These datasets quantify 5,654 and 5,137 proteins, respectively, and detect plasma membrane proteins at expected copy numbers. Tlr4 is not detected in either dataset, whereas low-abundance membrane proteins such as the amino-acid transporter Slc7a1 (~50 copies per cell) are readily quantified. We have also analysed IL-15-expanded splenic NK cells (data public at www.immpres.co.uk). In these cells, Tlr4 is detected at ~300 copies per cell, but only on the basis of a single unique peptide, and therefore this represents a low-confidence, near-limit-of-detection call. In contrast, the same proteomic pipeline robustly quantifies Tlr4 in macrophages, eosinophils, and mast cells, where it is supported by 3-5 unique peptides, confirming that the workflow is capable of detecting Tlr4 where it is genuinely expressed.

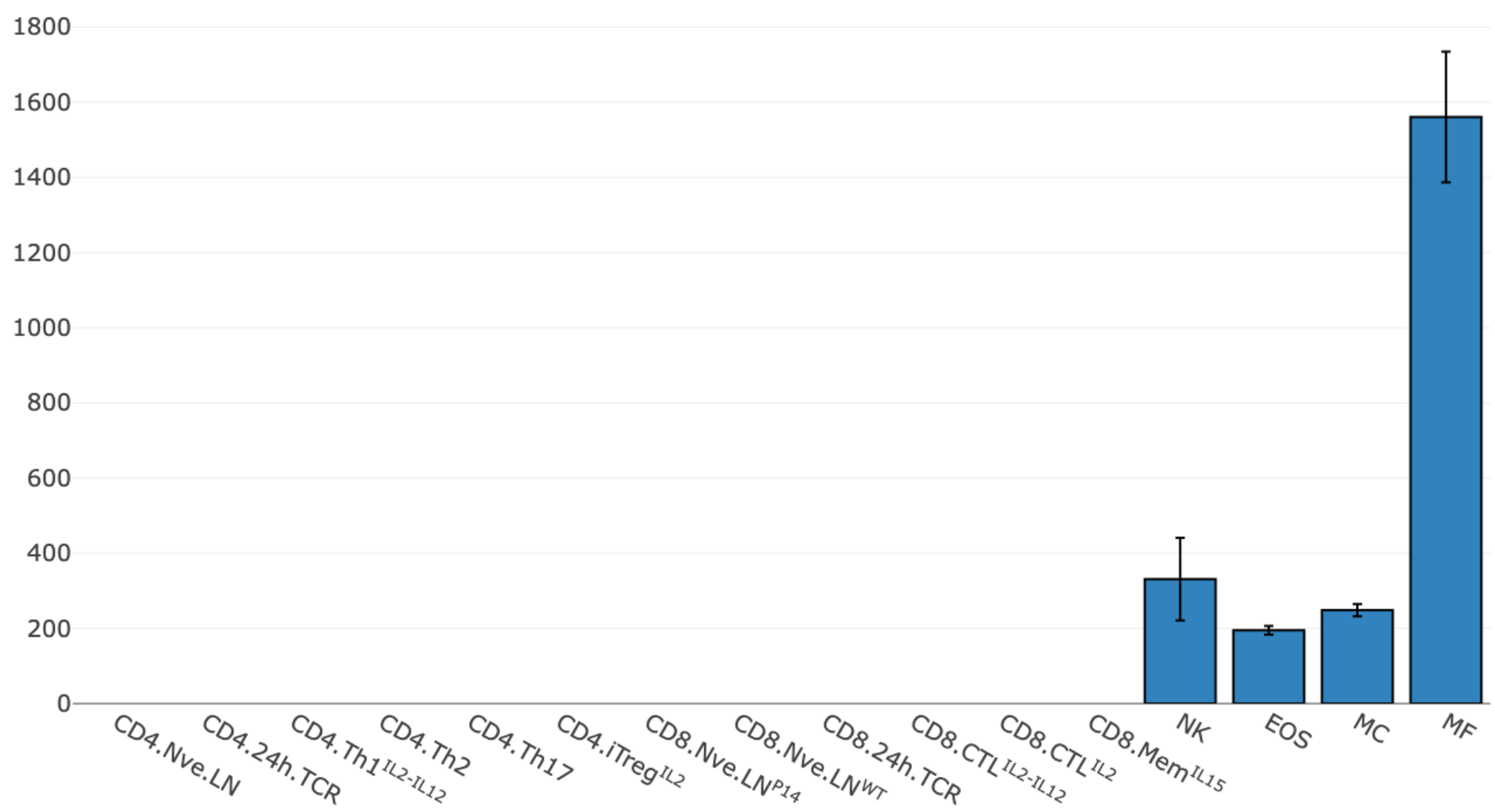


Taken together, these results demonstrate that murine NK cells directly ex vivo express no detectable Tlr4, or levels below the reliable limit of detection, as assessed by a sensitive and validated quantitative proteomics approach. Low-level Tlr4 may be detectable under specific in vitro expansion conditions

(e.g., IL-15), but this signal is weak and not robust. Consistent with this, splenic NK cells in our study were unresponsive to LPS stimulation, supporting the conclusion that TLR4-mediated direct activation does not occur under our experimental conditions. (See Figure 1A, Supplementary Figure 1)

2. It is not clear to me why Figure 1A is presented in this format unlike Fig.1B, C, D and F.

We thank the reviewer for this helpful observation. Figure 1A was originally presented as a radar plot to provide a simple visual summary, as we considered it primarily a control demonstrating that NK cells do not respond directly to LPS. However, we now appreciate that, given the ongoing debate in the literature regarding NK-cell responsiveness to LPS, it is important for readers to see the full dataset and its inherent variability. In response to the reviewer's comment, we have therefore added the complete underlying data as Supplementary Figure 1 (see also below), presented in a format consistent with the other panels in Figure 1.

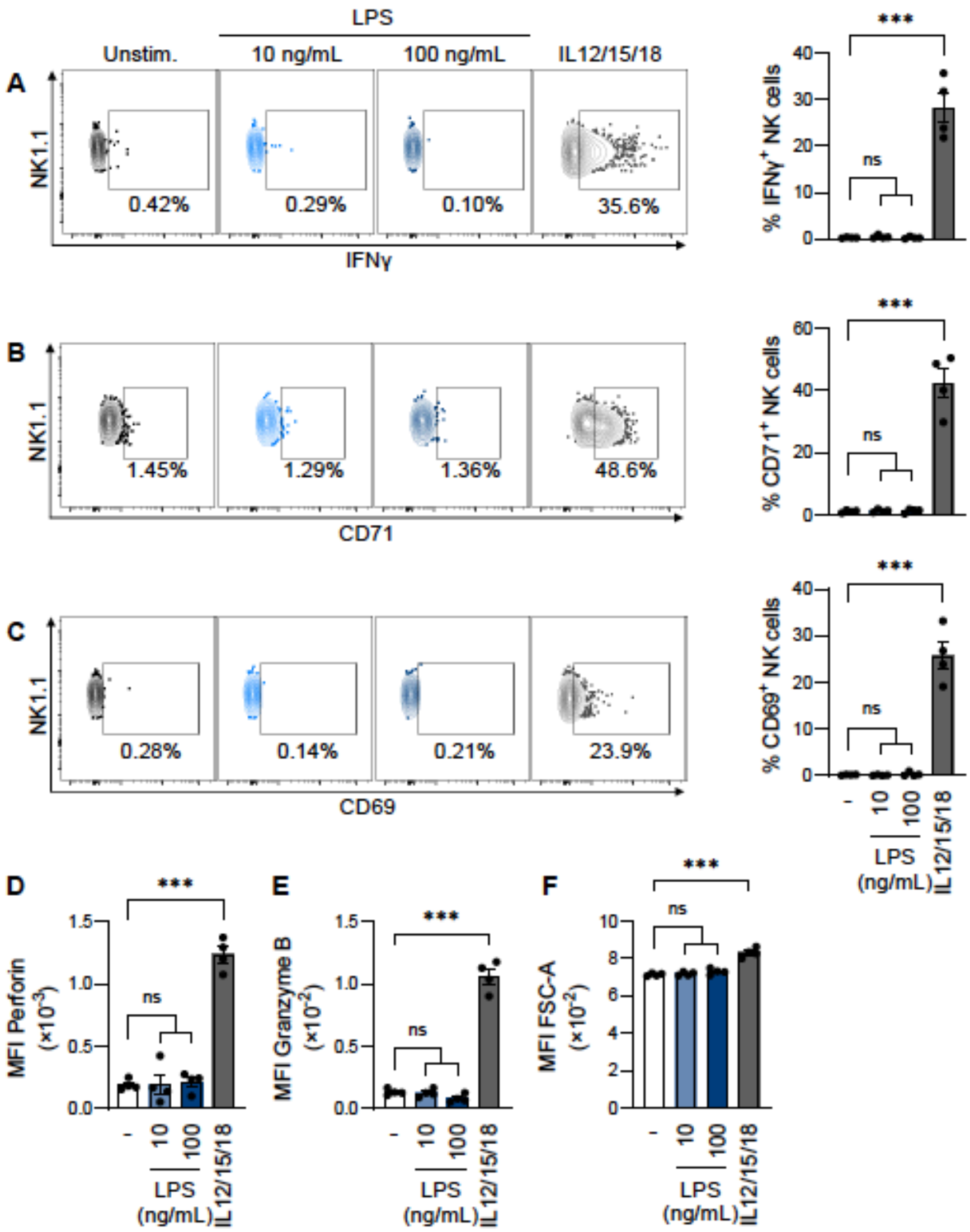


3. There are some nice experiments with inhibitors that indirectly suggest fatty acids are transferred directly to NK cells leading to an accumulation. Why is it not possible to label the fatty acids synthesised in macrophages so that after co-culture they can be detected in NK cells to give definitive proof of cell-to-cell transfer.

We appreciate the reviewer's suggestion and fully agree that direct labelling of macrophage-derived fatty acids would be an ideal approach. We extensively explored multiple strategies prior to the original submission and again after receiving this comment. Unfortunately, each available method presents technical limitations that prevent definitive labelling of newly synthesised fatty acids, the pool we show is transferred to NK cells.

**1) $^{13}$C-glucose labelling is not feasible for this system**. $^{13}$C-glucose would only label fatty acids made before co-culture begins. However, our data argued that that the transferred lipids are those synthesised during NK-macrophage interaction, not beforehand. Hence, we did not try this experiment. To test this directly, we inhibited fatty acid synthesis in BMDMs for the 6 h prior to co-culture using TOFA (a reversible inhibitor of fatty acid synthesis), washed the cells, and then co-cultured them with NK cells under conditions where both cell types could synthesise lipids normally. **Result**: NK cells accumulated neutral lipids to the same extent whether co-cultured with TOFA-pretreated or untreated BMDMs. This demonstrates that fatty acids made prior to co-culture (the only pool amenable to $^{13}$C labelling) do not significantly contribute to the lipid accumulation in NK cells. Thus, there is no viable window in which $^{13}$C-glucose would selectively label the relevant fatty acid pool.

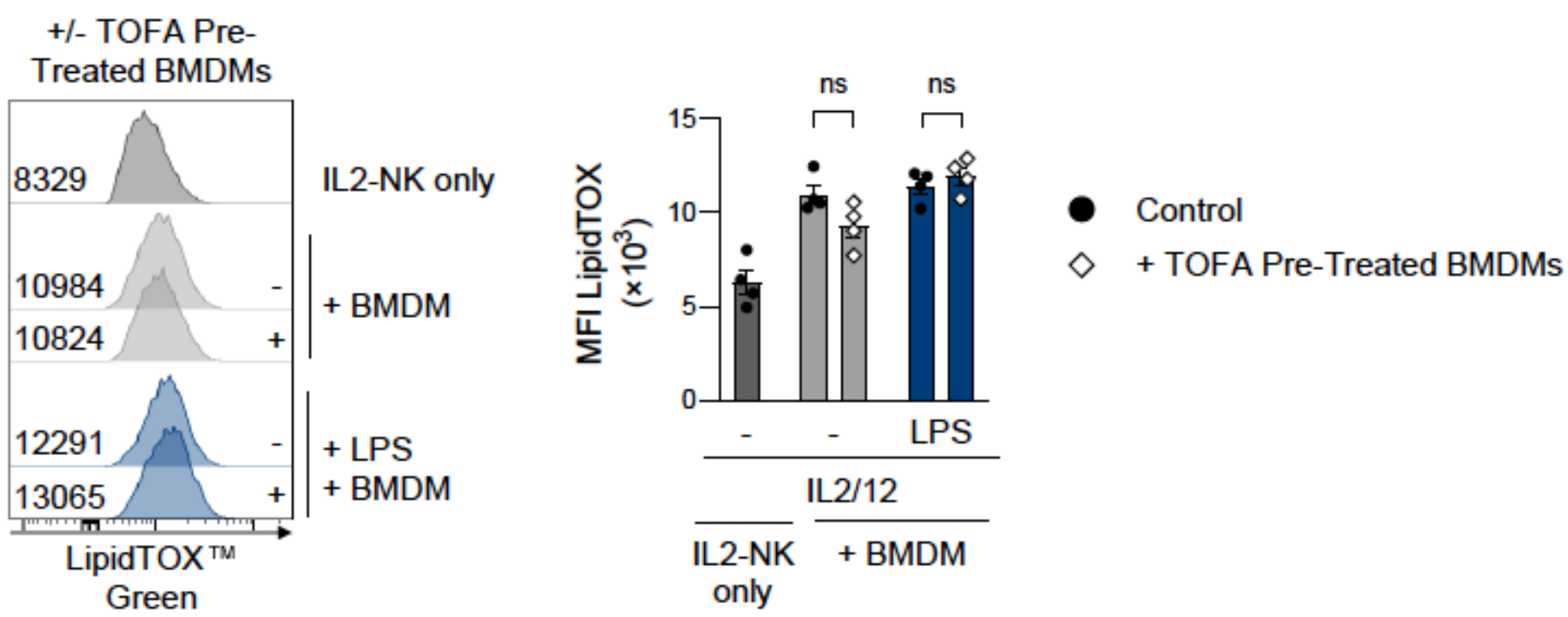


**2. Membrane dye approaches lack specificity.** We explored several lipophilic membrane dyes to track lipid transfer. However, all tested dyes exhibited significant back-exchange between cells and culture media, leading to non-specific staining and precluding reliable interpretation. For instance, media conditioned with BMDM that had been stained with these dyes was in itself sufficient to stain NK cells.

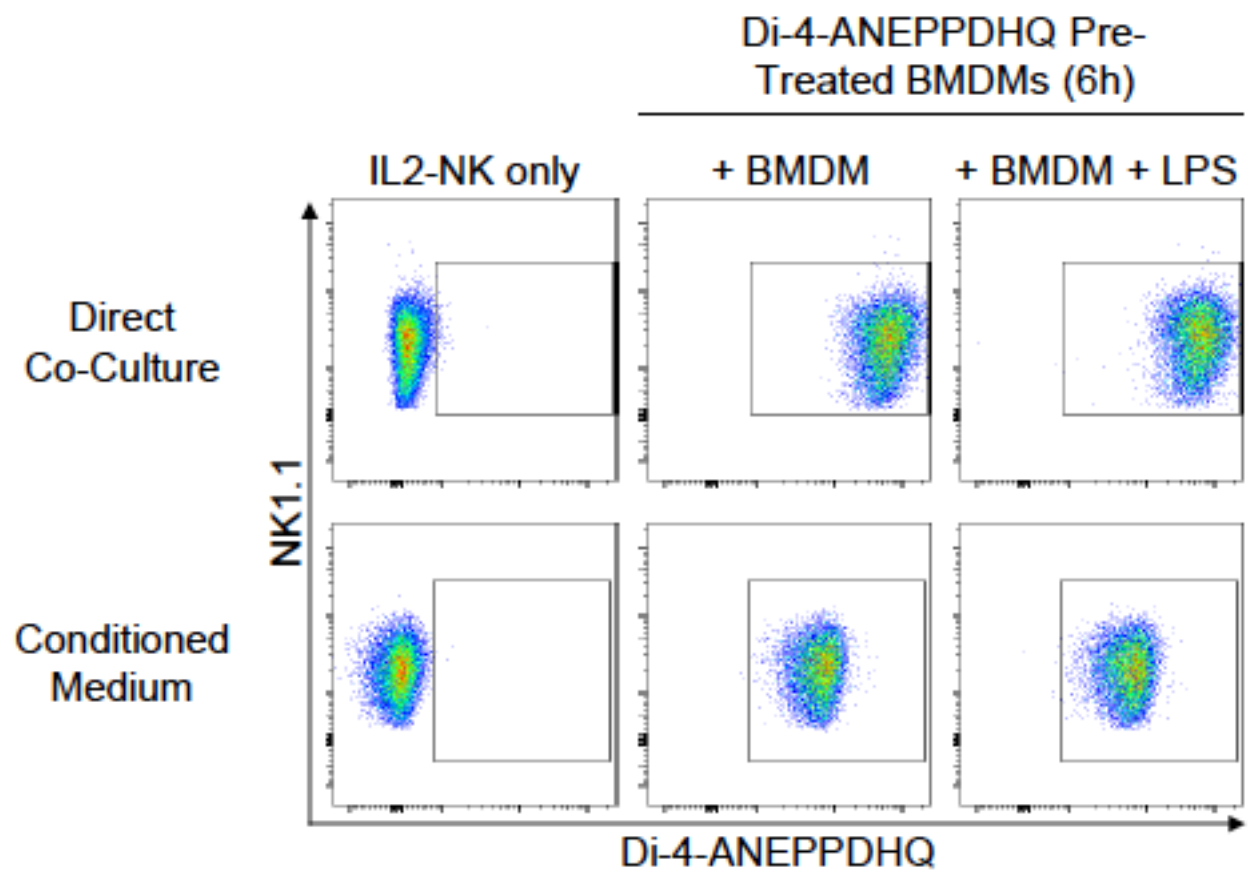


**3. Bioorthogonal fatty acid probes were ineffective.** We have a lot of experience with measuring fatty acid uptake into immune cells using click-FAs that include a bioorthogonal handle that can be used to attach a fluorophore using bioorthogonal chemistry. We attempted to preload BMDMs with alkyne-oleic acid to enable bioorthogonal tagging of transferred phospholipids (the lipid class we propose is transferred via trogocytosis or other contact dependent mechanism). Despite extensive optimisation, NK cells displayed no detectable labelled signal following co-culture. Given our findings that transferred lipids are newly synthesised, rather than scavenged from the environment, this negative result is consistent with the biology: externally supplid alkyne-oleic acid does not label fatty acids produced *de novo* during co-culture.

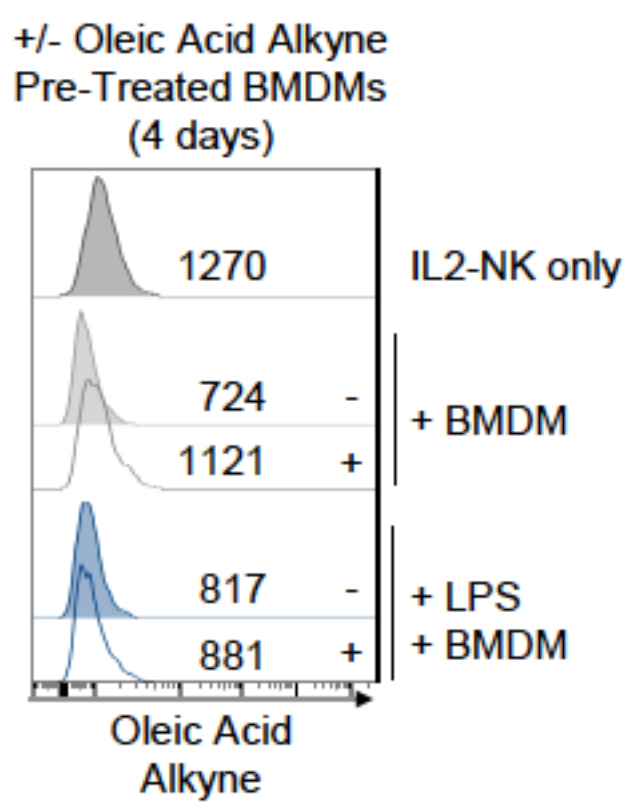

**Conclusion**

We fully agree that direct fatty-acid tracing would be ideal; however, the available chemical and metabolic labelling approaches cannot selectively label *de novo* fatty acids synthesised during co-culture, the specific pool that our data show is transferred to NK cells. For this reason, we rely on complementary inhibitor-based approaches, which robustly support the conclusion that macrophage-derived fatty acids are transferred directly to NK cells.

We now add a further discussion about the mechanisms that might be involved in the transfer of FA via phospholipids. Several studies demonstrate that macrophages spatially compartmentalise their plasma membrane to create zones enriched in newly synthesised phospholipids. The literature also supports focal exocytosis of endosomes to specific sites in the macrophage membrane, such as delivering membrane and phospholipids for phagocytosis (Wright and Silverstein 1984, Cox, Lee et al. 2000, Di, Nelson et al. 2003, Gaus, Rodriguez et al. 2005).

Reviewer #2 (Remarks to the Author) – Expert on trogocytosis

I appreciated the thorough discussion of potential mechanisms of the intercellular lipid transfer. The authors propose several mechanisms including trogocytosis, but there are limited examples of cytoplasmic transfer during trogocytosis. The second model involves the appearance of these lipids on the outer leaflet on the macrophage and subsequent uptake by the NK cell. The intercellular transfer mechanism is a very intriguing question, as it is in line with the initial 2003 trogocytosis paper from Joly and Hudrisier that suggested that trogocytosis may be a way for effector cells to capture metabolic products, specifically lipids.

We thank the reviewer for their thoughtful comments on the potential mechanisms of intercellular lipid transfer. We agree that trogocytosis has historically been associated primarily with membrane and protein transfer, and that it is very unlikely that cytoplasmic material such as lipid droplets are exchanged directly. But as the reviewer notes, (Joly and Hudrisier 2003) proposed

that trogocytosis may serve as a means for immune cells to acquire metabolic products by capturing membrane and extracting lipids and LCFA, the most energy dense fuel.

In light of our new lipidomics data :

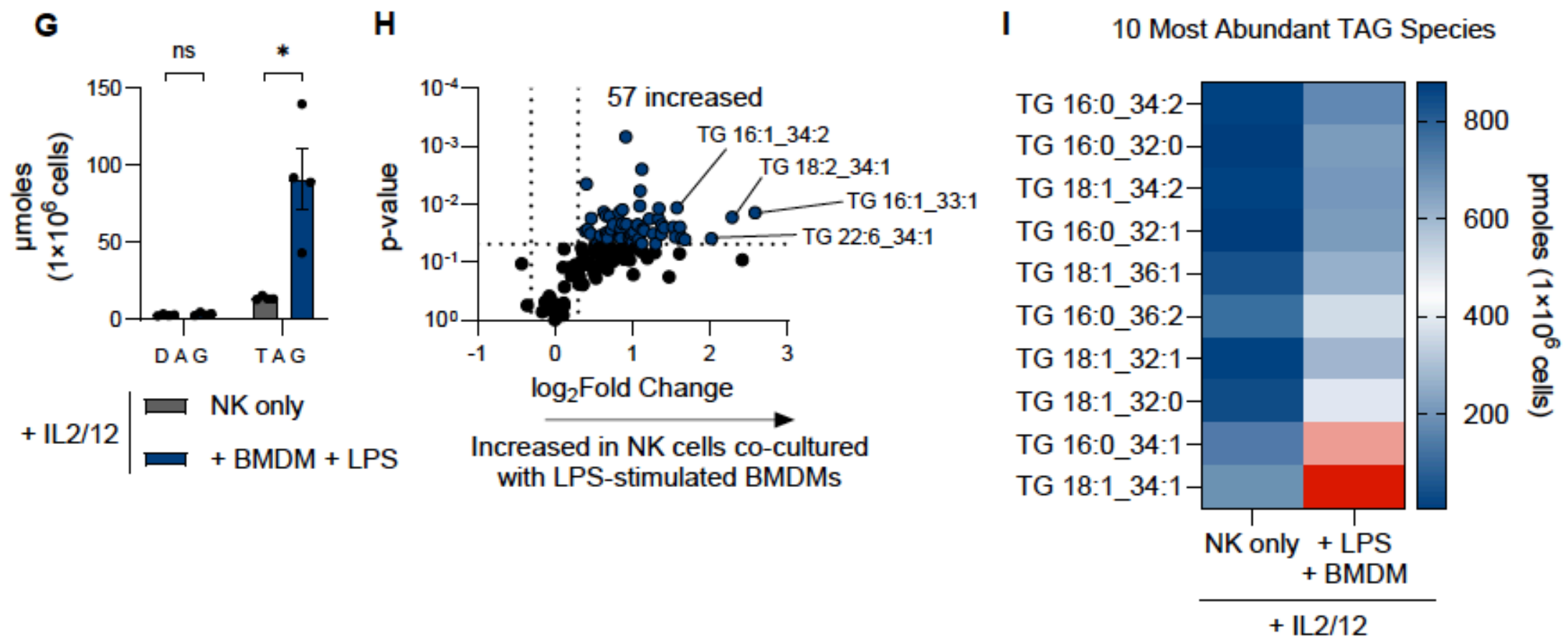


we have expanded the Discussion to further clarify the plausibility of this mechanism. Our analyses show that the increase in neutral lipids in NK cells is driven almost exclusively by triacylglycerols (TAGs) and the most dominant species are composed of palmitic acid (PA) and oleic acid (OA). These fatty acids correspond precisely to the products of de novo fatty-acid synthesis (PA) and subsequent desaturation by SCD1 (OA). The striking dominance of PA/OA-containing TAGs argues for a direct and specific transfer of newly synthesised fatty acid containing lipid species (probably phospholipids) from macrophages to NK cells. Where NK cells acquire mixed phospholipid species typical of plasma-membrane composition, the predicted FA composition of TAGs would be quite different to our new metabolomics data. If NK cells were acquiring bulk membrane fragments, we would expect a broader distribution of chain lengths and degrees of saturation reflecting the endogenous macrophage phospholipid landscape.

There is substantial support in the macrophage literature for the idea that newly synthesised phospholipids can be delivered to discrete, functionally specialised membrane domains. During phagocytosis, macrophages undergo focused exocytosis of endosomal membranes enriched in nascent phospholipids, enabling rapid expansion of the phagocytic cup (Bajno, Peng et al. 2000, Cox, Lee et al. 2000, Di, Nelson et al. 2003, Gaus, Rodriguez et al. 2005) . Similar mechanisms operate at immune synapses, where targeted exocytosis delivers

phospholipids originating from the ER or recycling endosomes to tightly regulated regions of the plasma membrane (Wright and Silverstein 1984, Gaus, Rodriguez et al. 2005).
This body of work supports the model that macrophages can create localised membrane domains enriched in newly synthesised lipids, which may provide a favourable interface for direct lipid capture by NK cells, through trogocytosis or a related contact-dependent process. We now emphasise this in the revised Discussion. We have added this text:

The literature describes a process in macrophages known as focal exocytosis, in which newly synthesised or recycled phospholipids are targeted to discrete regions of the plasma membrane(Freeman and Grinstein 2014). During this process, endosomes are trafficked to concentrated membrane sites, such as the phagocytic cup or points of cell-cell contact, where they deliver membrane lipids and proteins(Bajno, Peng et al. 2000, Di, Nelson et al. 2003). This targeted delivery provides a route to supply newly synthesised FA incorporated into phospholipids to the point of contact with NK cells. The metabolomic dataset shows that in NK cell TAGs are predominantly made up of palmitic and oleic acid FA chains, a strong argument that NK cells are extracting lipids from one of these discrete regions, the target of focal exocytosis for endosomes from the ER. The alternative, were NK cells acquiring phospholipid species from typical of plasma-membranes, we would expect a broader distribution of FA chain lengths and degrees of saturation reflecting the endogenous macrophage phospholipid landscape.

The data is well presented and clearly support the conclusions. There is one question that needs to be addressed by the authors.

What is the physiological relevance of these lipid droplet-associated gene expression differences if they don't alter the effector functions they assessed?

While the cytotoxic activity of NK cells against tumour cells and virus-infected targets is the most widely recognised aspect of NK cell biology, the importance of NK-cell-derived IFN-γ should not be underestimated. IFN-γ represents a crucial effector function of NK cells, profoundly influencing macrophage activation and T cell responses across diverse pathological contexts. Its impact has been demonstrated in settings ranging from infectious disease to tumour immunity and even tissue repair processes, including those in the liver (Scharton and Scott 1993, Biron, Nguyen et al. 1999, Vivier, Raulet et al. 2011, Li, Zeng et al. 2015, Lin, Rong et al. 2021).

We have added further data to show the changes in the IFNγ production that occur due to the interactions with macrophages. A time course analysis of Ifng mRNA (Figure 3C) and secreted IFNγ protein (Figure 3D) shows an early peak and subsequent decline when NK cells interact with inflammatory LPS stimulated macrophages. With unstimulated BMDM or NK cells stimulated alone there is a more gradual and sustained production of IFNγ cytokine.

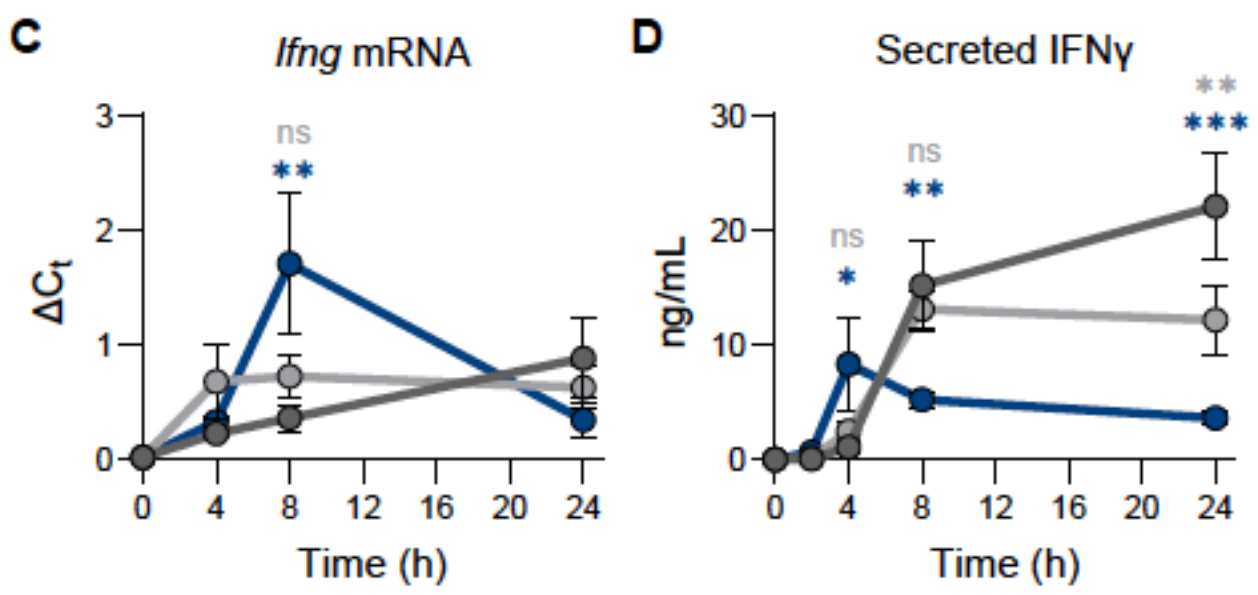


Did they test the cytolytic ability of the in vivo-generated NK cells?

We did not assess the cytolytic activity of NK cells isolated directly from LPS-treated mice. As our data developed, it became clear that the pathway we describe primarily regulates NK-cell IFN-γ-dependent inflammatory function, rather than cytotoxicity. This focus was further supported by the lack of change in killing capacity in our *in vitro* co-culture system despite measurable reductions in granzyme B and perforin. For this reason, and because our mechanistic findings consistently pointed to selective modulation of cytokine production, we did not pursue *in vivo* cytotoxicity assays.

The in vitro co-culture data show that there are changes to several molecules in the NK cells (perforin, granzyme B, IL-12, IFNγ), but these changes don't change

their cytolytic capacity. This calls into question whether this lipid transfer actually inhibits the NK cells.

We appreciate the reviewer's point. While we observe reductions in granzyme B and perforin following co-culture, it is important to note that these cytotoxic effector molecules are extremely abundant in NK cells. Consequently, a modest decrease in their expression does not necessarily impair cytolytic activity. To clarify this, we have now included FMO controls to illustrate the absolute amount of granzyme B and perforin that remains in NK cells despite the observed reductions. These data show that NK cells retain high intracellular stores of both molecules, consistent with the unchanged cytotoxic capacity observed in functional assays.

**Supplementary Figure 3B,C: IL2-NK – BMDM coculture**

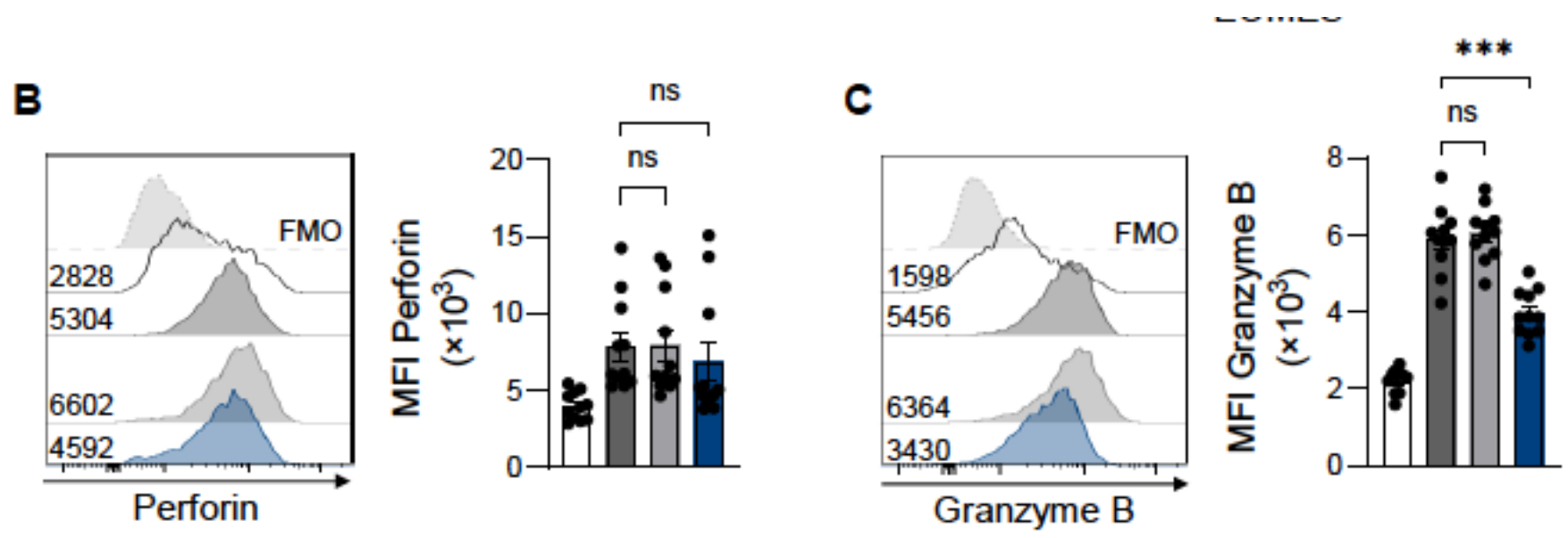


**Supplementary Figure 3E,F: spNK – Peritoneal macrophage coculture**

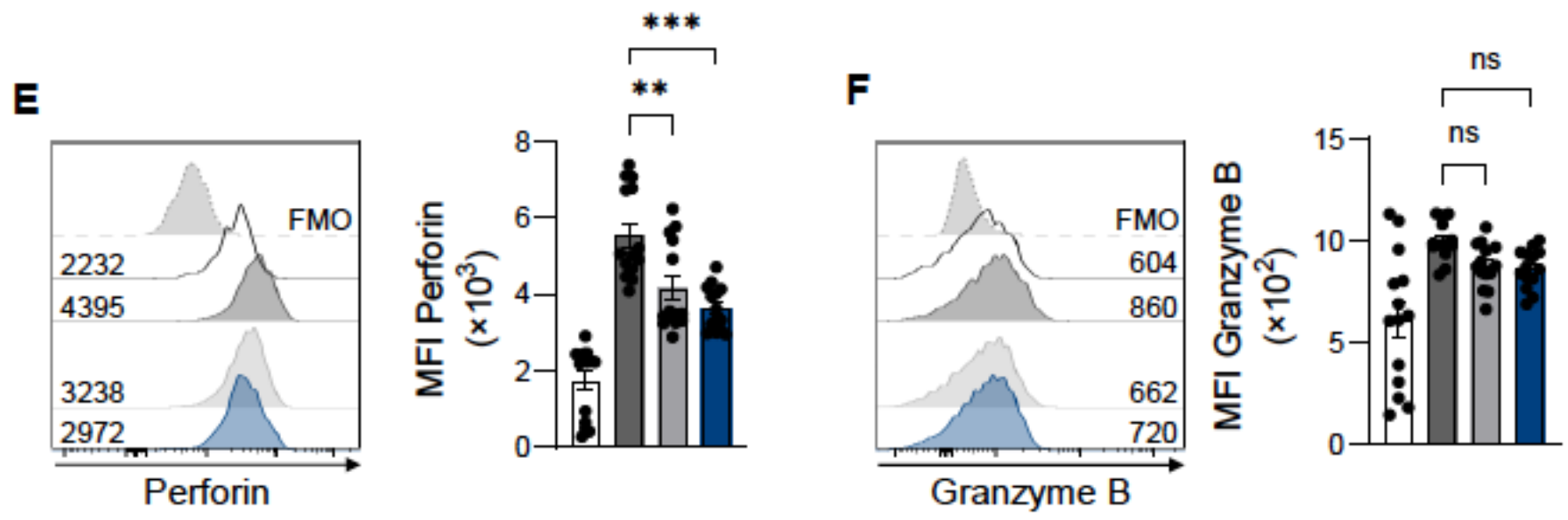


The relevant discussion section include this text:

Trogocytosis has been proposed as a mechanism for transferring energy-rich metabolites, including fatty acids, between interacting cells(Joly and Hudrisier 2003). In agreement with this idea, NK cells that accumulate lipid droplets use

these fatty acids to fuel β-oxidation and thereby support mitochondrial respiration. At the same time, macrophage-derived lipids suppress mTORC1 activity and consequently reduce IFN-γ production, but do not affect NK cell cytotoxicity. Inhibiting fatty-acid synthase (FASN) specifically in macrophages was sufficient both to prevent lipid accumulation in NK cells and to restore mTORC1 activity and IFN-γ output. Previous studies using rapamycin, a direct mTORC1 inhibitor, show that suppression of mTORC1 activity reduces IFN-γ production, as well as the expression of perforin and granzyme B, and can diminish cytotoxicity. However, in our system, although lipid-droplet accumulation in NK cells correlated with reduced mTORC1 activity, this reduction did not reach the level seen in rapamycin-treated controls. Correspondingly, macrophage-derived lipids suppressed IFN-γ, but did not impair cytotoxicity. While granzyme B and perforin expression were modestly reduced, NK cells still retained substantial levels of both proteins (well above FMO controls), consistent with their preserved killing capacity.

The manuscript would be improved with effector data from the in vivo-generated NK cells, but additional clarification from the authors in the discussion would suffice.

We thank the reviewer for this suggestion. We have amended the Discussion to clarify that the lipid-dependent regulation we describe does not impair NK-cell cytotoxic function. Instead, our data indicate that this pathway acts selectively as a brake on NK-cell IFN-γ-mediated inflammatory activity, while leaving their cytolytic capacity intact. This distinction is now explicitly highlighted in the revised text.

Reviewer #3 (Remarks to the Author): Expert on NK cells and metabolism

The study demonstrates that activated macrophages can transfer newly synthesized fatty acids to NK cells via direct cell–cell contact, leading to the suppression of IFN-γ production. However, several critical aspects of this mechanism warrant further investigation.

1. The mechanism underlying lipid transfer remains incompletely understood. Although the study proposes that CD36 is transferred to NK cells through trogocytosis, it reports that CD36 does not influence lipid accumulation in recipient NK cells. This raises questions about the actual pathway of fatty acid transfer-whether through extracellular vesicles (e.g., exosomes), membrane nanotubes, or direct membrane fusion-and the functional role of CD36 in this process. To address these uncertainties, live-cell imaging combined with super-resolution microscopy should be employed to dynamically visualize and track the cellular events associated with lipid transfer.

We thank the reviewer for highlighting this important point. Our data strongly argue against extracellular vesicles as the mode of transfer, as direct cell-cell contact is essential. We do not see anything when we use conditioned media or transwell assay. We show that transfer is abolished by inhibitors of actin remodelling, which is consistent with a trogocytosis-dependent or other contact dependent mechanism. Trogocytosis is a well-established process by which lymphocytes acquire membrane fragments and associated molecules from interacting cells, even though the precise ultrastructural steps remain incompletely defined.
While live-cell super-resolution imaging would certainly provide valuable mechanistic detail, our laboratory does not specialise in advanced trogocytosis imaging approaches, and resolving this question definitively is beyond the scope of the current study. We have therefore expanded the Discussion to emphasise that the precise structural mechanism remains an important avenue for future investigation.

2. The specific classes of fatty acids being transferred have not been definitively identified. A comprehensive lipidomic profiling approach is recommended to

characterize the molecular composition of the transferred lipids from macrophages to NK cells.

We thank the reviewer for this suggestion. We have now carried out comprehensive lipidomic profiling of NK cells with increased lipid droplets (Figure 4G-I). These analyses demonstrate that the elevated neutral lipid content is driven almost exclusively by triacylglycerols (TAGs) composed of palmitic acid (PA) and oleic acid (OA). These fatty acids correspond precisely to the products of de novo fatty-acid synthesis (PA) and its desaturation by SCD1 (OA).

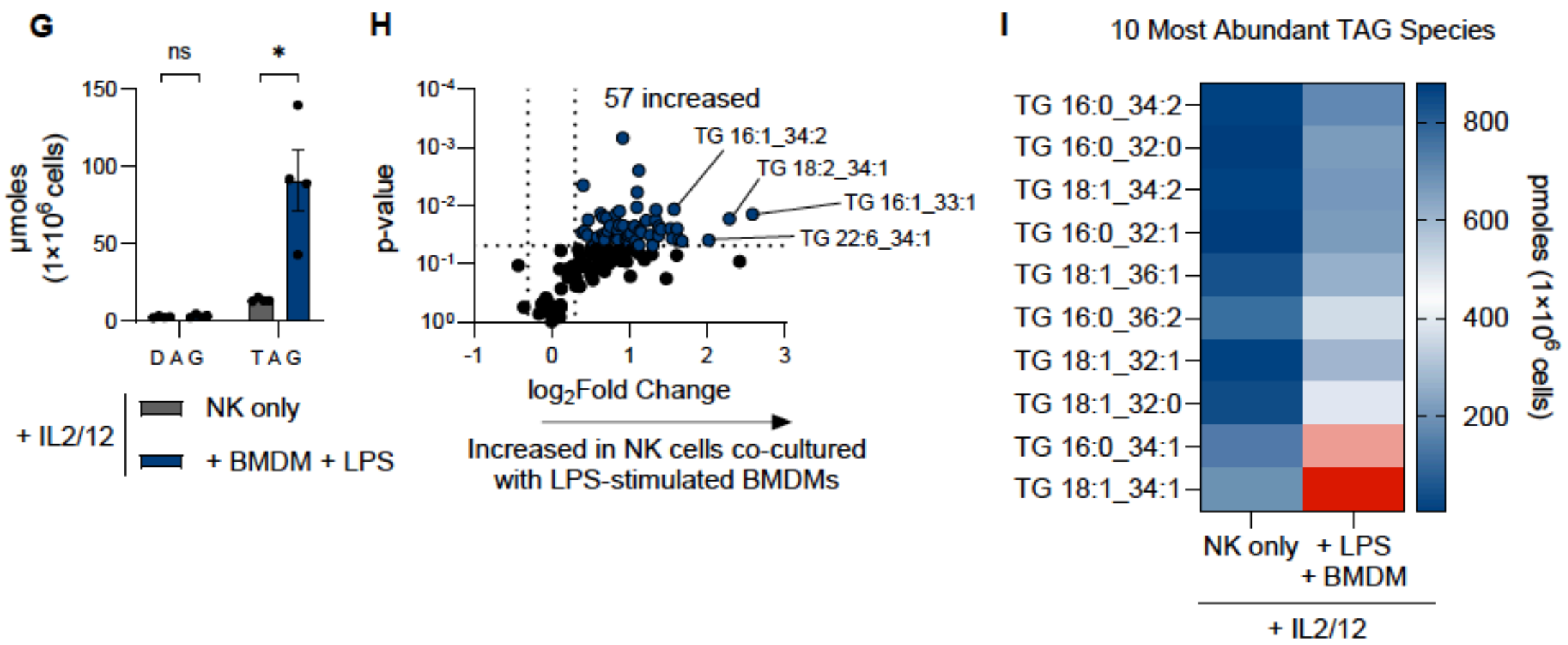


The remarkable dominance of PA/OA-containing TAG species strongly supports the conclusion that NK cells acquire newly synthesised fatty-acid-containing lipid species, most likely phospholipids generated in macrophages before TAG formation in NK cells. If the transfer involved bulk plasma-membrane fragments, we would expect a far broader range of chain lengths and saturation states reflective of the native macrophage phospholipid composition. Instead, the restricted FA profile points to a highly selective transfer of newly generated lipids. As noted in our responses to Reviewers 1 and 2, this interpretation is consistent with published work showing that macrophages can generate focal plasma-membrane domains enriched in newly synthesised phospholipids through targeted exocytosis from endosomal and ER-derived vesicles during phagocytosis and immune synapse formation. We have expanded the Discussion to integrate these findings and to clarify how focused delivery of nascent phospholipids could provide the substrate for direct capture by NK cells.

3. The assessment of NK cell function remains incomplete. Although IFN-γ production is significantly impaired, the effects on cytotoxic function-assessed via perforin and granzyme B expression-show inconsistent changes across experimental models, with no observed alteration in overall cytotoxicity. This apparent "functional decoupling" between cytokine production and cytolytic activity necessitates mechanistic explanation. Furthermore, the impact of lipid accumulation on NK cell viability, proliferation, and differentiation has not been evaluated. It is therefore essential to systematically examine markers of NK cell exhaustion, proliferative capacity, and the expression profiles of activating and inhibitory surface receptors.

We thank the reviewer for this broader question regarding NK-cell function and phenotype. We have now clarified these points in the revised manuscript.
First, NK-cell viability is not affected by co-culture with macrophages. As shown in Supplementary Figure 4A, NK cells remain uniformly viable and are recovered at similar numbers to the input population, arguing against substantial cell death or proliferation during the 18-hour culture period.

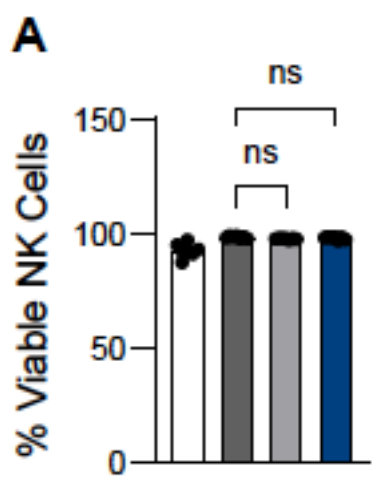


Second, we examined whether co-culture induces features of exhaustion or altered differentiation state. We assessed expression of PD-1 and TIGIT. There was very little PD-1 expression and while there was a statistically significant increase in BMDM co-cultured NK cells the % positive is too small to be meaningful. TIGIT expression was seen increased in activated NK cell but is not further increased with BMDM coculture. These data demonstrate that NK cells to not increase these features often associated with exhaustion.

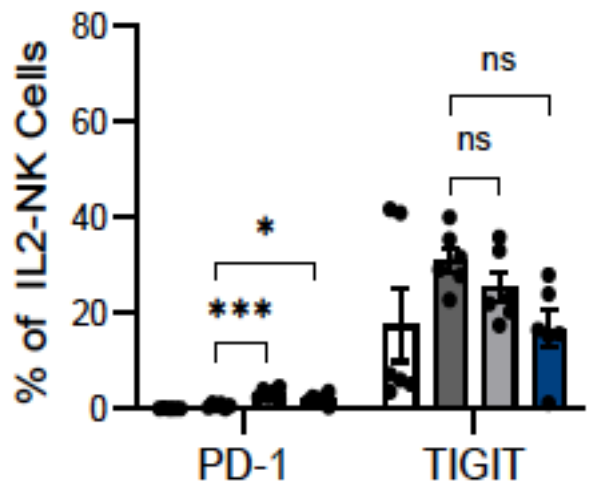


We also assessed markers associated with NK cell differentiation towards a ILC1-like phenotype including CD49a and CD200R.

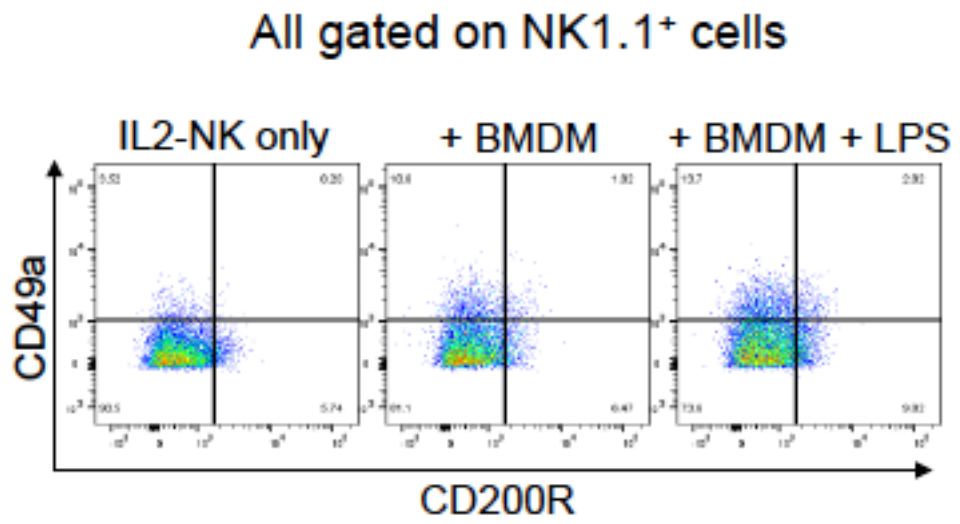


There was no significant change in expression of these ILC-like features while the expression of Eomes and T-bet (features of NK cells) remained strongly expressed. Il2-NK cocultured with BMDM on top (Supplementary Figure 3A) and spNK cocultures with peritoneal macrophages (Supplementary figure 3D) below.

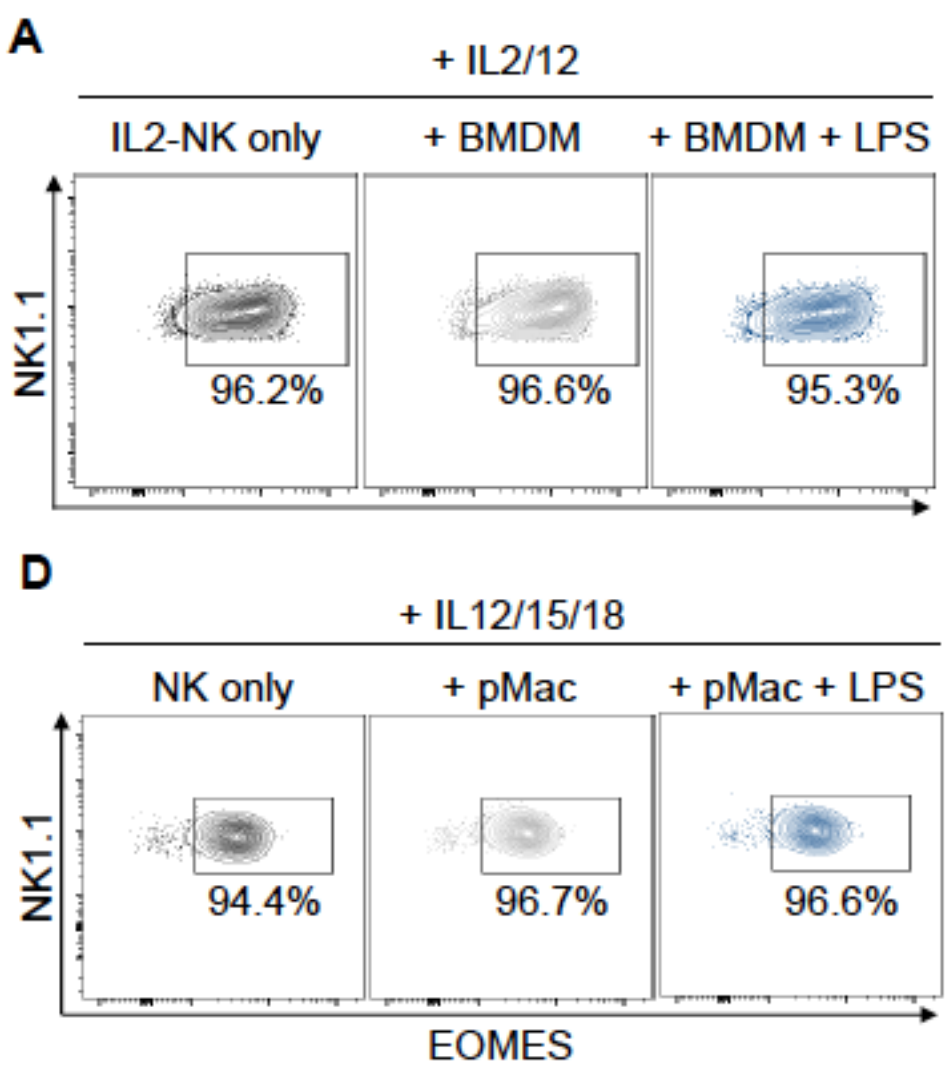


And Tbet data in Figure 3E (IL2-NK) and SplNK in Figure 3I

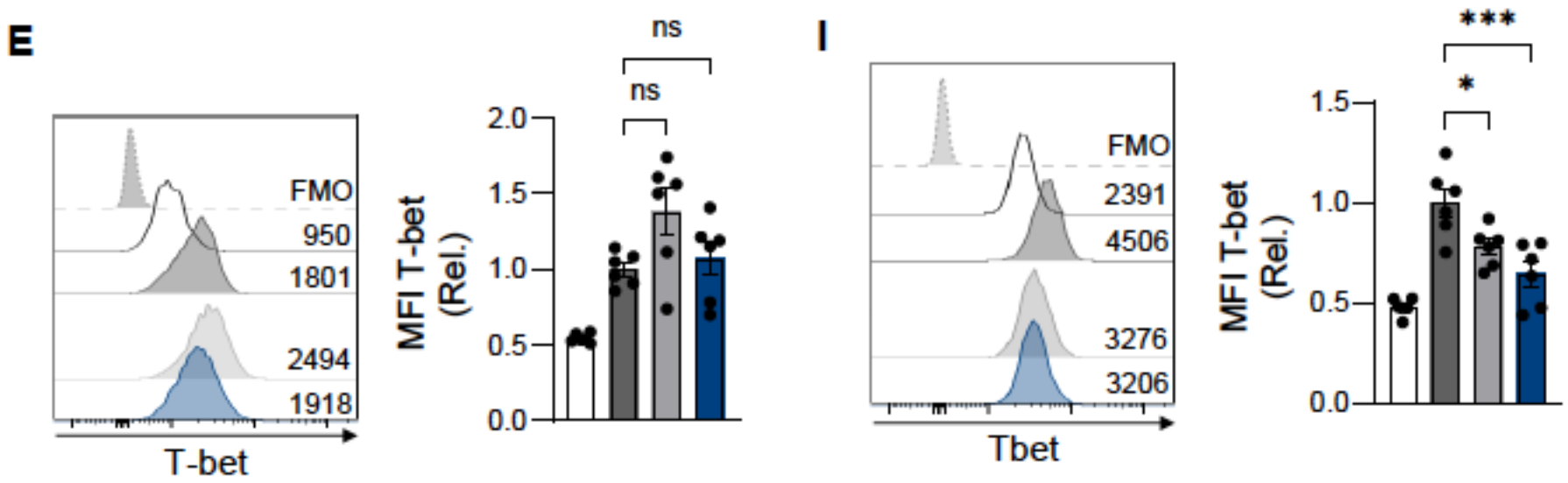


While there is a small decrease in Tbet in Spl-NK cells the expression of T-bet is still strongly above FMO. In addition, there was no increase in TGFbeta, the cytokine linked to the ILC-like differentiation programme, in LPS stimulated BMDM culture alone for 18h.

BMDMs cultured alone

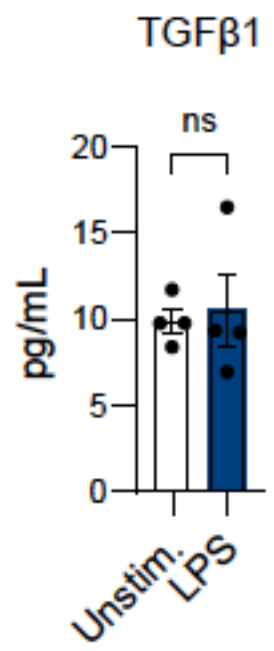


Taken together, the expression patterns of these markers are not consistent with ILC1-like differentiation or an exhausted phenotype. These data indicate that lipid accumulation does not drive a differentiation switch or functional exhaustion. As this is a complicated issue and the data is negative we would prefer to leave these data from the manuscript to avoid confusing the narrative.

Regarding cytotoxic function, although we observe modest reductions in granzyme B and perforin in some conditions, these molecules are extremely abundant in NK cells. A small decrease in expression does not necessarily translate into functional impairment. To clarify this point, we have now included FMO controls that demonstrate the substantial intracellular stores of perforin and granzyme B that remain after co-culture. These findings are consistent with the unchanged cytotoxic activity observed in our functional assays.

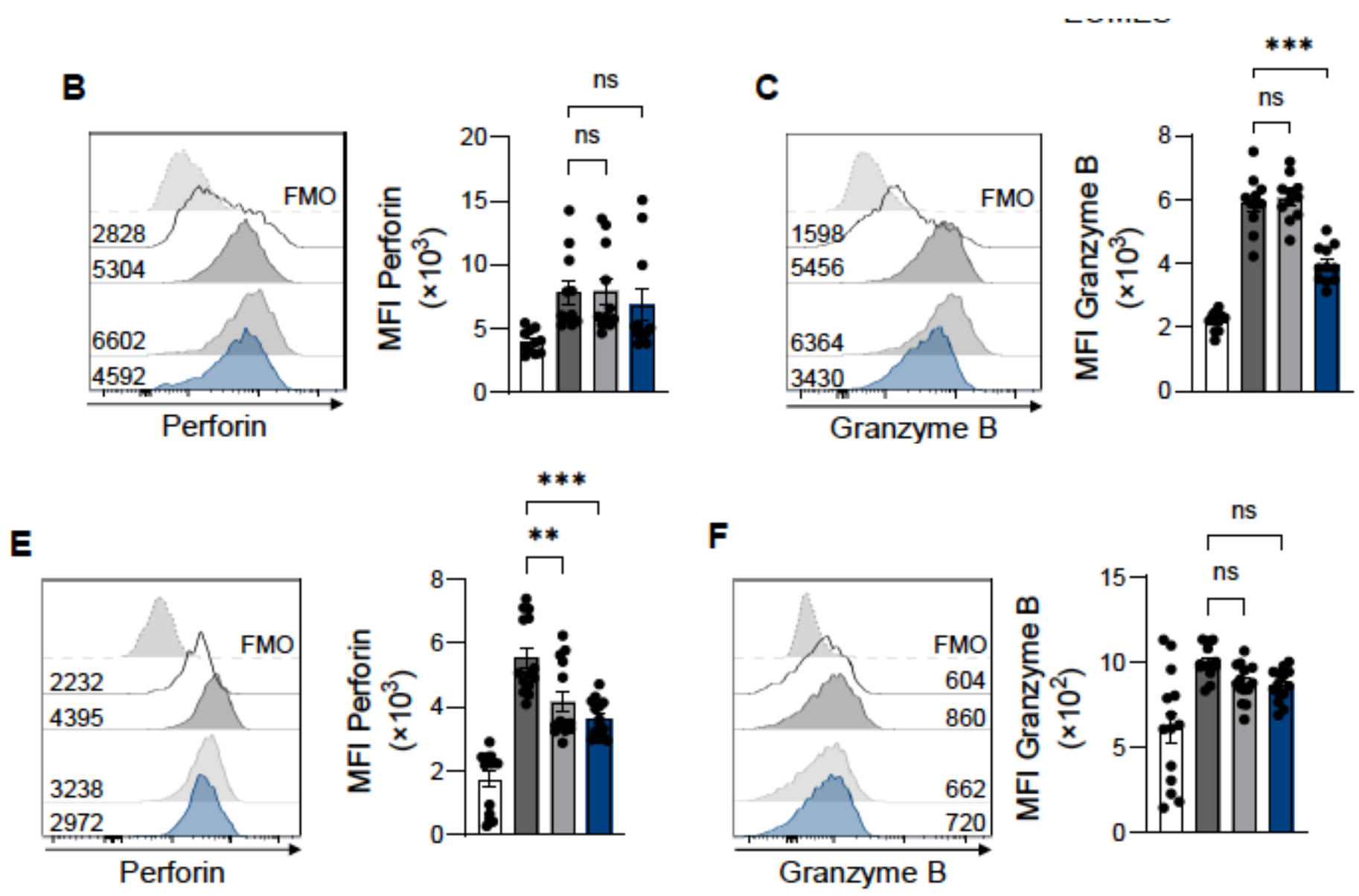


Finally, while we have not performed a full screen of activating and inhibitory NK-cell receptors, the preservation of cytotoxic function strongly suggests that core receptor pathways remain intact.

Collectively, these data reinforce the conclusion that lipid accumulation selectively impairs IFN-γ-mediated inflammatory function without affecting NK-cell viability, differentiation, or cytotoxic potential. We have incorporated these clarifications into the revised manuscript

4. The downstream mechanisms by which intracellular lipid accumulation suppresses IFN-γ production in NK cells remain unclear. Specifically, it is unknown whether accumulated lipids or lipid droplets interfere with key metabolic signaling pathways (e.g., mTOR, AMPK), mitochondrial bioenergetics, transcriptional regulation (e.g., STAT4 activation), or secretory machinery. To identify potential regulatory nodes, integrated omics analyses, including transcriptomics, metabolomics, or phosphoproteomics, should be performed on NK cells following co-culture with macrophages.

We thank the reviewer for raising this important point regarding the mechanism linking lipid accumulation to impaired IFN-γ production. We have expanded the manuscript to address this more clearly.

First, we examined several candidate pathways known to regulate IFN-γ transcription. Expression of T-bet, the key transcription factor for IFN-γ, was strongly expressed in NK cells with increased lipid droplets, indicating that reduced cytokine production is not driven by the loss of T-bet.

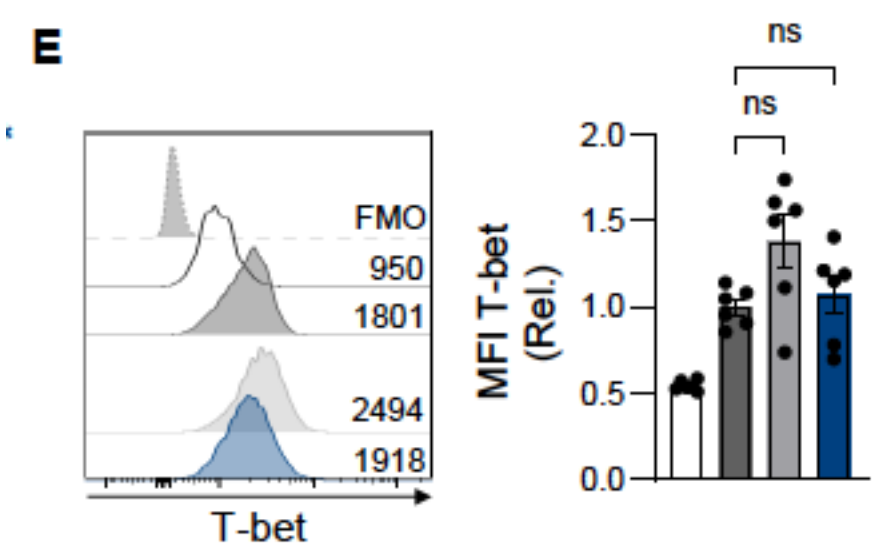


Loss of mitochondrial function has previously been linked to reduced IFNgamma. Therefore, we assessed mitochondrial stress and detected no increase in mitochondrial ROS, although NK cells with lipid droplets displayed a trend towards a reduction in a mitochondrial mass.

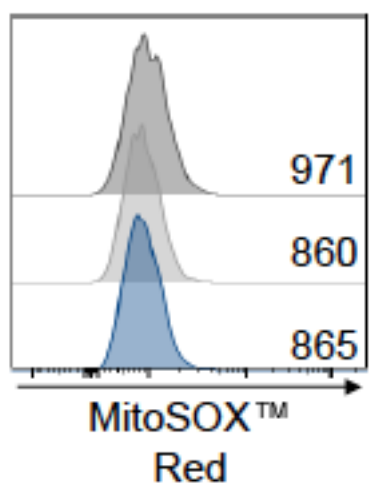


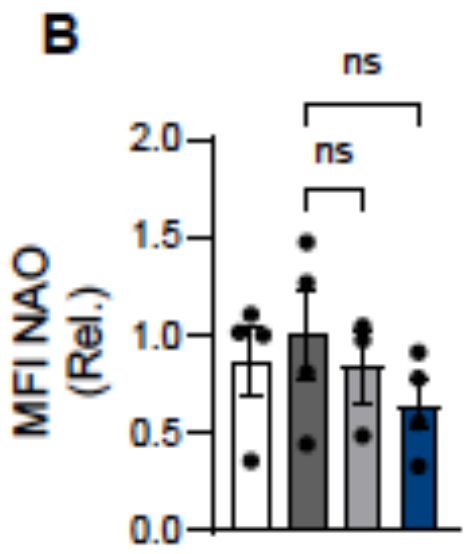


Importantly, we now provide new data demonstrating that mTORC1 activity is reduced in NK cells that accumulate lipids following co-culture (Figure 4B). This was assessed using phosphorylated S6 ribosomal protein as a readout, with rapamycin controls included to confirm specificity.

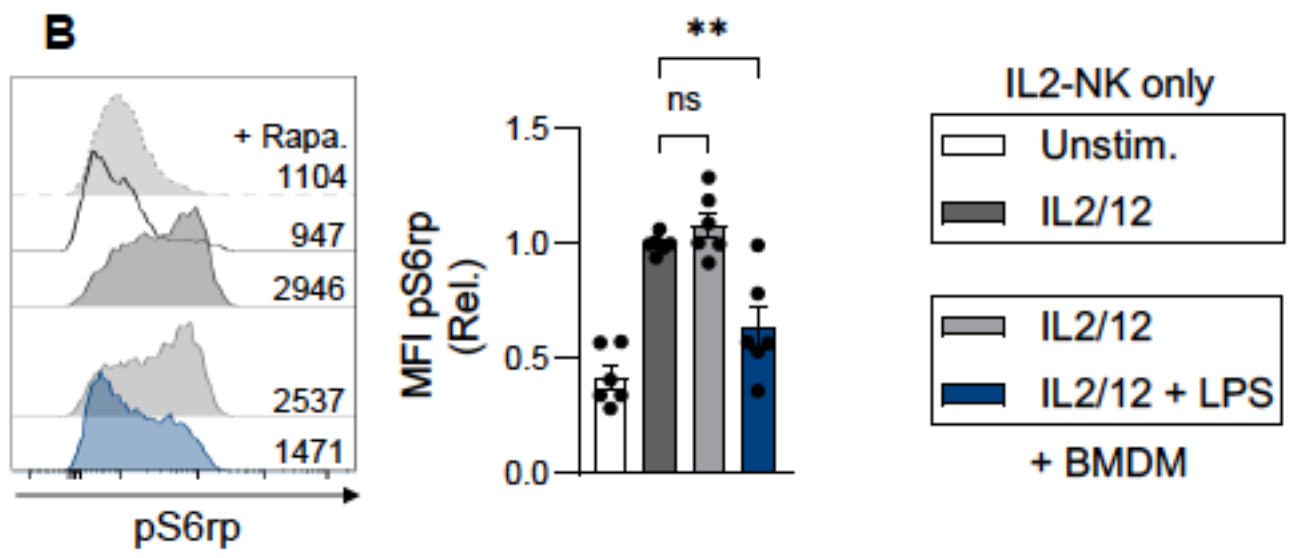


Interventions that prevent lipid-droplet formation in NK cells restore mTORC1 activity, supporting a functional link between lipid accumulation and suppression of this pathway. This is consistent with prior work from Lynch and colleagues showing that lipid overload can inhibit mTORC1 signalling in NK cells (Michelet, Dyck et al. 2018), and with multiple studies demonstrating that intact mTORC1 activity is essential for optimal IFN-γ production in NK cells (Donnelly, Loftus et al. 2014, Viel, Marcais et al. 2016, Zaiatz-Bittencourt, Jones et al. 2023).
Together, these data support a model in which intracellular lipid accumulation selectively dampens mTORC1 signalling, thereby limiting IFN-γ production without impairing cytotoxic function.
While we agree that integrated multi-omics approaches (transcriptomics, metabolomics, phosphoproteomics) would offer additional mechanistic insights, such comprehensive analyses are beyond the scope of the present study. We now explicitly acknowledge this in the revised Discussion.

5. The rationale for the selected co-culture duration requires clarification. Given that the duration of macrophage-NK cell interactions in vivo may be considerably shorter than those used in prolonged co-culture experiments, it is important to assess whether the observed phenotypic and functional changes reflect physiological conditions or are artifacts of extended in vitro exposure

We thank the reviewer for raising this point. Although we did not include these data in the manuscript, we have performed live-cell imaging of NK–macrophage co-cultures and observed stable, prolonged interactions between NK cells and macrophages rather than transient contacts. These observations align with what is known about immune-cell dynamics within tissue niches such as the peritoneal cavity, where leukocytes continuously migrate but frequently engage in sustained cell–cell contacts.

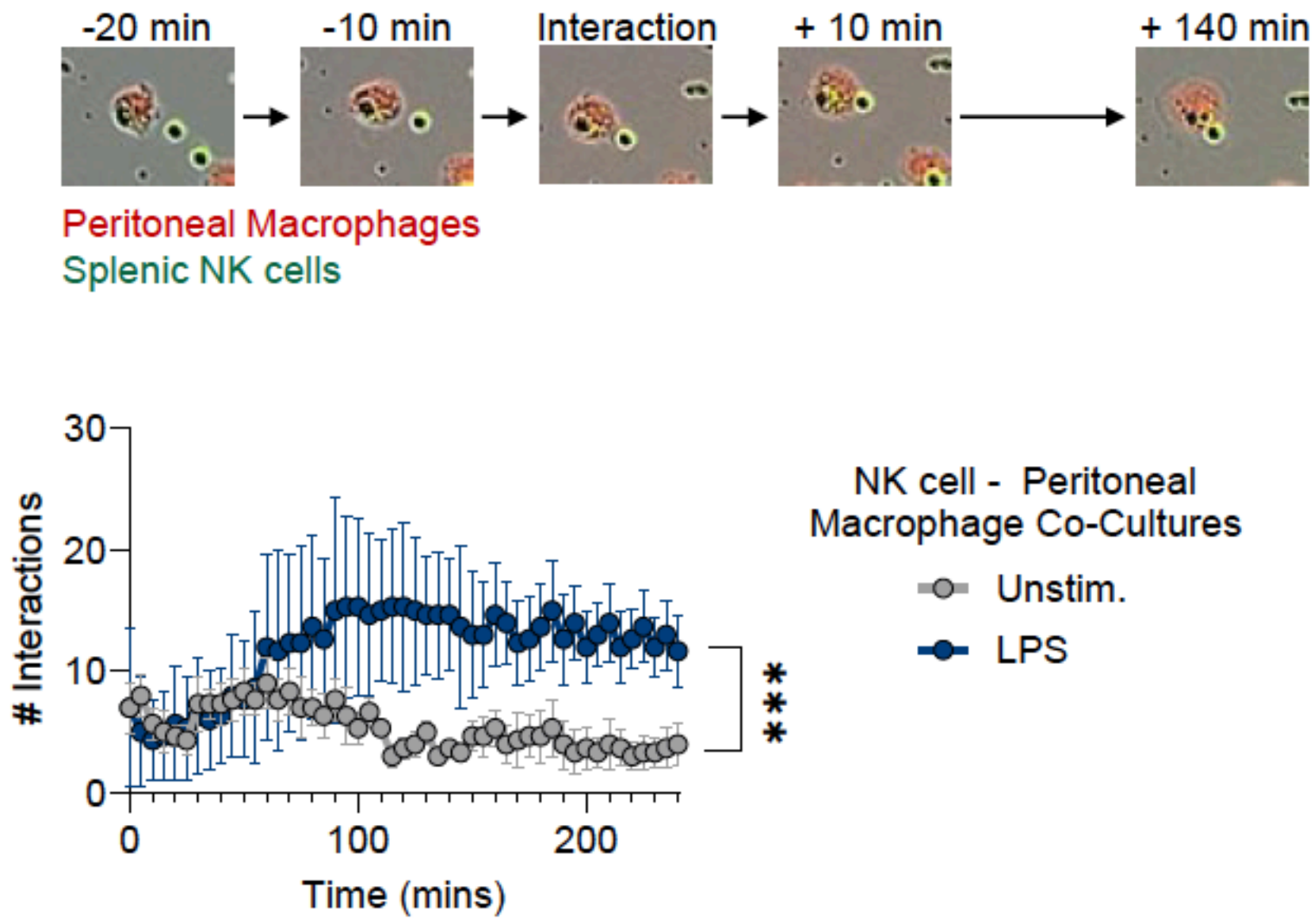


Moreover, intravital microscopy studies have shown that NK cells form long-lasting interactions with myeloid cells, including dendritic cells and macrophages, rather than merely scanning them transiently(Bajenoff, Breart et al. 2006, Barry, Hsu et al. 2018). These in vivo observations support the physiological relevance of the co-culture duration we used and indicate that NK cells can experience extended contact with myeloid cells during immune activation.

We have now clarified this rationale in the revised introduction and Discussion.

Introduction now reads:

"Cell-cell contact, whether through receptor engagement or synapse formation, is often required for maximal NK activation by macrophages(Tu, Bozorgzadeh et al. 2008, Lapaque, Walzer et al. 2009). Moreover, intravital imaging has shown that NK cells form sustained, sequential interactions with myeloid cells in vivo, highlighting significant opportunities for functional crosstalk(Bajenoff, Breart et al. 2006, Beuneu, Deguine et al. 2009). These sustained contacts can lead to the transfer of membrane fragments and associated proteins to NK cells through trogocytosis, with clear consequences for NK-cell phenotype(Reed, Reichelt et al. 2021). For example, NK-cell acquisition of MHC class II from dendritic cells generates MHCII$^{+}$ NK cells capable of suppressing CD4$^{+}$ T-cell response(Nakayama, Takeda et al. 2011)."

6. While pharmacological inhibition using C75 was employed to block fatty acid synthesis, genetic validation is lacking. The use of macrophage-specific fatty acid synthase (FASN) conditional knockout models would provide more definitive evidence supporting the role of de novo lipogenesis in mediating immunosuppressive effects on NK cells.

We appreciate the reviewer's suggestion regarding genetic validation using macrophage-specific FASN-deficient models. We did consider this approach. However, work from our institute has shown that macrophage-specific deletion of FASN substantially impairs TLR4 signalling and downstream responses to LPS (e.g., cytokine production, activation markers)(Carroll, Zaslona et al. 2018) Because our study relies on macrophage activation via TLR4 to generate the lipid-transfer phenotype, using FASN-deficient macrophages would introduce a major confounding variable. Specifically, it would be difficult to disentangle whether any observed effects on NK cells resulted from loss of de novo fatty-acid synthesis or from the broader defects in macrophage activation inherent to the FASN knockout.

For this reason, pharmacological inhibition (C75), which selectively blocks fatty-acid synthesis while preserving upstream TLR4 signalling, provides a clearer and more interpretable approach for this system. We now clarify this rationale in the revised Discussion.

We would like to thank the reviewers and editors once again for their thoughtful and constructive comments, which have substantially improved the clarity, depth, and impact of our manuscript. We have carefully addressed each point with new data, additional analyses, and expanded discussion where appropriate. We believe that these revisions significantly strengthen the mechanistic framework and robustness of our findings. We hope the revised manuscript now satisfactorily addresses all concerns and we remain grateful for the opportunity to further refine and improve our work.

All the best

David Finlay, PhD, F.T.C.D
Prof. in Immunometabolism
Trinity Biomedical Sciences Institute
Trinity St. James's Cancer Institute
Trinity College Dublin
Ireland

Bajenoff, M., B. Breart, A. Y. Huang, H. Qi, J. Cazareth, V. M. Braud, R. N. Germain and N. Glaichenhaus (2006). "Natural killer cell behavior in lymph nodes revealed by static and real-time imaging." J Exp Med **203**(3): 619-631.
Bajno, L., X. R. Peng, A. D. Schreiber, H. P. Moore, W. S. Trimble and S. Grinstein (2000). "Focal exocytosis of VAMP3-containing vesicles at sites of phagosome formation." J Cell Biol **149**(3): 697-706.
Barry, K. C., J. Hsu, M. L. Broz, F. J. Cueto, M. Binnewies, A. J. Combes, A. E. Nelson, K. Loo, R. Kumar, M. D. Rosenblum, M. D. Alvarado, D. M. Wolf, D. Bogunovic, N. Bhardwaj, A. I. Daud, P. K. Ha, W. R. Ryan, J. L. Pollack, B. Samad, S. Asthana, V. Chan and M. F. Krummel (2018). "A natural killer-dendritic cell axis defines checkpoint therapy-responsive tumor microenvironments." Nat Med **24**(8): 1178-1191.
Beuneu, H., J. Deguine, B. Breart, O. Mandelboim, J. P. Di Santo and P. Bousso (2009). "Dynamic behavior of NK cells during activation in lymph nodes." Blood **114**(15): 3227-3234.
Biron, C. A., K. B. Nguyen, G. C. Pien, L. P. Cousens and T. P. Salazar-Mather (1999). "Natural killer cells in antiviral defense: function and regulation by innate cytokines." Annu Rev Immunol **17**: 189-220.
Carroll, R. G., Z. Zaslona, S. Galvan-Pena, E. L. Koppe, D. C. Sevin, S. Angiari, M. Triantafilou, K. Triantafilou, L. K. Modis and L. A. O'Neill (2018). "An unexpected link between fatty acid synthase and cholesterol synthesis in proinflammatory macrophage activation." J Biol Chem **293**(15): 5509-5521.
Cox, D., D. J. Lee, B. M. Dale, J. Calafat and S. Greenberg (2000). "A Rab11-containing rapidly recycling compartment in macrophages that promotes phagocytosis." Proc Natl Acad Sci U S A **97**(2): 680-685.
Di, A., D. J. Nelson, V. Bindokas, M. E. Brown, F. Libunao and H. C. Palfrey (2003). "Dynamin regulates focal exocytosis in phagocytosing macrophages." Mol Biol Cell **14**(5): 2016-2028.

Donnelly, R. P., R. M. Loftus, S. E. Keating, K. T. Liou, C. A. Biron, C. M. Gardiner and D. K. Finlay (2014). "mTORC1-dependent metabolic reprogramming is a prerequisite for NK cell effector function." J Immunol **193**(9): 4477-4484.
Freeman, S. A. and S. Grinstein (2014). "Phagocytosis: receptors, signal integration, and the cytoskeleton." Immunol Rev **262**(1): 193-215.
Gaus, K., M. Rodriguez, K. R. Ruberu, I. Gelissen, T. M. Sloane, L. Kritharides and W. Jessup (2005). "Domain-specific lipid distribution in macrophage plasma membranes." J Lipid Res **46**(7): 1526-1538.
Joly, E. and D. Hudrisier (2003). "What is trogocytosis and what is its purpose?" Nat Immunol **4**(9): 815.
Kanevskiy, L. M., S. A. Erokhina, M. A. Streltsova, W. G. Telford, A. M. Sapozhnikov and E. I. Kovalenko (2014). "Bacterial lipopolysaccharide activates CD57-negative human NK cells." Biochemistry (Mosc) **79**(12): 1339-1348.
Kanevskiy, L. M., S. A. Erokhina, M. A. Streltsova, R. H. Ziganshin, W. G. Telford, A. M. Sapozhnikov and E. I. Kovalenko (2019). "The Role of O-Antigen in LPS-Induced Activation of Human NK Cells." J Immunol Res **2019**: 3062754.
Lapaque, N., T. Walzer, S. Meresse, E. Vivier and J. Trowsdale (2009). "Interactions between human NK cells and macrophages in response to Salmonella infection." J Immunol **182**(7): 4339-4348.
Li, L., Z. Zeng, Z. Qi, X. Wang, X. Gao, H. Wei, R. Sun and Z. Tian (2015). "Natural Killer Cells-Produced IFN-gamma Improves Bone Marrow-Derived Hepatocytes Regeneration in Murine Liver Failure Model." Sci Rep **5**: 13687.
Lin, Q., L. Rong, X. Jia, R. Li, B. Yu, J. Hu, X. Luo, S. R. Badea, C. Xu, G. Fu, K. Lai, M. C. Lee, B. Zhang, H. Gong, N. Zhou, X. L. Chen, S. H. Lin, G. Fu and J. D. Huang (2021). "IFN-gamma-dependent NK cell activation is essential to metastasis suppression by engineered Salmonella." Nat Commun **12**(1): 2537.
Michelet, X., L. Dyck, A. Hogan, R. M. Loftus, D. Duquette, K. Wei, S. Beyaz, A. Tavakkoli, C. Foley, R. Donnelly, C. O'Farrelly, M. Raverdeau, A. Vernon, W. Pettee, D. O'Shea, B. S. Nikolajczyk, K. H. G. Mills, M. B. Brenner, D. Finlay and L. Lynch (2018). "Metabolic reprogramming of natural killer cells in obesity limits antitumor responses." Nat Immunol **19**(12): 1330-1340.
Nakayama, M., K. Takeda, M. Kawano, T. Takai, N. Ishii and K. Ogasawara (2011). "Natural killer (NK)-dendritic cell interactions generate MHC class II-dressed NK cells that regulate CD4+ T cells." Proc Natl Acad Sci U S A **108**(45): 18360-18365.
Reed, J., M. Reichelt and S. A. Wetzel (2021). "Lymphocytes and Trogocytosis-Mediated Signaling." Cells **10**(6).
Scharton, T. M. and P. Scott (1993). "Natural killer cells are a source of interferon gamma that drives differentiation of CD4+ T cell subsets and induces early resistance to Leishmania major in mice." J Exp Med **178**(2): 567-577.

Souza-Fonseca-Guimaraes, F., M. Parlato, F. Philippart, B. Misset, J. M. Cavaillon, M. Adib-Conquy and g. Captain study (2012). "Toll-like receptors expression and interferon-gamma production by NK cells in human sepsis." Crit Care **16**(5): R206.

Tu, Z., A. Bozorgzadeh, R. H. Pierce, J. Kurtis, I. N. Crispe and M. S. Orloff (2008). "TLR-dependent cross talk between human Kupffer cells and NK cells." J Exp Med **205**(1): 233-244.

Viel, S., A. Marcais, F. S. Guimaraes, R. Loftus, J. Rabilloud, M. Grau, S. Degouve, S. Djebali, A. Sanlaville, E. Charrier, J. Bienvenu, J. C. Marie, C. Caux, J. Marvel, L. Town, N. D. Huntington, L. Bartholin, D. Finlay, M. J. Smyth and T. Walzer (2016). "TGF-beta inhibits the activation and functions of NK cells by repressing the mTOR pathway." Sci Signal **9**(415): ra19.

Vivier, E., D. H. Raulet, A. Moretta, M. A. Caligiuri, L. Zitvogel, L. L. Lanier, W. M. Yokoyama and S. Ugolini (2011). "Innate or adaptive immunity? The example of natural killer cells." Science **331**(6013): 44-49.

Wright, S. D. and S. C. Silverstein (1984). "Phagocytosing macrophages exclude proteins from the zones of contact with opsonized targets." Nature **309**(5966): 359-361.

Zaiatz-Bittencourt, V., F. Jones, M. Tosetto, C. Scaife, G. Cagney, E. Jones, G. A. Doherty and E. J. Ryan (2023). "Butyrate limits human natural killer cell effector function." Sci Rep **13**(1): 2715.

23rd June 2026

Dear Reviewer 3,

I would like to thank you for your comments. Please find a point by point response to your last 2 comments in red.

1.Future studies could further validate the downstream mechanisms by which intracellular lipid accumulation inhibits IFN-γ production in NK cells. Previous research has shown that upon uptake of lipids from macrophages, NK cells exhibit reduced mTORC1 activity, which ultimately suppresses IFN-γ production. Whether the inhibition of IFN secretion induced by lipid accumulation in NK cells can be reversed via mTORC1 activation warrants investigation.

We thank the reviewer for this insightful suggestion. We have expanded the Discussion to further consider the mechanisms by which intracellular lipid accumulation may suppress mTORC1 activity and IFN-γ production in NK cells. In particular, we now outline two non-mutually exclusive models involving (i) altered lysosomal signalling following membrane uptake and (ii) ER stress responses associated with lipid droplet biogenesis. Importantly, we highlight that inhibition of fatty acid synthesis in macrophages restores both mTORC1 activity and IFN-γ production in NK cells, indicating that this pathway is reversible through upstream metabolic intervention.

We agree that direct testing of whether mTORC1 activation can fully reverse NK cell dysfunction would be highly informative. However, such experiments would require additional genetic or pharmacological manipulation of mTORC1 signalling combined with temporal metabolic analysis, which are beyond the scope of a minor revision.

We therefore discuss this as an important avenue for future investigation.

“Our findings position mTORC1 as a key node linking macrophage-derived lipid transfer to suppression of NK cell function. While mTORC1 inhibition is typically associated with nutrient limitation, our data support a model in which lipid accumulation contributes directly to mTORC1 attenuation. Importantly, inhibition of fatty acid synthesis in macrophages restored both mTORC1 activity and IFN-γ production, demonstrating functional reversibility through upstream metabolic intervention. The mechanisms underlying this effect remain to be defined but are likely multifactorial. Uptake of membrane material via contact-dependent transfer may increase endo-lysosomal flux, potentially perturbing lysosomal function; lysosomal stress is suggested as a mechanism for the dislocation of mTORc1 from the lysosome, hence mTORc1 inhibition(Lakpa, Khan et al. 2021, Saftig and Puertollano 2021). In parallel, rapid lipid droplet formation is consistent with ER lipid buffering and stress responses, which can suppress mTORC1(Guan, Krokowski et al. 2014, Jarc and Petan 2019, Olzmann and Carvalho 2019).”

2. The article proposes that NK cells and macrophages form sustained interactions, and such direct crosstalk indeed plays a critical role in regulating NK cell function. Determining the optimal contact duration between NK cells and macrophages via dynamic monitoring may enhance the scientific rigor of experimental design.

We additionally expanded the Discussion to address the temporal aspects of NK cell–macrophage interactions. Our data already provide initial insight into the kinetics of this process, including rapid CD36 acquisition, progressive lipid accumulation, and delayed suppression of IFN-γ production. We now highlight the importance of future studies using live-cell imaging and dynamic approaches to define the duration and frequency of cell-cell interactions required for metabolic regulation.
We agree that defining the precise duration and frequency of NK cell-macrophage interactions would strengthen mechanistic understanding. However, this would

require high-resolution live-cell imaging and quantitative tracking approaches that are not feasible within the scope of a minor revision.
We therefore frame this as an important direction for future work.

“Our data provides insight into the temporal dynamics of NK cell-macrophage crosstalk. We observe rapid acquisition of CD36 by NK cells within 1 hours of co-culture, showing rapid onset of trogocytosis. Inflammatory macrophages initially enhance NK cell IFN-γ production, followed by suppression coinciding with the emergence of lipid accumulation in NK cells. These findings support a temporal sequence in which early activating signals are progressively overridden by contact-dependent metabolic regulation. While this framework defines the kinetics of the interaction, our system does not resolve the precise duration or frequency of contact required to drive lipid transfer and functional suppression. Addressing this will require high-resolution live-cell imaging and dynamic tracking approaches to quantify interaction times and link these to metabolic and functional outcomes at the single-cell level.”

Kind regards

Dr. David Finlay
Prof. In, Immunometabolism
Trinity Biomedical Sciences Institute
Trinity College Dublin
Ireland

Guan, B. J., D. Krokowski, M. Majumder, C. L. Schmotzer, S. R. Kimball, W. C. Merrick, A. E. Koromilas and M. Hatzoglou (2014). "Translational control during

endoplasmic reticulum stress beyond phosphorylation of the translation initiation factor eIF2alpha." J Biol Chem **289**(18): 12593-12611.
Jarc, E. and T. Petan (2019). "Lipid Droplets and the Management of Cellular Stress." Yale J Biol Med **92**(3): 435-452.
Lakpa, K. L., N. Khan, Z. Afghah, X. Chen and J. D. Geiger (2021). "Lysosomal Stress Response (LSR): Physiological Importance and Pathological Relevance." J Neuroimmune Pharmacol **16**(2): 219-237.
Olzmann, J. A. and P. Carvalho (2019). "Dynamics and functions of lipid droplets." Nat Rev Mol Cell Biol **20**(3): 137-155.
Saftig, P. and R. Puertollano (2021). "How Lysosomes Sense, Integrate, and Cope with Stress." Trends Biochem Sci **46**(2): 97-112.
